# Ultrafast Photophysics of Ni(I)–Bipyridine Halide Complexes: Spanning the Marcus Normal and Inverted Regimes

**DOI:** 10.1021/jacs.4c04091

**Published:** 2024-05-22

**Authors:** Erica Sutcliffe, David A. Cagan, Ryan G. Hadt

**Affiliations:** Division of Chemistry and Chemical Engineering, Arthur Amos Noyes Laboratory of Chemical Physics, California Institute of Technology, Pasadena, California 91125, United States

## Abstract

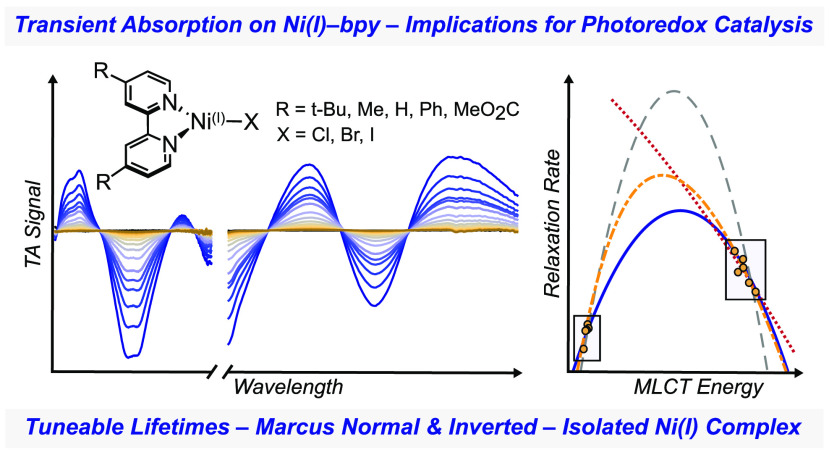

Owing to their light-harvesting properties, nickel–bipyridine (bpy) complexes have found wide use in metallaphotoredox cross-coupling reactions. Key to these transformations are Ni(I)–bpy halide intermediates that absorb a significant fraction of light at relevant cross-coupling reaction irradiation wavelengths. Herein, we report ultrafast transient absorption (TA) spectroscopy on a library of eight Ni(I)–bpy halide complexes, the first such characterization of any Ni(I) species. The TA data reveal the formation and decay of Ni(I)-to-bpy metal-to-ligand charge transfer (MLCT) excited states (10–30 ps) whose relaxation dynamics are well described by vibronic Marcus theory, spanning the normal and inverted regions as a result of simple changes to the bpy substituents. While these lifetimes are relatively long for MLCT excited states in first-row transition metal complexes, their duration precludes excited-state bimolecular reactivity in photoredox reactions. We also present a one-step method to generate an isolable, solid-state Ni(I)–bpy halide species, which decouples light-initiated reactivity from dark, thermal cycles in catalysis.

## 1 Introduction

**Figure 1:**
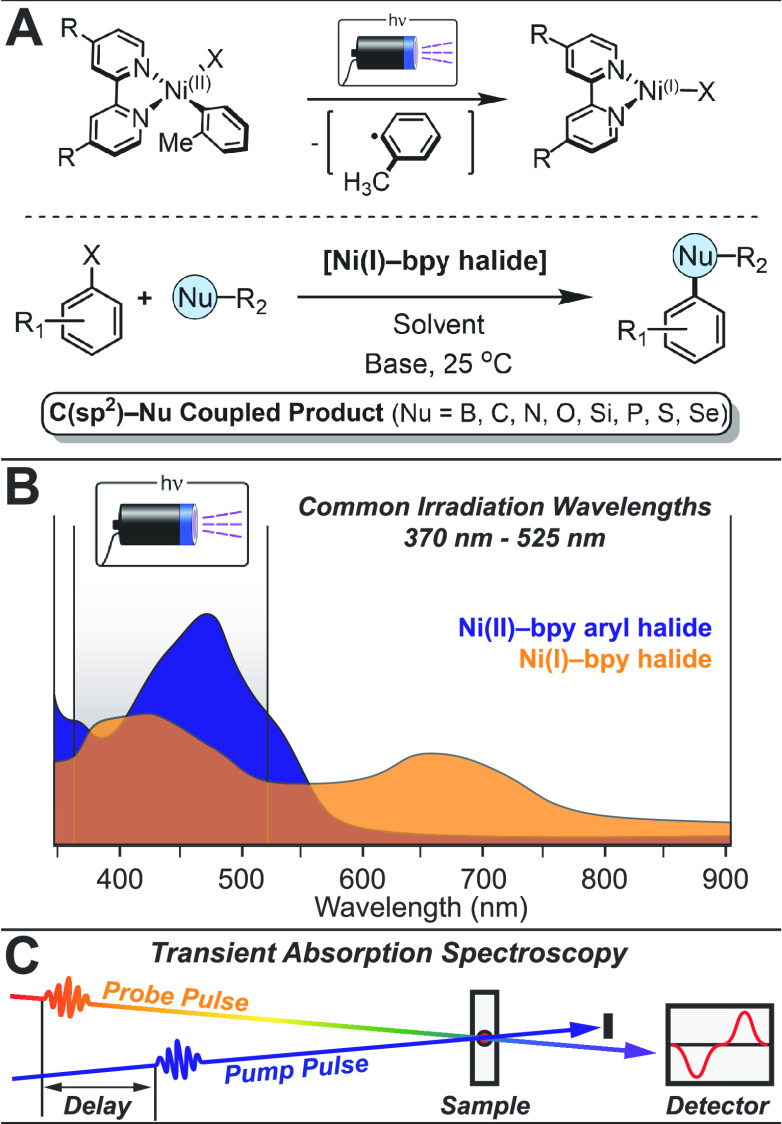
(A) Photogeneration and cross-coupling reactivity of Ni(I)–bipyridine halides. (B) Comparison of Ni(II)(^*t*-Bu^bpy)(*o*-tolyl)Cl and photogenerated Ni(I)(^*t*-Bu^bpy)Cl, illustrating their overlapping absorbance profiles. (C) Schematic of pump–probe transient absorption spectroscopy.

Ni(II)–bipyridine (bpy) aryl halide complexes are widely used for their ability to facilitate light-driven cross-coupling reactions.^1−12^ Excited-state Ni(II)–C(aryl) bond homolysis results in the formation of low-valent Ni(I)–bpy halide species^13−15^ that can activate aryl halides through a subsequent dark catalytic cycle. While aryl iodides and bromides can be activated with relative ease, increasing the electron-donating ability of the bpy substituents allows for activation of stronger C(sp^2^)–Cl bonds.^16−24^ Coupling light reactions for *in situ* Ni(I) generation to dark reactions for C–X bond activation enables Ni(I)/(III) catalytic cycles in the presence of nucleophilic coupling partners (e.g., amines, alcohols, thiols, etc., Figure 1A).^25−30^ Indeed, Ni(I)–bpy halide species have been shown to facilitate cross-coupling with remarkable scope, forging new C(sp^2^)–X bonds in good-to-excellent yields.^31^

As such, recent efforts have been made to understand the properties and reactivity profiles of Ni(I)–bpy halide complexes. However, they are prone to dimerization at elevated temperatures or high concentrations and exhibit rapid decomposition in the presence of oxygen or water, making their direct structural and spectroscopic characterization challenging.^24,32−34^ Ni(I)–bpy species can be stabilized through steric protection or backbonding to coordinating olefins.^35,36^ While these routes provided some of the first X-ray crystal structures of Ni(I)–bpy complexes, the stabilization slows or fully inhibits aryl halide oxidative addition. Alternatively, *in situ* formation of Ni(I)–bpy complexes has allowed for detailed mechanistic analysis. Pulsed radiolysis and electrochemical methods identified a Ni(I)(^*t*-Bu^bpy)Br species and have provided rate constants for aryl iodide oxidative addition.^21,37^ Solid-state polynuclear Ni species (e.g., [Ni(I)(^EtO_2_C^bpy)Cl]_*n*_, *n* = 2 or 4) are precursors to monomeric Ni(I) and did likewise for aryl bromides.^19^ From Hammett analysis, both of these aryl halide classes are thought to be activated by a concerted two-electron oxidative addition. Additionally, air- and moisture-free irradiation of parent Ni(II) complexes leads to stoichiometric conversion to Ni(I)–bpy halide photoproducts, many of which activate aryl chlorides.^24^ Analogous Hammett analysis demonstrated a nearly 2-fold increase in ρ-value relative to aryl bromides or iodides, suggesting a two-electron nucleophilic aromatic substitution (S_N_Ar) mechanism for the activation of the C(sp^2^)–Cl bond.^24^

Interestingly, all the Ni(I)–bpy species observed thus far absorb light across a wide wavelength range with similar or greater extinction coefficients than their parent Ni(II) complexes.^24^ More generally, many of the previously proposed metallaphotoredox reactions invoke photon absorption by specific Ni species and/or photosensitizers, indicating that *in situ* generated intermediates also absorb a significant fraction of photons during LED irradiation (Figure 1B).

To date, there have been no direct studies of the photophysical properties of Ni(I) intermediates that form as part of metallaphotoredox catalytic cycles. Given the importance of light-activation for Ni(II), Ni(I) excited states may also participate in processes that could influence catalysis. Furthermore, there has been significant interest in understanding geometric and electronic structural factors that contribute to charge transfer excited-state lifetimes in first-row transition metal complexes, including recent work on Ni(II).^14,18,38−40^

Herein, we have utilized ultrafast transient absorption (TA) spectroscopy (Figure 1C) to study a library of photogenerated Ni(I)(^R^bpy)X species (R = *t*-Bu, Me, H, Ph, MeO_2_C; X = Cl, Br, I) and an independently isolated Ni(I)(^MeO_2_C^bpy)Cl complex, the first such photophysical study for any Ni(I) species. The TA data are consistent with the formation and decay of ^2^Ni(I)-to-bpy metal-to-ligand charge transfer (^2^MLCT) excited states with lifetimes ranging from ∼10–30 ps. We also find the bpy substituent strongly influences the mechanism of ^2^MLCT excited-state deactivation. Tuning from electron-donating to electron-withdrawing substituents has provided a rare example of a series of related complexes whose excited-state deactivation processes span the Marcus normal and inverted regimes.

## 2 Results

### 2.1 Steady State and Ultrafast UV–Vis–NIR Spectroscopy

**Figure 2:**
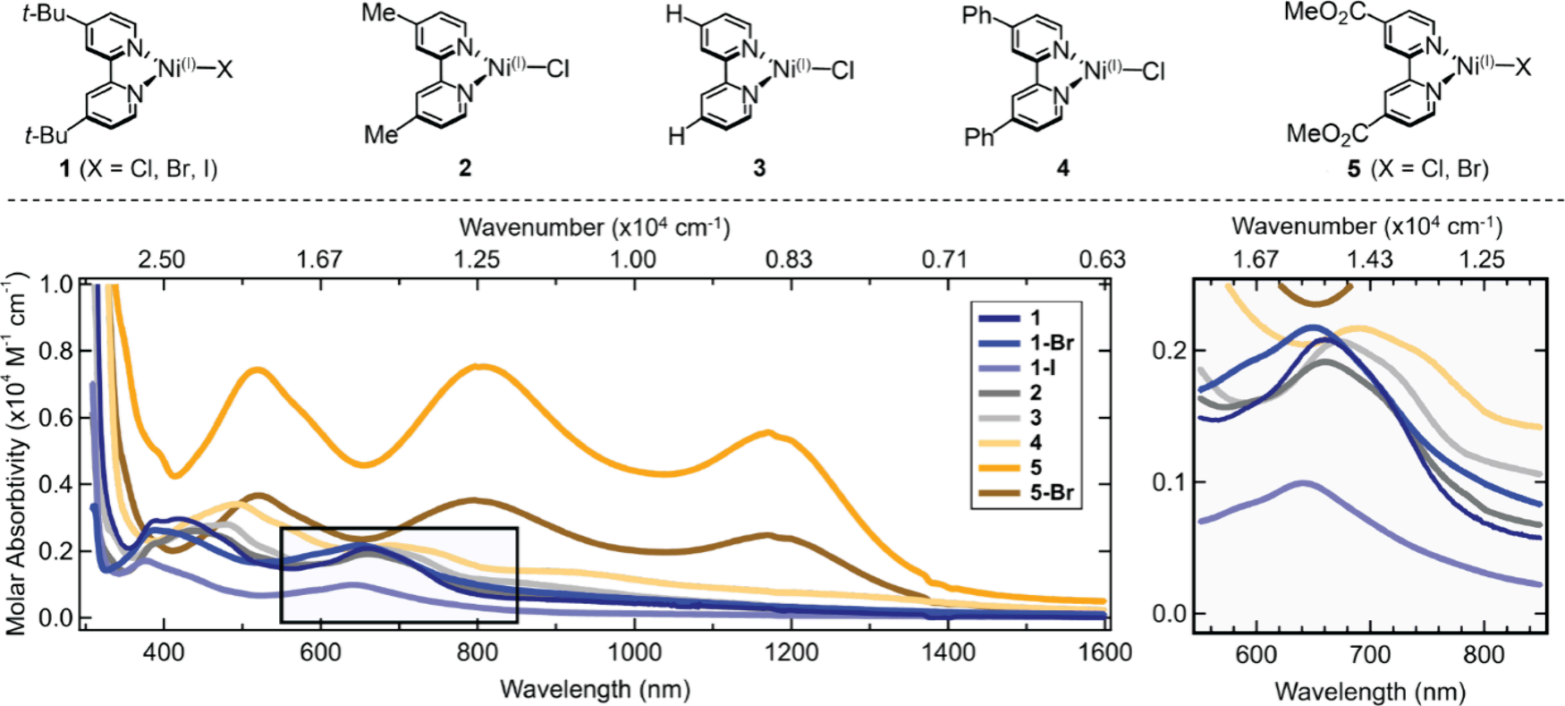
Structures and UV–vis–NIR absorption spectra of the photochemically generated Ni(I)–bpy halides in THF. Boxed section is expanded on the right. Analogous figure with wavenumber axis is given as Figure S9.

Following our previous work,^24^ Ni(I)(^R^bpy)X complexes were photogenerated from Ni(II)(^R^bpy)(*o-*tolyl)X parents (R = *t*-Bu, Me, Ph, H, MeO_2_C; X = Cl, Br, I) (**1**–**5**, Figure 2 and Figures S7–S8). Complete synthetic details are provided in the Supporting Information, Section S1.2. In general, Ni(II) complexes were dissolved in tetrahydrofuran (THF) and irradiated under air- and moisture-free conditions using purple or blue PR160L Kessil LEDs (370 or 390 nm incident light) to afford >95% Ni(II)(^R^bpy)(*o-*tolyl)X to Ni(I)(^R^bpy)X conversion.

UV–vis–NIR absorption spectra of **1**–**5** feature transitions with molar absorptivity on the order of 10^3^ M^–1^ cm^–1^ (Figure 2), with minor energy shifts upon variation of the halide; more dramatic shifts are observed upon modifications of the bpy substituents. As discussed in the Supporting Information, Section S1.4, the absorption bands can be assigned as Ni(I)-to-bpy MLCTs, consistent with our previous work.^24^ Density functional theory (DFT) calculated molecular orbital diagrams are given in Figures S34–S41, and time-dependent DFT (TD-DFT) predicted absorption transitions are tabulated in the Supporting Information, Section S2.4.

We have used ultrafast TA spectroscopy to elucidate the photophysical properties of the Ni(I) intermediates. TA spectra for all compounds were measured in THF using either 700 or 800 nm pump pulses. While some residual Ni(II) precursor can be present in solution due to the *in situ* generation of **1**–**5**, the precursors do not absorb light near the 700 or 800 nm excitation.^14,15^ Nevertheless, additional pump wavelengths were also used to determine the effect of pumping alternative, higher energy transitions. Full details of the experimental setup and subsequent analysis are given in the Supporting Information, Sections S1.1 and S1.5.

Similar to their Ni(II) precursors, all Ni(I) compounds showed a sizable ultrafast TA response. While the Ni(II) precursors exhibit ^3^d-d lifetimes of several nanoseconds,^14,18^ Ni(I) excited states decay more rapidly, with no measurable signal after a few hundred ps. Difference spectra for the longest-lived state of each compound (Figure S32) exhibit ground state bleach (GSB) signals that clearly align with the MLCT bands in the Ni(I) steady state absorption, along with significant excited state absorption (ESA) in the UV–visible region; complexes **4** and **5** also show sizable signal in the NIR region (Figures S21 and S31).

Changing the Cl ligand in **1** to Br (**1-Br**) or to I (**1-I**) slightly blue-shifts the MLCT bands in the ground-state absorption spectra (∼160 and 310 cm^–1^, respectively). TA spectra for these halide variants were similar to **1**, but with a small increase in lifetime of the long-lived state going down the group. Substituting Cl in **5** for Br (**5-Br**) also gave a small change in the TA spectra, but with the reverse trend; the long-lived state showed a slight decrease in lifetime for Br versus Cl. Thus, the halide has a minor but measurable impact on the relaxation kinetics. Five bpy variants were also investigated (**1**–**5**). Compounds **1**–**3** have comparable absorption spectra; these similarities are reflected in their ultrafast behavior, with all three compounds exhibiting decay pathways with similar time constants. In contrast, **4** and **5** showed a longer-lived three-component decay pathway. Notably, the lowest-energy MLCT bands of **4** and **5** extend into the NIR, while the lowest-energy MLCTs of complexes **1**–**3** are located in the visible. Thus, the bpy substituents significantly perturb the steady state electronic structure and the excited-state relaxation kinetics.

**Figure 3:**
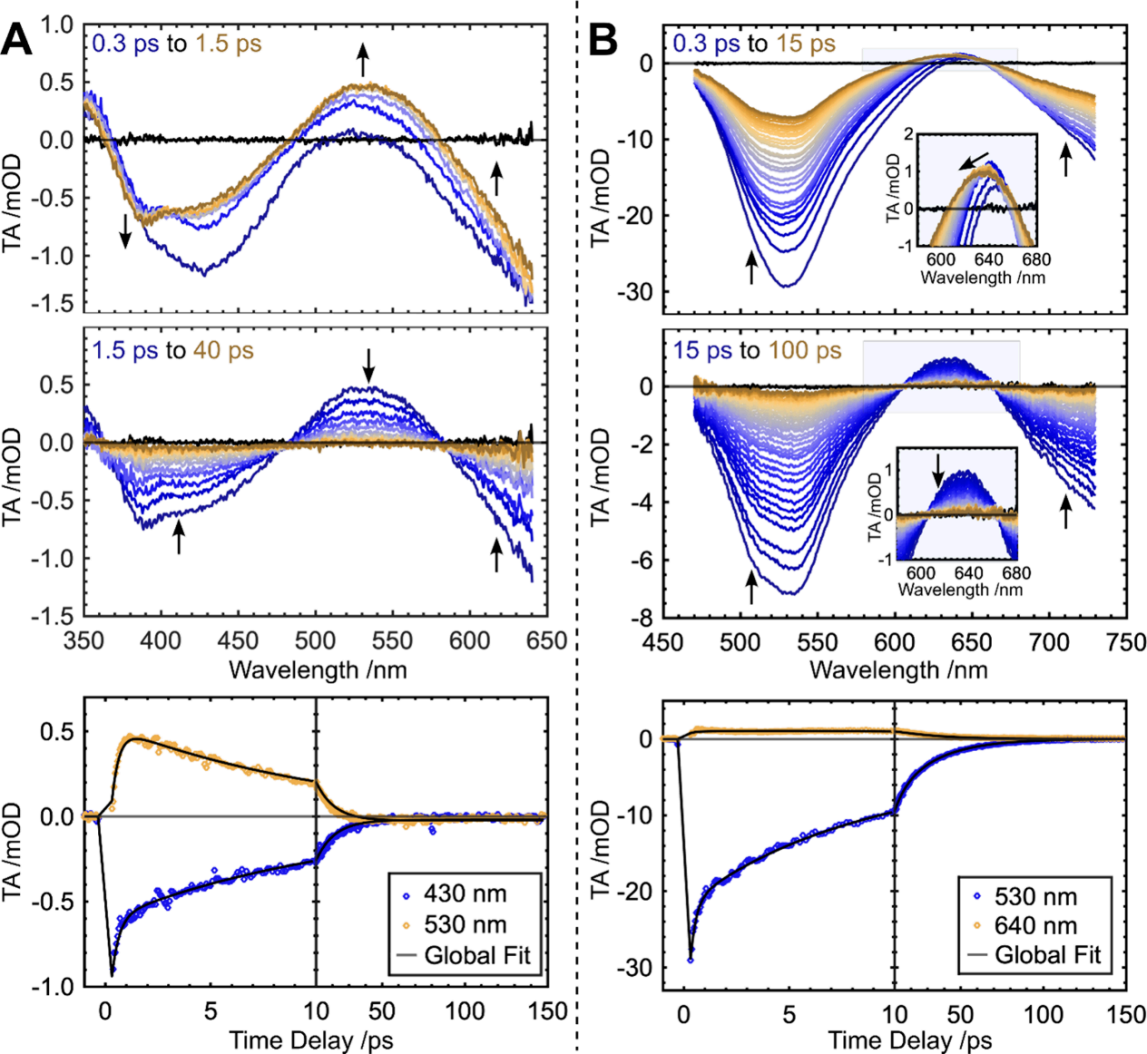
Cascaded difference spectra of (A) **1** and (B) **5**, across two time regions with TA prior to photoexcitation plotted in black. Kinetic traces at representative GSB and ESA wavelengths plotted in bottom panel alongside the fit to the data. Insets are an enlarged view of the shaded area showing the evolution of the ESA. All TA spectra, alongside their corresponding global fits, are presented in Figures S11–S32.

The ultrafast dynamics of the eight compounds fall into two groups (Table 1); analysis of **1** and **5** are presented as representative compounds. Difference spectra across two ranges of time-delays following photoexcitation of **1** (700 nm, 1 μJ/pulse) into its lowest energy MLCT are plotted in Figure 3A. Within the first picosecond, GSB and ESA peaks are observed at 390 and 540 nm, respectively, with the ESA red-shifting by ∼300 cm^–1^. Subsequently, a simultaneous decay of the whole difference spectrum occurs from 1.5 to 40 ps, as evidenced by isosbestic points at 479 and 583 nm. By 100 ps, the ground state of **1** has fully recovered. We note that there remains a small, flat, negative feature in the TA; this minor signal has no spectral features in common with the Ni(I) TA signal and is assigned to fine particulate scatter following the pump pulse (Supporting Information, Section S1.5). Using global multiexponential fitting to quantify the relaxation dynamics, the TA spectrum of **1** is found to be well-described by two exponentials with time constants of 0.3 and 10.9 ps, plus a small constant offset. Difference spectra for **5** following photoexcitation (800 nm, 1 μJ/pulse) (Figure 3B) exhibit a GSB ∼25-fold larger than **1**, despite the comparable concentrations and pump power. The magnitude of the GSB signal of **5** is contrasted by its smaller ESA at 640 nm (Figure 3B, inset), which reveals an initial growth and blue-shift (∼380 cm^–1^) in the first picosecond, followed by a significant broadening and loss of intensity. Fifteen picoseconds after the excitation pulse, isosbestic points appear at 605 and 662 nm, signifying the recovery of the whole difference spectrum to the ground state. Three exponentials are required to fit to the data: the shortest time constant (τ_1_ = 0.6 ps) corresponds to the initial growth of the spectrum, while the intermediate and longer time constants correspond to the blue-shift/spectral broadening (τ_2_ = 5.6 ps) and the relaxation of the difference spectrum to the ground state (τ_3_ = 24.2 ps), respectively.

**Table 1:** Lowest-Energy MLCT Absorption Positions and Relaxation Time Constants for All Compounds in THF

**Compound**	^**R**^**bpy/halide**	**MLCT λ**_**max**_(nm/cm^–1^)	**τ**_**1**_**(ps)**^a^	**τ**_**2**_**(ps)**^a^	**τ**_**3**_**(ps)**
**1**	*t*-Bu/Cl	660/15 150	0.3		10.9^c^
**1-Br**	*t*-Bu/Br	653/15 310	0.5		13.9
**1-I**	*t*-Bu/I	640/15 625	0.4	1.2	15.4
**2**	Me/Cl	660/15 150			12
**3**	H/Cl	673/14 860	0.4		10
**4**	Ph/Cl	1175^b^/8510	0.5	5.2	22.3
**5**	MeO_2_C/Cl	1178/8490	0.6	5.6	24.2^c^
**5′**	MeO_2_C/Cl	1178/8490	0.2	5.0	23.9
**5-Br**	MeO_2_C/Br	1167/8570	0.4	5.6	23.8

a In the case of the quickly relaxing compounds (**1**–**3**), we are only able to resolve one fast component (see Section 3 (Discussion) below).

b Identified by using both the UV–vis–NIR absorption peak and the ground state bleach feature in the TA spectrum (Figure S31).

c Lifetimes of 12.4 and 29.2 ps for **1** and **5** in toluene, respectively.

As discussed in the Supporting Information, Section S1.5, the difference in behavior between **1** and **5** is not the result of pumping different MLCT bands, solvent coordination, pump saturation, or multiphoton effects.

### 2.2 Isolation and Characterization of Ni(I)(^MeO_2_C^bpy)Cl, **5′**

Given the disparate excited-state lifetimes for **1** and **5**, we sought their isolation and independent study. Unfortunately, this was not possible for **1**, as it was prone to dimerization, consistent with previous works.^24,32^ Compound **5**, however, does not dimerize at room temperature. Thus, we approached the synthesis of **5** photochemically. The parent Ni(II)(^MeO_2_C^bpy)(*o*-tolyl)Cl complex is only sparingly soluble in diethyl ether; we reasoned the more polar, three-coordinate Ni(I)(^MeO_2_C^bpy)Cl complex would be even less so. Indeed, irradiation of the deep purple precursor rapidly afforded a blue precipitate, (**5′**), which was collected and analyzed.

**Figure 4:**
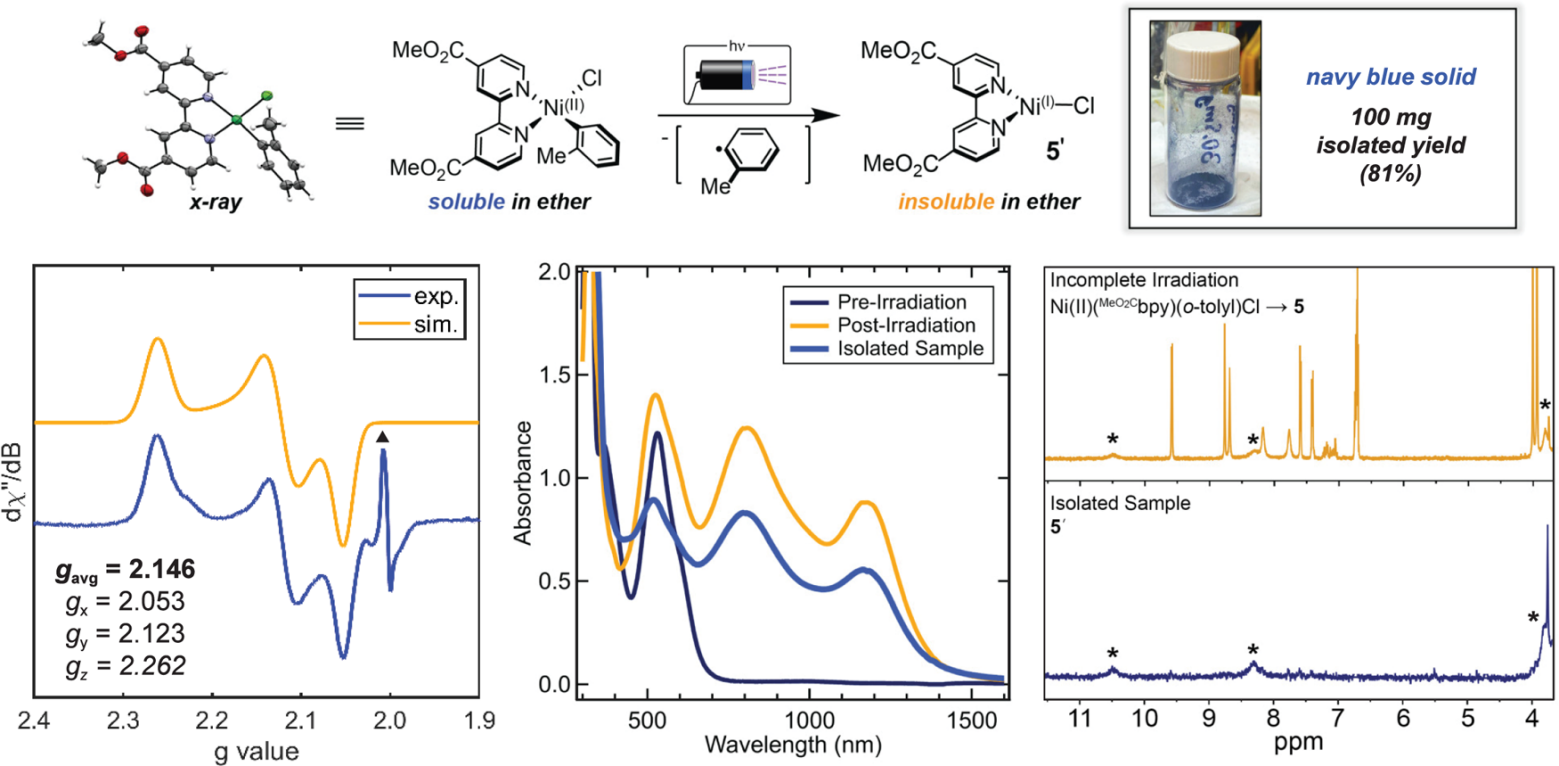
Photochemical generation and isolation of **5′**. Top: Synthetic route for the isolation of solid sample, **5′**. Bottom left: Powder X-band CW-EPR spectra of **5′** (*T* = 5 K; powder sample; frequency = 9.638 GHz; power = 2.2 mW; modulation amplitude = 8 G). Simulation parameters: *g*_*x*_ = 2.053, *g*_*y*_ = 2.123, *g*_*z*_ = 2.262, *g*(strain)_*x*_ = 0.025, *g*(strain)_*y*_ = 0.035, *g*(strain)_*z*_ = 0.033. The sharp signal denoted with the triangle at *g* = 2.003 likely corresponds to a trace amount of an organic radical impurity (present in a ∼1:10 000 ratio relative to **5′** by spin standard measurements with TEMPO). Bottom middle: UV–vis–NIR spectra of **5** and **5′** in THF. Bottom right: ^1^H NMR spectra (*d*_8_-THF) of a partially photolyzed sample of parent Ni(II)(^MeO_2_C^bpy)(*o*-tolyl)Cl complex to generate **5** (starred peaks) compared to the isolated complex **5′**.

While small and darkly colored crystals were grown by slow evaporation of a concentrated solution of **5′** in either 2-methyl THF or toluene, they were highly air sensitive and too small for XRD analysis. We instead turned to EPR. Solid-state EPR at 5 K showed a broadened rhombic signal (*g*_avg_ = 2.146, *g*_*x*_ = 2.053, *g*_*y*_ = 2.123, *g*_*z*_ = 2.262; Figure 4). The *g* values agree well with previously reported frozen glass solutions of Ni(I)–bpy complexes (*g*_avg_ = 2.12–2.24) and the predicted *g*_avg_ of the photogenerated species **5** (calculated *g*_avg_ = 2.187).^24,35,36^ Interestingly, IR spectra of solid samples exhibited a lowered carbonyl stretching frequency (ν_C=O_ = 1716 cm^–1^) in **5′** relative to its precursor Ni(II) complex (ν_C=O_ = 1730 cm^–1^),^15^ indicative of greater electron density in the bpy ligand π*-orbital via increased back-bonding from Ni(I).

Redissolving **5′** in THF gave an identical UV–vis–NIR spectrum to **5** (Figure 4). Evans Method in deuterated benzene gave μ_eff_ = 1.9 (n_e-_ = 1.2), and EPR spectra of frozen solutions were consistent with a Ni(I) species (Figures S1 and S2). Furthermore, comparison between the paramagnetic ^1^H NMR spectra of *in situ* photogenerated **5** and isolated **5′** showed excellent agreement (Figure 4). Notably, the NMR does not match the related tetrameric sample, [Ni(I)(^EtO_2_C^bpy)Cl]_4_, and we see no evidence of a redox equilibrium with Ni(0), as was seen previously for the tetramer (Figure S55).^19^ Addition of 2-bromobenzotrifluoride to a solution of **5′** gave rapid conversion to the oxidative addition product, Ni(II)(^MeO_2_C^bpy)(*o*-CF_3_Ph)Br, as confirmed by UV–vis–NIR and ^19^F NMR spectra of the independently synthesized Ni(II) complex (Figures S3 and S4). Finally, TA measurements on **5′** gave signals identical to **5** (Figures S28 and S29). Altogether, **5′** is most likely monomeric Ni(I)(^MeO_2_C^bpy)Cl.

## 3 Discussion

**Figure 5:**
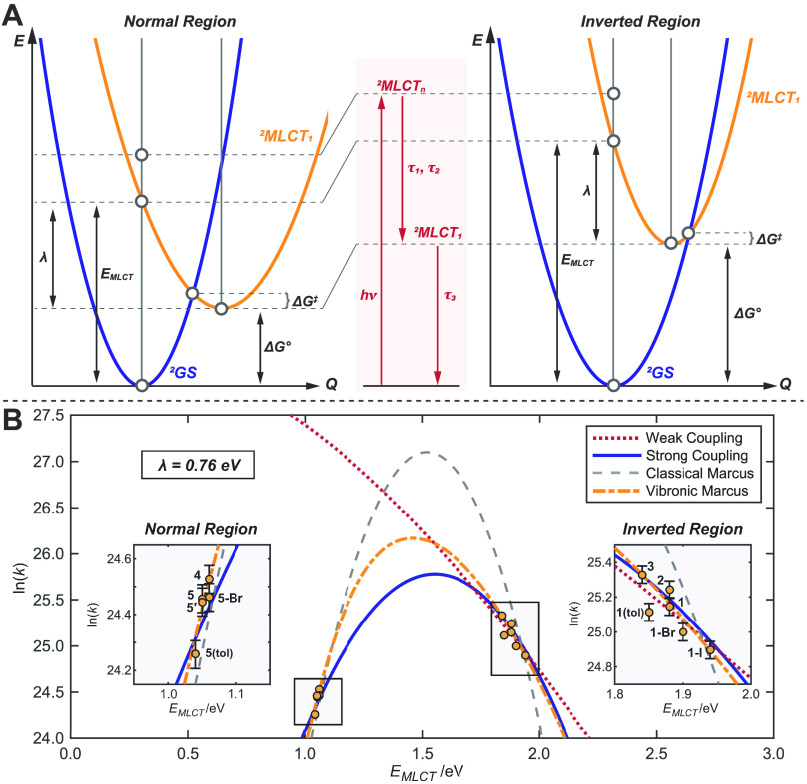
Modeling excited-state relaxation dynamics of Ni(I) compounds. (A) Potential energy surfaces and Jablonski diagram (in red) demonstrating the relaxation pathways within the strong coupling limit, which qualitatively correspond to the Marcus normal (left) and inverted (right) regimes along the nuclear coordinate, *Q*. (B) Plot of the experimentally determined rate constants for relaxation from the lowest energy MLCT back to the ground state as a function of the MLCT vertical transition energy (values taken from Table 1). The data reveal a clear parabolic shape and are fit to the four models discussed in the text, with vibronic Marcus giving the best fit. Error bars were extrapolated from the standard deviation of replicate measurements (see the Supporting Information, Section S1.1). Data were collected in THF; complexes **1** and **5** were also measured in toluene; 1 eV = 8065 cm^–1^.

Given their broad absorption cross sections and relevance for photoredox reactions, Ni(I)–bpy halide complexes are important targets for fundamental and applied photophysical investigations. Herein, we have studied a library of Ni(I)–bpy complexes, **1**–**5**, using ultrafast TA spectroscopy. Following excitation, relaxation proceeds through a rapid, multistep process accompanied by red- or blue-shifting of peaks and spectral broadening, characteristic of internal conversion through intermediate MLCT manifolds, vibrational cooling, and/or solvation effects.^14,38,39,41−43^ Components τ_1_ and τ_2_ are therefore assigned to relaxation into the lowest-energy MLCT manifold (Figure 5A). For the fastest-relaxing compounds (**1**–**3**), we are unable to separately resolve these components. Subsequently, ESA and GSB features recover on a longer time scale with clearly defined isosbestic points; the presence of significant ESA in the visible at all times suggests persistent reduction of the bpy ligand in an MLCT state.^14^ The kinetics for ground state reformation are independent of pump wavelength, further indicating the rate-limiting step involves nonradiative relaxation out of the lowest-energy MLCT (τ_3_ in Table 1 and Figure 5A). Overall charge transfer lifetimes span ∼10–30 ps, comparable to some of the longest reported Ni(II) MLCT lifetimes only obtained through the synthetic incorporation of significant steric constraints.^38,40^ The simple three-coordinate structures of these Ni(I) intermediates may lead to limited vibrational degrees of freedom to relax to the ground state, possibly representing an alternative approach to prolonging excited-state lifetimes. Interestingly, the values of τ_3_ for the eight complexes also fall into two clear classes (exemplified by **1** and **5**).

Nonradiative decay can be examined by the approach of Englman and Jortner,^44^ which relates the decay rate constant (*k* = τ_3_^–1^) and the energy gap between fully relaxed excited and ground states, Δ*G*°. This model considers two limiting cases of the coupling between molecular vibrations and the decay rate, namely weak or strong coupling.

The weak coupling limit applies to excited states with a small displacement from the ground state along the vibrational coordinate. This regime is found to approximately result in the energy-gap law through the interaction of the excited state with the highest vibrational mode(s) of the ground state; the decay rate constant is linearly governed by the energy gap between the relaxed ground and excited state, i.e., ln(*k*) ∝ – Δ*G*°. A full discussion of the weak coupling limit is given in the Supporting Information, Section S1.6.

In the strong coupling limit, the displacement between the ground- and excited-state potential energy surfaces is large, making their intersection thermally accessible (Figure 5A). This activation energy gives rise to a Gaussian dependence on the energy gap, as in Marcus theory for intermolecular electron transfer.^45−48^ For small energy gaps, an increase in Δ*G*° leads to a decrease in the kinetic barrier, Δ*G*^‡^, for back-electron transfer (e.g., the relaxation from the MLCT to the ground state). Accordingly, the decay rate constant increases. For large energy gaps, Δ*G*° and Δ*G*^‡^ scale together; a larger energy gap corresponds to a reduced decay rate constant, akin to the Marcus inverted region. These two regimes of strong coupling behavior can be described by eq 1,
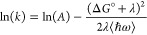
1where λ is the reorganization energy, ⟨*ℏ*ω⟩ the average vibrational energy, and *A* contains various transition-dependent parameters. Substituting ⟨*ℏ*ω⟩ = 2*k*_B_*T*, where *k*_*B*_ is the Boltzmann constant and *T* is the temperature, recovers the well-known Marcus equation. This difference in denominator follows from their derivations in the classical (Marcus) and quantum limits (strong coupling). Notably, a plot of ln(*k*) versus – \Δ*G*° results in a parabolic shape, which captures both the Marcus normal and inverted regimes.^49,50^

The value of the energy gap is often not easily accessible experimentally. However, λ is defined as the difference between the vertical transition energy (*E*_MLCT_) and Δ*G*°, enabling the substitution of the energy gap for the more experimentally accessible vertical transition energy, (44)
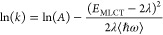
2

By considering the coupling of vibrations to intermolecular electron transfer, Jortner and Ulstrup developed an extended version of Marcus theory that has also seen success in modeling intramolecular electron transfer.^51−57^ Aside from vibrational considerations, the major difference of this model is the splitting of the reorganization energy into contributions from the solvent (λ_S_) and vibrational modes (λ_V_), or λ = λ_S_ + λ_V_. Making the approximation that one dominant vibronic mode (or a representative average mode) with energy *ℏ*ω couples to the transfer, this model simplifies to
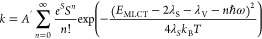
3where *A*′ is a constant defined in eq S6 and *S* = λ_V_/*ℏ*ω. We term this form of Jortner and Ulstrup’s model the vibronic Marcus model to distinguish it from the simpler, classically derived Marcus theory.

To evaluate which models are most representative of the relaxation kinetics of **1**–**5**, we plot ln(*k*) against *E*_MLCT_ (Figure 5B). Two clear clusters are visible; while **1**–**3** appear to follow the energy gap law, **4**–**5** exhibit the opposite behavior. The division of the excited-state dynamics into two clusters cannot be explained solely within the weak coupling regime, which predicts a single monotonic relationship over all complexes. This is highlighted by the fit to the weak coupling model (eq S3) shown in Figure 5B.

On the other hand, the strong coupling and Marcus models present a simple alternative to describe the two classes of relaxation kinetics. Using eq 2 with *A*, λ, and ⟨*ℏ*ω⟩ as variables, the strong coupling fit shown in Figure 5B is achieved. The model shows excellent agreement with the data; **1**–**3** and **4**–**5** lie in the inverted and normal regions, respectively. We find ⟨*ℏ*ω⟩ = 0.13(7) eV and λ = 0.77(3) eV. Perhaps coincidentally, ⟨*ℏ*ω⟩ is comparable to the value predicted by DFT (0.14 eV, Table S4). The reorganization energy is of comparable magnitude to similar metal-to-bpy charge transfer processes.^50,58,59^ Alternatively, fitting to the classical Marcus model also gives a reasonable, but marginally worse, fit to the data with λ varying no more than the error of the original fit.

The best fit to the data is achieved by the vibronic Marcus model (eq 3), giving λ = λ_S_ + λ_V_ = 0.76 eV. This can also be roughly approximated from Gaussian fits to the widths of MLCT transitions in the absorption spectrum, which results in λ ∼ 0.5 eV, in fair agreement (Figure S10).^53,60^ The dominant vibrational mode is fit as *ℏ*ω = 0.21 eV. Previous studies have maintained that intramolecular charge transfer typically couples to vibrations of the ligand backbone, such as bpy breathing modes in polypyridyl–Fe complexes.^52−54,61^ These modes are predicted by DFT at ∼0.2 eV for **1**–**5**, agreeing with the vibronic Marcus fit and these previous studies. The solvent and vibrational contributions to the reorganization energy are found by the fit to be *λ*_*S*_ = 0.54 eV and λ_V_ = 0.22 eV, respectively. These individual contributions are hard to measure experimentally, but λ_V_ can be estimated from TD-DFT (Figure S44). In **1** and **5**, λ_V_ is ∼0.18 and ∼0.33 eV, respectively (average value of 0.26 eV), which is close to the value obtained from the vibronic Marcus model. We note, however, that DFT is found to have limited applicability in these systems (Supporting Information, Section S2.5) and reported values of λ_V_ vary greatly.^52,54,55,61^ Nonetheless, the total reorganization energy found by the vibronic Marcus fit is consistent with both the classical Marcus and strong coupling models.

## 4 Conclusions

Herein, we have examined a library of Ni(I)–bpy halide complexes, including an isolable species accessible through a simple photochemical route. To the best of our knowledge, this research represents the first ultrafast TA spectroscopic characterization of any Ni(I) complex. As such, it is also the first ultrafast characterization of a Ni(I) intermediate in metallaphotoredox catalysis. Ni(I)–bpy halide ^2^MLCT lifetimes are <50 ps, prohibiting diffusion-controlled bimolecular chemistry. Varying the bpy substituents significantly influences the excited-state relaxation dynamics, with kinetics that span the Marcus normal and inverted regimes.

## Supporting Information Available

Experimental and computational methods, synthetic details, X-ray crystallography, UV–vis/photochemical data, transient absorption spectra, NMR/IR spectra, calculated properties, and Cartesian coordinates of optimized structures (PDF)
