# Supplementary material for: Ultrafast Photophysics of Ni(I)–Bipyridine Halide Complexes: Spanning the Marcus Normal and Inverted Regimes
Source: J Am Chem Soc. 2024 May 22;146(22):15506–14. doi: 10.1021/jacs.4c04091 (PMC11157544; doi:10.1021/jacs.4c04091)
Supplement: Supplementary file 1 — ja4c04091_si_001.pdf [file ja4c04091_si_001.pdf]

Supporting Information

Ultrafast Photophysics of Ni(I)–bipyridine Halide Complexes: Spanning the Marcus Normal and Inverted Regimes

Erica Sutcliffe†, David A. Cagan†, Ryan G. Hadt*

[a]Division of Chemistry and Chemical Engineering, Arthur Amos Noyes Laboratory of Chemical Physics, California Institute of Technology, Pasadena, California 91125, United States

†Authors contributed equally

*Corresponding Author: rghadt@caltech.edu

Table of Contents

S1. Experimental Section **3**

S1.1. General Considerations. 3

S1.2. Synthetic Details. 6

Preparation of Parent Ni(II)–bpy Aryl Halide Complexes. 6

Photochemical Preparation of Ni(I)–bpy Halide Complexes. 9

Isolation of a Ni(I)–bpy Halide Complex. 12

S1.3. X-ray Crystallography. 15

S1.4. Steady-State UV-vis-NIR Spectroscopy. 17

Steady-State Spectra 17

Fitting of Steady-State Spectra 19

S1.5 Time-Resolved Spectroscopy 21

Transient Absorption Spectra 21

Discussion of Anomalous Long-lived Feature 43

S1.6 Fits to Alternative Relaxation Models 46

S2. Computational Section. **51**

S2.1. General Computational Details. 51

S2.2. Sample ORCA Inputs. 52

S2.3. DFT Molecular Orbital Diagrams and Vibrational Energies 53

S2.4. TDDFT Spectra and Tabulated Transitions 63

S2.5 Limitations of DFT/TD-DFT 73

S3. NMR and IR Spectra. **74**

S4. Appendix. **81**

S5. References **85**

S1. Experimental Section.

S1.1. General Considerations.

All purchased compounds were used as received unless otherwise noted. Bis-(1,5-cyclooctadiene) nickel(0) was purchased from Strem Chemicals. Ligands N,N,N′,N′-tetramethyl ethylenediamine (TMEDA), 4,4′-di-*tert*-butyl-2,2′-bipyridine ($^{t\text{-Bu}}$bpy), 4,4′-dimethyl-2,2′-bipyridine (^{Me}bpy), 2,2′-bipyridine (bpy), 4,4'-diphenyl-2,2'-bipyridine (^{Ph}bpy), and dimethyl-2,2′-bipyridine-4,4′-dicarboxylate (^{MeO2C}bpy) were purchased from Sigma-Aldrich. Aryl halide compounds, 2-*chloro*-toluene, 2-*bromo*-toluene, 2-*iodo*-toluene, and 2-*bromo*-α,α,α-trifluorotoluene were also obtained from Sigma-Aldrich. Solids were dried under vacuum and brought into a nitrogen-atmosphere glove box; liquids (including aryl halides) were sparged (N_2) and degassed via freeze-pump-thaw techniques, brought into the glove box, and stored over 3 Å molecular sieves. All solvents were air-free and collected from a solvent purification system (SPS), then stored in the glove box over 3 Å molecular sieves in amber jars. Tetrahydrofuran (THF), 2-methyl tetrahydrofuran (2-MeTHF), and d_8-tetrahydrofuran (d_8-THF) were inhibitor-free. All deuterated solvents were purchased from Cambridge Isotope Laboratories, Inc. and also dried and stored over activated 3 Å molecular sieves in a nitrogen-filled glove box for at least three days before use. All synthesized compounds were made using air-free Schlenk techniques or made in the glove box. All synthesized complexes are considered air and moisture sensitive. Light sensitivity was also seen even in the solid state if left exposed for extended time.

UV-vis spectra of the complexes were obtained on a Varian Cary 500 spectrophotometer or a StellarNet Inc. Black Comet UV-vis spectrophotometer. Starna Cells 6-Q 2- or 10-mm path length cuvettes fitted with air-tight seals were used. Proton nuclear magnetic resonance (^{1}H NMR) and fluorine nuclear magnetic resonance (^{19}F NMR) spectra were recorded on a 400 MHz Varian Spectrometer with broadband auto-tune OneProbe. ^{19}F NMR were externally referenced to neat fluorobenzene (δ = -113.15 ppm). ^{13}C NMR spectra were collected on a Bruker AV-III HD 400 MHz spectrometer and were ^{1}H decoupled. Chemical shifts are reported in parts per million (δ in ppm, s: singlet, d: doublet, t: triplet, m: multiplet) and are referenced to residual solvent signal (THF-d_8 = 3.58 ppm). NMR samples were prepared in the glove box into Norell J-Young tubes. IR measurements were performed on a Bruker Alpha Platinum ATR spectrometer. Samples were analyzed with high resolution mass spectrometry (HRMS) by Field Desorption ionization using a JEOL AccuTOF GC-Alpha (JMS-T2000GC) mass spectrometer interfaced with an Agilent 8890 GC system. Electron paramagnetic resonance (EPR) spectroscopy was collected on a Bruker EMX X-band CW-EPR Spectrometer using either an Oxford ESR 900 liquid helium/nitrogen flow-through cryostat or a liquid nitrogen immersion dewar for experiments at a fixed temperature of 77 K. The recorded spectra were simulated in EasySpin for Matlab.[1] EPR samples were prepared in the glove box into Wilmad quartz low pressure/vacuum EPR tubes fitted with a with air-tight PTFE piston.

Ultrafast laser pulses used for the transient absorption (TA) measurements originate from a Coherent Astrella Ti:Sapphire amplifier system, which generates 5 mJ, 40 fs pulses centered on 800 nm at a 1 kHz repetition rate. These pulses were passed into an Ultrafast Systems Helios spectrometer system to carry out the transient absorption spectroscopy measurements. Pulses from the Astrella were attenuated and then delayed by the Helios' built-in 7 ns delay stage before undergoing supercontinuum generation to generate a white-light probe. Three different non-linear optical media were used to generate white light across the UV to the NIR as needed: CaF_2 (330 –

650 nm), Sapphire (470 – 750 nm), YAG (850 – 1600 nm). The majority of the beam was focused onto the sample and then subsequently into a fiber spectrometer, while the remaining portion of the beam bypassed the sample and was focused into a second fiber spectrometer to act as a reference.

Pump pulses were generated through various means using a stronger portion of the Astrella output than that of the probe. To generate 560, 700 and 1200 nm pump pulses, a Coherent OPerA optical parametric amplifier was used in various configurations. For pumping at 800nm, the fundamental output of the Astrella was used and for 400 nm the fundamental was frequency doubled in a β-barium borate (BBO) crystal (EKSMA Optics, 10 mm x 10 mm x 0.2 mm, $\theta = 29.2^{o}$, $\phi = 90^{o}$, P/P@400-800 nm) crystal. After wavelength manipulation, the pump was chopped, attenuated with a variable neutral density filter, focused onto the sample, and subsequently blocked.

TA samples were prepared in a nitrogen-filled glovebox using 2 mm quartz cuvettes fitted with air-tight PTFE piston seals (Schlenk cuvettes). Concentration varied between measurements but was chosen to maximize absorbance while minimizing the dimerization occurring at high concentrations. Thus, typical absorbances at the pumped wavelength were around 0.1 – 0.3 OD. Some compounds were especially prone to dimerization and the resultant precipitate caused significant scattered pump light in the results. This was minimized through the use of spectral filters (ThorLabs FELH0450, FESH0650, FESH0750) to block the pump wavelengths, but these also blocked wavelengths shorter than 400 nm so were only used when necessary. A magnetic stirrer was also used to stir the sample over the course of the measurement.

Data were acquired using the Helios control software and subsequently exported to a custom MATLAB script for processing. Each datapoint was averaged for 2 s (1000 total pump-probe cycles) and an exponentially-spaced time-array was used to capture the decay of the signal. Each scan was repeated five times. Background points collected before time-zero where no signal was present were subtracted from the remaining data to remove pump scatter and other unwanted effects. Following this, the chirp in the data was corrected by tracking the position of the center of the cross-phase modulation (XPM) feature across the first picosecond of the spectrum and resampling the data with the original time array to remove the chirp. The data were then globally fit through nonlinear least-squares to a series of exponentials convoluted with a gaussian instrument response function. Since the true pulse length at the sample is unknown, the width of the response function was set to 70 fs to best fit the data. In most cases, the XPM was many times larger than the actual signal and so greatly biased the fit. In this work our interest lies with the dynamics after the first picosecond so the signal between -0.3 and 0.3 ps (0.5 ps for toluene due to a wider XPM) was excluded from the fit. The fitting procedure yields several fitted time constants alongside the decay-associated spectrum (DAS) corresponding to each exponential decay. For some cases, the least-squared algorithm would not converge so coarse manual tuning of the parameters was necessary to provide a good fit. Further processing can convert the DAS into evolution- or species-associated spectra but the presence of significant vibrational cooling violates the assumption of bilinearity required by the global fitting procedure making subsequent postprocessing of the spectrum questionable. However, the time constants are still valid and the DAS can still be used to understand the origin of each component.

Errors on the fitted parameters can be estimated through the residuals and numerical Jacobian matrix outputted by the fitting algorithm. The errors on the time constants calculated this way ranged from around 0.1 to 1%. However, errors calculated this way notoriously underestimate the

true uncertainty and more accurate errors can only be garnered from more complex statistical methods such as bootstrapping.[2,3] Therefore, we adopt a compromise here. Across all compounds, **1** and **5** were studied most thoroughly so the standard deviation of τ_3 across many different samples and measurements was taken as the error and found to be 5 and 4%, respectively. Such a large number of individual measurements were not possible for all compounds, so instead several permutations of repeats of the noisiest, **4**, were fitted and their standard deviation found to also be around 5%. Therefore, we approximate the error on τ_3 to be 5% for all compounds. The shorter time constants typically correspond to a much smaller change in the signal and so have larger uncertainty. However, these components are of less relevance to the study as a whole and thus a detail consideration of their corresponding errors is considered beyond the scope of this work.

S1.2. Synthetic Details.

The parent four-coordinate complexes, Ni(II)($^{t\text{-Bu}}$bpy)(*o*-tolyl)Cl, Ni(II)($^{t\text{-Bu}}$bpy)(*o*-tolyl)Br, Ni(II)($^{t\text{-Bu}}$bpy)(*o*-tolyl)I, Ni(II)(bpy)(*o*-tolyl)Cl, and Ni(II)(^{MeO_2C}bpy)(*o*-tolyl)Cl, were synthesized according to previous reports.[4–6] Their spectroscopic properties were identical to those described prior. The precatalyst, Ni(II)TMEDA(*o*-tolyl)Br, and the parent complexes, Ni(II)(^{Me}bpy)(*o*-tolyl)Cl, Ni(II)(^{Ph}bpy)(*o*-tolyl)Cl, and Ni(II)(^{MeO_2C}bpy)(*o*-tolyl)Br were prepared as given below.

Preparation of Parent Ni(II)–bpy Aryl Halide Complexes.

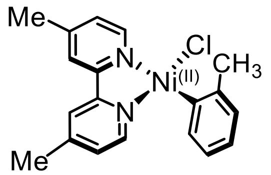

Ni(^{Me}bpy)(*o*-tolyl)Cl. In a nitrogen filled glove box, a 20 mL scintillation vial was charged with a Teflon coated stir bar, bis-(1,5-cyclooctadiene) nickel(0) (0.240 g, 0.870 mmol, 1.00 eq.), and 4,4′-dimethyl-2-2′-bipyridine (0.165 g, 0.896 mmol, 1.03 eq.). To this vial, 5.0 mL THF was added, and the mixture was stirred for 90 minutes affording a deep purple solution. Subsequently, 1.5 mL of 2-*chloro*-toluene (excess) was added dropwise, while stirring. An orange solid precipitated after 3.5 hours alongside a gray-black solid. Pentane (10 mL) were added to the mixture to complete precipitation. The crude solid mixture was then collected by filtration, washed with pentane and heptane (3x5 mL each), and the filtrate discarded. Into a second, clean filter flask, the solid mixture was rinsed with diethyl ether, affording a red/orange filtrate; the insoluble solids were discarded. To this filtrate, pentane was added to precipitate an orange solid. This solid was collected by filtration, washed again with pentane and heptane (3x5 mL each) then dried under vacuum (0.075 g, 23% yield). *Note:* The solid product is prone to decomposition over the course of days/weeks, even in the glove box, becoming an orange/brown solid. Sample should be stored at low temperature, if possible. Solutions decompose at room temperature over the course of several hours to days. Solutions should be used immediately to avoid insoluble decomposition products.

UV-vis (THF): λ_{MLCT} = 477 nm / 20,964 cm^{-1} (ε_{MLCT} = 4530 cm^{-1} M^{-1}). ^{1}H NMR (400 MHz, CD_2Cl_2): δ 8.97 (d, *J* = 6.0 Hz, 1H), 7.71 (d, *J* = 1.6 Hz, 1H), 7.67 (d, *J* = 1.5 Hz, 1H), 7.54 – 7.48 (m, 1H), 7.35 (dd, *J* = 5.2, 1.6 Hz, 1H), 7.10 (d, *J* = 5.7 Hz, 1H), 6.91 (dd, *J* = 5.5, 1.9 Hz, 1H), 6.83 – 6.74 (m, 3H), 3.04 (s, 3H), 2.49 (s, 3H), 2.37 (s, 3H). ^{13}C{^{1}H}NMR (100 MHz, CD_2Cl_2) δ 156.1, 151.3, 150.5, 149.2, 142.7, 135.8, 127.5, 127.2, 123.3, 123.2, 122.8, 121.8, 121.0, 25.2, 21.7, 21.5. FT-IR (ATR, cm^{-1}): 3036, 2977, 1615, 1556, 1478, 1445, 1418, 1024, 1018, 921, 846, 827, 733, 650, 556, 515. HRMS (FD-MS): calculated for $[C_{19}H_{19}N_2NiCl]^+$: 368.0590 found: 368.0584.

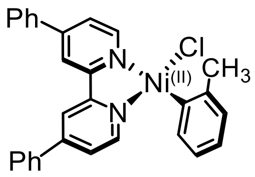

Ni(^{Ph}bpy)(*o*-tolyl)Cl. Synthetic procedure was adapted from a literature method.[5] In a nitrogen filled glove box, a 4 mL vial with air-tight septa cap was charged with a Teflon coated stir bar and Ni(TMEDA)(*o*-tolyl)Cl (0.024 g, 0.080 mmol, 1.00 eq.). To this vial, 4,4′-diphenyl-2,2′-bipyridine (0.026 g, 0.085 mmol, 1.06 eq.) was added along with 1.6 mL of benzene. The vial was capped and sealed with three turns of electrical tape, removed from the glove box, and stirred at 45 °C for 4 hours affording a dark red solution with precipitate. After allowing the vial to cool, it was brought back into the glove box where the red solid was collected by vacuum filtration, washed with benzene (2x2 mL), diethyl ether (2x2 mL), and pentane (4x2 mL), then was dried under vacuum (0.028 g, 73% yield). Spectroscopic properties were identical to those reported previously.

UV-vis (THF): λ_{MLCT} = 500 nm / 20,000 cm^{-1} (ε_{MLCT} = 5300). ^{1}H NMR (400 MHz, CD_2Cl_2): δ 9.25 (d, *J* = 5.7 Hz, 1H), 8.20 (s, 1H), 8.15 (s, 1H), 7.83 – 7.76 (m, 3H), 7.73 – 7.68 (m, 2H), 7.63 – 7.49 (m, 7H), 7.36 (d, *J* = 3.0 Hz, 2H), 6.89 – 6.78 (m, 3H), 3.09 (s, 3H). HRMS (FD-MS): calculated for $[C_{29}H_{23}N_2NiCl]^+$: 492.0903 found: 492.0923.

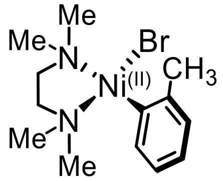

Ni(TMEDA)(*o*-tolyl)Br. In a nitrogen filled glove box, a 20 mL scintillation vial was charged with a Teflon coated stir bar and bis-(1,5-cyclooctadiene) nickel(0) (0.250 g, 0.909 mmol, 1 eq.). Via micro syringe, 0.175 mL (1.182 mmol, 1.3 eq.) of N,N,N′,N′-tetramethyl ethylenediamine was added along with 3.25 mL of 2-*bromo*-toluene (excess). A red/orange solid began precipitating in the vial. After 5 hours stirring at room temperature, hexanes was added (10 mL) to further precipitate the solid; the mixture was left overnight. The red/orange solid was collected by vacuum filtration where it was rinsed thoroughly with hexane and pentane, and dried (290 mg, 92% yield).

^{1}H NMR (400 MHz, CD_2Cl_2): δ 7.38 (dd, *J* = 7.3, 1.4 Hz, 1H), 6.70 – 6.62 (m, 2H), 6.61 – 6.53 (m, 1H), 3.41 (s, 4H), 2.79 – 2.31 (m, 12H), 2.17 (d, *J* = 10.4 Hz, 2H), 1.78 (s, 2H). ^{13}C{^{1}H}NMR (100 MHz, CD_2Cl_2) δ 144.8, 143.7, 136.2, 126.5, 122.5, 121.9, 61.4, 57.3, 50.1, 49.1, 47.7, 47.0, 26.7. FT-IR (ATR, cm^{-1}): 3037, 2971, 2893, 2840, 2784, 1558, 1456, 1277, 1123, 1047, 1018, 1010, 953, 806, 772, 749, 648, 605. HRMS (FD-MS): calculated for $[C_{13}H_{23}N_2NiBr]^+$: 344.0398 found: 344.0396.

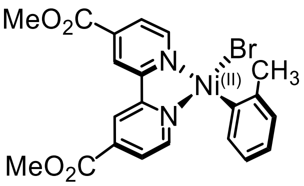

Ni(^{MeO_2C}bpy)(*o*-tolyl)Br. In a nitrogen filled glove box, a 100 mL Schlenk flask was charged with a Teflon coated stir bar and Ni(TMEDA)(*o*-tolyl)Br (0.250 g, 0.723 mmol, 1.00 eq.). To this vial, dimethyl-2,2′-bipyridine-4,4′-dicarboxylate (0.240 g, 0.881 mmol, 1.22 eq.) was added along with 24 mL of heptane and 4 mL toluene (7:1 heptane/toluene). Upon removal from the glove box, the flask was sonicated to promote solubilization of the reagents. The flask was attached to the nitrogen Schlenk line, covered in aluminum foil, and stirred at 60 °C for 24 hours affording a deep purple solution with purple precipitate. Orange starting material was still seen in the flask after inspection, so the temperature was then increased to 65 °C and the reaction continued for an additional 48 hours. After allowing the Schlenk flask to cool, it was brought back into the glove box. A purple solid had precipitated; it was collected by vacuum filtration, washed copiously with heptane, diethyl ether (3x2 mL), and excess pentane, then was dried under vacuum (0.255 g, 70% yield).

UV-vis (THF): λ_{MLCT} = 538 nm / 18,587 cm^{-1} (ε_{MLCT} = 5100 M^{-1} cm^{-1}). ^{1}H NMR (400 MHz, CD_2Cl_2): δ 9.73 (d, *J* = 5.0 Hz, 1H), 8.58 (d, *J* = 2.5 Hz, 1H), 8.51 (d, *J* = 1.4 Hz, 1H), 8.11 (dd, *J* = 5.7, 1.7 Hz, 1H), 7.71 (dd, *J* = 6.0, 1.8 Hz, 1H), 7.48 (dd, *J* = 6.9, 1.8 Hz, 1H), 7.43 (d, *J* = 6.1 Hz, 1H), 6.88 – 6.76 (m, 3H), 4.03 (s, 3H), 3.97 (s, 3H), 2.96 (s, 3H). ^{13}C{^{1}H}NMR (100 MHz, CD_2Cl_2) δ 164.1, 163.9, 155.8, 153.0, 152.2, 151.6, 147.4, 142.1, 139.7, 138.3, 135.6, 127.8, 126.3, 125.8, 123.5, 122.9, 120.8, 120.1, 25.1. FT-IR (ATR, cm^{-1}): 3028, 2950, 1725, 1556, 1433, 1398, 1322, 1250, 1232, 1121, 1012, 962, 884, 841, 840, 766, 737, 715, 649. HRMS (FD-MS): calculated for $[C_{21}H_{19}N_2O_4NiBr]^+$: 499.9882 found: 499.9899.

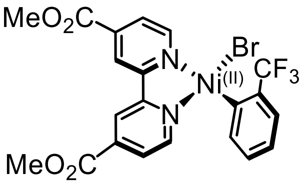

Ni(^{MeO_2C}bpy)(*o*-CF$_3$Ph)Br. In a nitrogen filled glove box, a 20 mL scintillation vial was charged with a Teflon coated stir bar, bis-(1,5-cyclooctadiene) nickel(0) (0.220 g, 0.800 mmol, 1.00 eq.), and dimethyl-2,2′-bipyridine-4,4′-dicarboxylate (0.225 g, 0.826 mmol, 1.03 eq.). To this vial, 5.0 mL THF was added, and the mixture was stirred for 90 minutes affording a deep purple solution. Subsequently, 2.0 mL of 2-bromobenzotrifluoride (excess) was added while stirring. The product solid precipitated after 4 hours. Hexane (10 mL) was added to the mixture to complete precipitation, and the mixture was placed in the glovebox freezer (-35 °C) for 60 hours. The solid was finally collected by filtration, washed thoroughly with hexane, diethyl ether (3x2 mL), and excess pentane, then dried under vacuum (0.405 g, 91% yield).

UV-vis (THF): λ_{MLCT} = 499 nm / 20,040 cm^{-1} (ε_{MLCT} = 4370 M^{-1} cm^{-1}). ^{1}H NMR (400 MHz, *d*$_8$-toluene): δ 9.74 (d, *J* = 5.7 Hz, 1H), 7.96 – 7.92 (m, 1H), 7.91 (s, 1H), 7.74 (s, 1H), 7.65 (s, 1H), 7.37 (d, *J* = 8.3 Hz, 2H), 7.22 (t, *J* = 5.2 Hz, 2H), 6.55 (d, *J* = 5.4 Hz, 1H), 3.40 (s, 3H), 3.36 (s, 3H). ^{19}F NMR (400 MHz, CD_2Cl_2) δ –58.6 ppm; (400 MHz, *d*$_8$-THF) δ –58.4 ppm. ^{13}C{^{1}H}NMR (100 MHz, CD_2Cl_2) δ 163.8, 155.8, 152.8, 152.1, 146.1, 137.9, 137.1, 128.3, 126.0, 125.7, 123.0,

120.8, 120.3, 120.2. Low signal to noise precluded the resolution of the $J_{C–F}$ coupling values for the trifluorotoluene peak. FT-IR (ATR, cm^{-1}): 3061, 2960, 1723, 1558, 1435, 1398, 1311, 1232, 1148, 1092, 1020, 967, 883, 842, 764, 735, 702, 675, 638. HRMS (FD-MS): calculated for $[C_{21}H_{16}N_2O_4F_3NiBr]^+$: 553.9599 found: 553.9594.

Photochemical Preparation of Ni(I)–bpy Halide Complexes.

Following our previous report,[6] the Ni(I)(^{R}bpy)X (R = *t*-Bu, Me, Ph, H, MeO_2C; X = Cl, Br, I) compounds studied herein were accessed directly from their parent Ni(II)–bpy aryl halide precursors by air- and moisture-free irradiation (370 nm or 390 nm).

Stock solutions of parent Ni(II)–bpy aryl halide complexes (0.5 – 1 mM) were prepared in a nitrogen-filled glove box and distributed into separate spectroscopic cuvettes (Starna Cells, 2- or 10-mm path length) fitted with air-tight PTFE piston seals (Schlenk cuvettes). Solutions were prepared fresh daily for analysis. Each cuvette was placed 5 cm away from either a Gen 2 Kessil PR160L 370 nm LED or Kessil PR160L 390 nm LED on highest setting. A cooling fan was used to maintain room temperature irradiation during the experiment. *Note:* Kessil LEDs may auto-shut off if left on for extended periods without the external fan due to overheating. Typical irradiation times, *t*, were ~60 minutes, but these varied with each complex and are listed below.

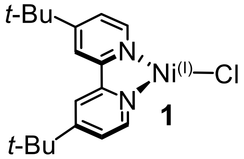

1

Ni(I)($^{t\text{-Bu}}$bpy)Cl, **1**. Air- and moisture-free irradiation of the parent Ni(II) compound, Ni(II)($^{t\text{-Bu}}$bpy)(*o*-tolyl)Cl, for 60 minutes using a Gen 2 Kessil PR160L 370 nm LED afforded the title compound. UV-vis (THF): λ_1 = 660 nm (15,152 cm^{-1}), λ_2 = 422 nm, (23,700 cm^{-1}). Spectroscopic properties were identical to those reported previously.

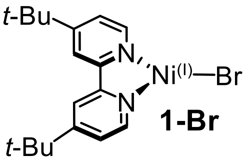

1-Br

Ni(I)($^{t\text{-Bu}}$bpy)Br, **1-Br**. Air- and moisture-free irradiation of the parent Ni(II) compound, Ni(II)($^{t\text{-Bu}}$bpy)(*o*-tolyl)Br, for 45 minutes using a Kessil PR160L 390 nm LED afforded the title compound. UV-vis (THF): λ_1 = 653 nm (15,314 cm^{-1}), λ_2 = 386 nm, (25,906 cm^{-1}). Spectroscopic properties were identical to those reported previously.

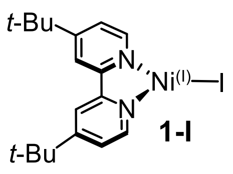

1-I

Ni(I)($^{t\text{-Bu}}$bpy)I, **1-I**. Air- and moisture-free irradiation of the parent Ni(II) compound, Ni(II)($^{t\text{-Bu}}$bpy)(*o*-tolyl)I, for 60 minutes using a Kessil PR160L 390 nm LED afforded the title compound. UV-vis (THF): λ_1 = 640 nm (15,625 cm^{-1}), λ_2 = 382 nm, (26,178 cm^{-1}). Spectroscopic properties were identical to those reported previously.

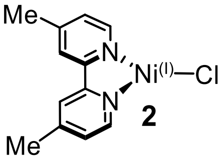

2

Ni(I)(^{Me}bpy)Cl, **2**. Air- and moisture-free irradiation of the parent Ni(II) compound, Ni(II)(^{Me}bpy)(*o*-tolyl)Cl, for 60 minutes using a Kessil PR160L 390 nm LED afforded the title compound. UV-vis (THF): λ_1 = 660 nm (15,152 cm^{-1}), λ_2 = 440 nm, (22,727 cm^{-1}).

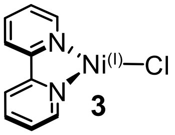

3

Ni(I)(^{H}bpy)Cl, **3**. Air- and moisture-free irradiation of the parent Ni(II) compound, Ni(II)(^{H}bpy)(*o*-tolyl)Cl, for 75 minutes using a Kessil PR160L 390 nm LED afforded the title compound. Precipitation can occur after extended irradiation; it can be filtered off in a glove box to yield a homogenous filtrate solution of the Ni(I) complex. UV-vis (THF): λ_1 = 673 nm (15,625 cm^{-1}), λ_2 = 431 nm, (23,200 cm^{-1}). Spectroscopic properties were identical to those reported previously.

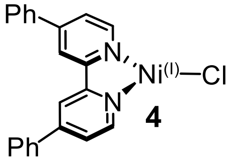

4

Ni(I)(^{Ph}bpy)Cl, **4**. Air- and moisture-free irradiation of the parent Ni(II) compound, Ni(II)(^{Ph}bpy)(*o*-tolyl)Cl, for 75 minutes using a Gen 2 Kessil PR160L 370 nm LED afforded the title compound. UV-vis (THF): λ_1 = 1175 nm (8,510 cm^{-1}), λ_2 = 915 nm, (10,929 cm^{-1}), λ_3 = 690 nm, (14,493 cm^{-1}), λ_4 = 485 nm, (20,619 cm^{-1}).

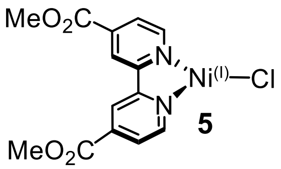

Ni(I)(^{MeO_2C}bpy)Cl, **5**. Air- and moisture-free irradiation of the parent Ni(II) compound, Ni(II)(^{MeO_2C}bpy)(*o*-tolyl)Cl, for 45 minutes using a Gen 2 Kessil PR160L 370 nm LED afforded the title compound. UV-vis (THF): λ_1 = 1178 nm (8,490 cm^{-1}), λ_2 = 805 nm (12,422 cm^{-1}), λ_3 = 523 nm (19,120 cm^{-1}). Spectroscopic properties were identical to those reported previously.

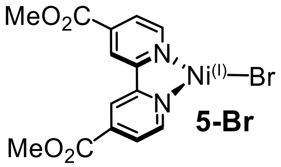

Ni(I)(^{MeO_2C}bpy)Br, **5-Br**. Air- and moisture-free irradiation of the parent Ni(II) compound, Ni(II)(^{MeO_2C}bpy)(*o*-tolyl)Br, for 45 minutes using a Gen 2 Kessil PR160L 370 nm LED afforded the title compound. UV-vis (THF): λ_1 = 1167 nm (8,570 cm^{-1}), λ_2 = 785 nm (12,739 cm^{-1}), λ_3 = 520 nm (19,231 cm^{-1}).

Table S1. Summary of the photochemical parameters used to generate the Ni(I)–bpy halide complexes from their Ni(II)–bpy aryl halide parents (Ar = *o*-tolyl) alongside their photochemical properties. MLCT peak positions and molar extinction coefficients are given for the lowest energy MLCT transition seen in the UV-vis-NIR data. Solvent = THF; *t* = irradiation time.

Parent Ni(II) Complex	Ni(II) MLCT (nm / cm^{-1})	Ni(II) ε_{MLCT} (M^{-1} cm^{-1})	LED (nm)	*t* (min)	Ni(I) Complex	Ni(I) MLCT (nm / cm^{-1})	Ni(I) ε_{MLCT} (M^{-1} cm^{-1})[a]
Ni$^{(II)}$($^{t\text{-Bu}}$bpy)(Ar)Cl	475 / 21 053	4970	370	60	**1**	660 / 15 150	2000
Ni$^{(II)}$($^{t\text{-Bu}}$bpy)(Ar)Br	479 / 20 877	3100	390	45	**1-Br**	653 / 15 310	2100
Ni$^{(II)}$($^{t\text{-Bu}}$bpy)(Ar)I	488 / 20 492	2200	390	60	**1-I**	640 / 15 625	1000
Ni$^{(II)}$(^{Me}bpy)(Ar)Cl	465 / 21 505	4540	390	60	**2**	660 / 15 150	1900
Ni$^{(II)}$(^{H}bpy)(Ar)Cl	483 / 20 704	4070	390	75	**3**	673 / 14 860	2100
Ni$^{(II)}$(^{Ph}bpy)(Ar)Cl	501 / 19 960	5400[b]	370	75	**4**	1175[c] / 8510	800
Ni$^{(II)}$(^{MeO_2C}bpy)(Ar)Cl	532 / 18 797	6100	370	45	**5**	1178 / 8490	5500
Ni$^{(II)}$(^{MeO_2C}bpy)(Ar)Br	538 / 18 587	5100	370	45	**5-Br**	1167 / 8570	2500

[a]Values obtained following complete photolysis of parent Ni(II) complexes and may be underestimated. [b]Reference value[5]. [c]Identified by using both the UV-vis-NIR absorption peak and the ground state bleach feature in the transient absorption spectrum (Figure S31).

Isolation of a Ni(I)–bpy Halide Complex.

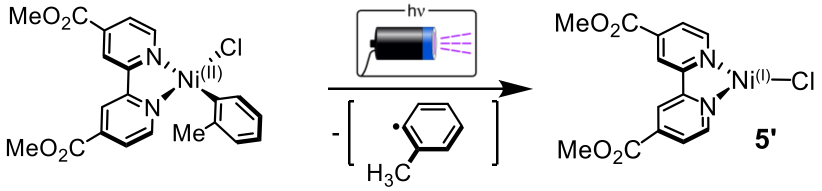

<u>Ni(I)(^{MeO_2C}bpy)Cl, **5′**.</u> In a nitrogen filled glove box Ni(II)(^{MeO_2C}bpy)(*o*-tolyl)Cl[6] (0.160 g, 0.350 mmol) was dissolved in 175.0 mL of diethyl ether. This purple solution was filtered to ensure homogeneity and transferred to a 350 mL Schlenk flask with a Teflon coated stir bar. The flask was sealed and removed from the glovebox. The flask was attached to a nitrogen Schlenk line and allowed to stir. Two Gen 2 Kessil PR160L 370 nm LEDs were pointed at the flask (one on either side); a fan was pointed at the entire setup to ensure room temperature irradiation. The LEDs were allowed to irradiate the solution on their maximum setting for 48 hours. During the course of the irradiation, a dark precipitate could be seen evolving from the solution. The flask was then returned to the glovebox, and the solid collected by vacuum filtration (fraction 1). The purple filtrate was transferred back into the Schlenk flask, removed from the glovebox, and reattached to the nitrogen line. Irradiation of the solution was continued in the same manner as before for an additional 24 hours, affording more precipitated solid. The flask was again returned to the glovebox, and the new solid collected by vacuum filtration (fraction 2); the filtrate was discarded. The collected navy blue solid was washed with excess diethyl ether, 2-methyl tetrahydrofuran (5x1 mL), hexane (3x2 mL), and pentane (3x2 mL), then dried under reduced pressure (fraction 1: 0.072 g, fraction 2: 0.029 g, combined fractions: 0.101 g, 81% yield).

Note: The title compound is highly air and moisture sensitive. It is insoluble in pentane, hexane, heptane, and ether. It is sparingly soluble in 2-MeTHF and is soluble in benzene, toluene, and THF. The compound reacts readily with dichloromethane (affording a red solution) and decomposes in acetonitrile (becoming a black solution). The compound readily reacts with aryl bromides.

Powder sample X-band CW-EPR (T = 5 K; frequency = 9.638 GHz; power = 2.2 mW; modulation amplitude = 8 G): g_{avg} = 2.146 (g_x = 2.053, g_y = 2.123, g_z = 2.262; $g(\text{strain})_x$ = 0.025, $g(\text{strain})_y$ = 0.035, $g(\text{strain})_z$ = 0.033. FT-IR solid sample (ATR, cm^{-1}): 3073, 2957, 1716, 1567, 1511, 1435, 1398, 1316, 1277, 1224, 1102, 1014, 992, 885, 836, 751, 726, 698, 542.

Frozen solution (toluene) X-band CW-EPR (T = 5 K; frequency = 9.639 GHz; power = 2.2 mW; modulation amplitude = 8 G): g_{iso} = 2.2011; g(strain) = 0.5. Frozen solution (THF) X-band CW-EPR (T = 5 K; frequency = 9.639 GHz; power = 2.2 mW; modulation amplitude = 8 G): $g_{1,iso}$ = 2.2011; g_1(strain) = 0.5; $g_{2,iso}$ = 2.1870; g_2(strain) = 0.0902. UV-vis (THF): λ_1 = 1178 nm (8,490 cm^{-1}), λ_2 = 805 nm (12,422 cm^{-1}), λ_3 = 523 nm (19,120 cm^{-1}). UV-vis (Benzene): λ_2 = 1181 nm (8,490 cm^{-1}), λ_2 = 820 nm (12,422 cm^{-1}), λ_3 = 526 nm (19,120 cm^{-1}). Paramagnetic ^{1}H NMR (400 MHz, d_8THF): δ 10.51 (br s, 1H), 8.31 (br s, 2H), 3.81 (br s, 3H). Effective magnetic moment (Evans method, 298 K, C_6D_6): 1.9 μB.

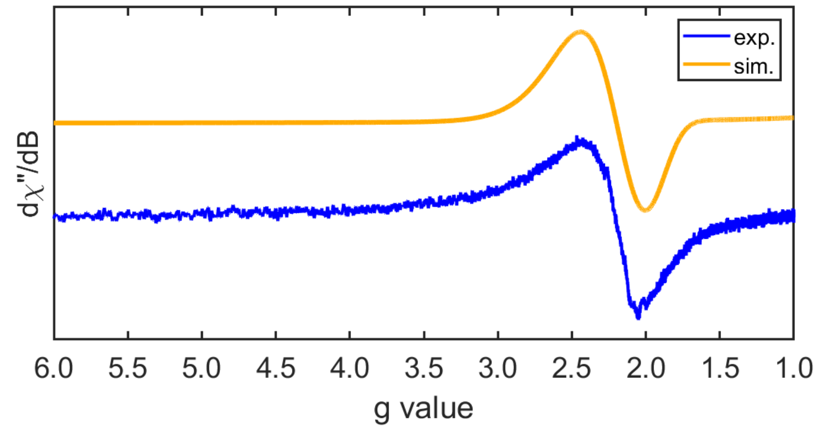

Figure S1. Frozen solution (toluene) X-band CW-EPR spectrum and fit (blue and orange lines, respectively) of **5′** (T = 5 K; frequency = 9.639 GHz; power = 2.2 mW; modulation amplitude = 8 G). Anisotropy in the signal could not be resolved due to line broadening, likely arriving from suspended particles of the complex which precipitated upon freezing or were not fully solvated. Simulation values: g_{iso} = 2.201; g(strain) = 0.5.

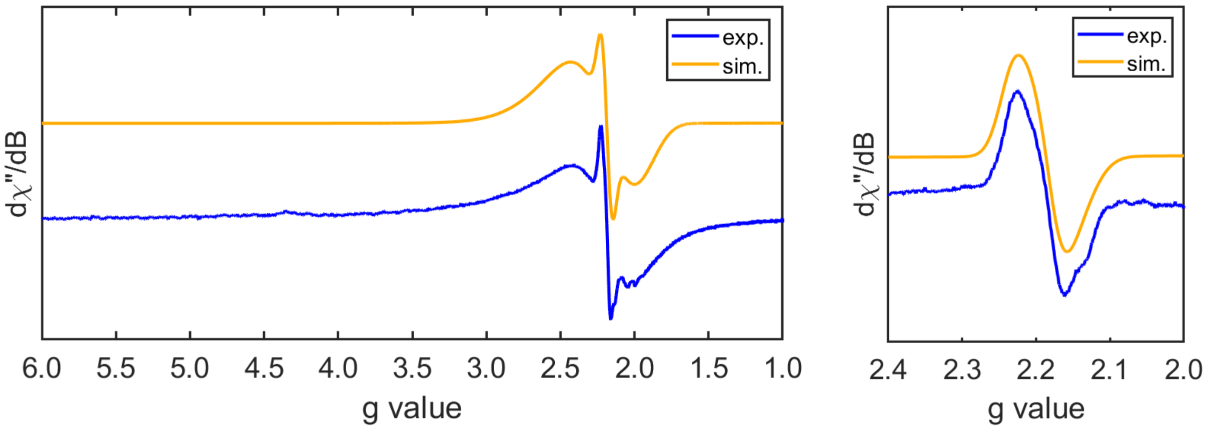

Figure S2. Frozen solution (THF) X-band CW-EPR spectrum and fit (blue and orange lines, respectively) of **5′** (T = 5 K; frequency = 9.639 GHz; power = 2.2 mW; modulation amplitude = 8 G). (*Left*) Full spectrum plotted showing two species; anisotropy in the signal could not be resolved due to line broadening, likely arising from suspended particles of the complex which precipitated upon freezing or were not fully solvated. The second feature in the spectrum (labeled as g_2) is attributed to a small fraction of the species coordinating to THF, which may occur during freezing of the sample. Dual spin simulation values: $g_{1,iso}$ = 2.201; g_1(strain) = 0.5; $g_{2,iso}$ = 2.187; g_2(strain) = 0.090. (*Right*) Spectrum after subtraction of the broad signal corresponding to g_1. Simulation values: g_z = 2.230, g_x = 2.180, g_y = 2.146, g_{avg} = 2.185; g_z(strain) = 0.043, g_x(strain) = 0.043, g_y(strain) = 0.065.

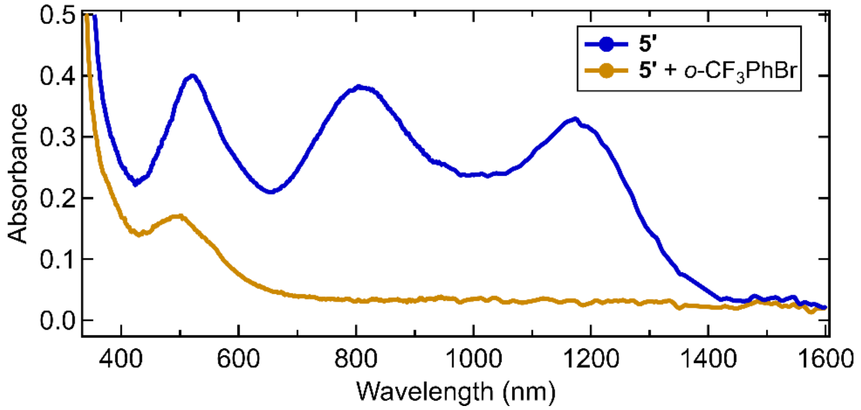

Figure S3. UV-vis-NIR spectra of **5′** pre- and post-addition (blue and orange lines, respectively) of 100 µL of 2-bromobenzotrifluoride.

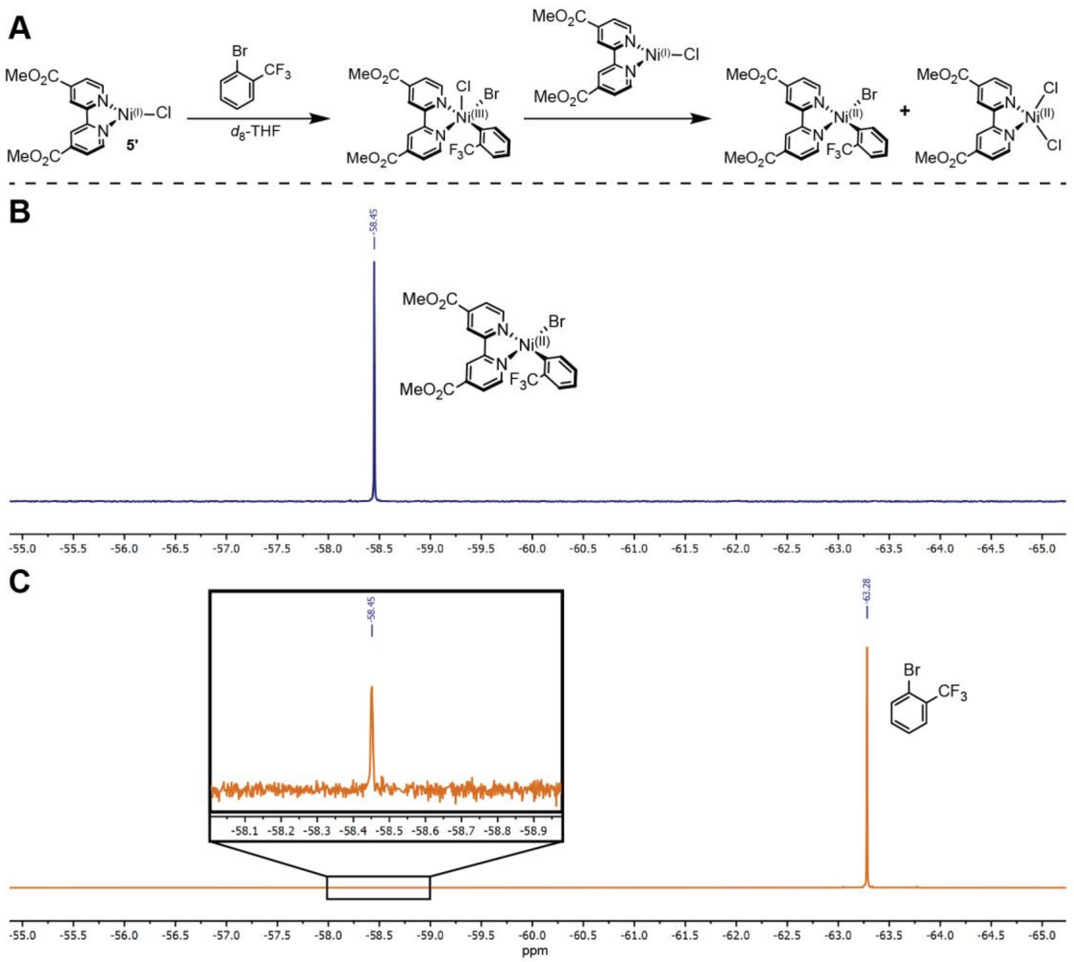

Figure S4. Aryl bromide reactivity analysis of **5′** by ^{19}F NMR (d_8-THF). (A) Reaction scheme for the oxidative addition of the aryl bromide by Ni(I), forming Ni(II)(^{MeO_2C}bpy)(o-CF_3Ph)Br and paramagnetic Ni(II)(^{MeO_2C}bpy)Cl_2.[6,7] (B) ^{19}F NMR spectrum of independently synthesized Ni(^{MeO_2C}bpy)(o-CF_3Ph)Br. (C) ^{19}F NMR spectrum of **5′** post-addition of 100 µL of 2-bromobenzotrifluoride.

S1.3. X-ray Crystallography.

Collection and Refinement Details for Ni(II)(^{MeO_2C}bpy)(*o*-tolyl)Cl.

Ni(II)(^{MeO_2C}bpy)(*o*-tolyl)Cl was crystallized by slow evaporation in diethyl ether. Low-temperature diffraction data (ϕ-and ω-scans) were collected on a Bruker AXS D8 VENTURE KAPPA diffractometer coupled to a PHOTON II CPAD detector with Mo K_α radiation (λ = 0.71073 Å) from an IμS micro-source for the structure of compound V23337. The structure was solved by direct methods using SHELXS[8] and refined against F^2 on all data by full-matrix least squares with SHELXL-2019[9] using established refinement techniques.[10] All non-hydrogen atoms were refined anisotropically. All hydrogen atoms were included into the model at geometrically calculated positions and refined using a riding model. The isotropic displacement parameters of all hydrogen atoms were fixed to 1.2 times the U value of the atoms they are linked to (1.5 times for methyl groups). All disordered atoms were refined with the help of similarity restraints on the 1,2- and 1,3-distances and displacement parameters as well as enhanced rigid bond restraints for anisotropic displacement parameters. Ni(II)(^{MeO_2C}bpy)(*o*-tolyl)Cl crystallizes in the monoclinic space group $P2_1/n$ with one molecule in the asymmetric unit. The *o*-tolyl group was modeled as a two-component disorder. These data are provided free of charge from The Cambridge Crystallographic Data Centre by The Cambridge Crystallographic Data Centre.

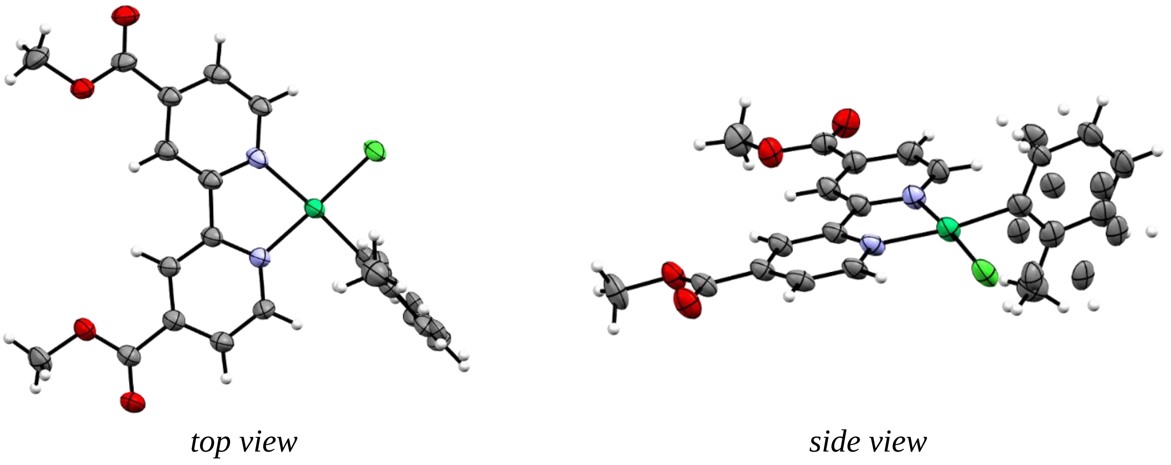

Figure S5. Top down and side views of the refined crystal structure of Ni(II)(^{MeO_2C}bpy)(*o*-tolyl)Cl. The *o*-tolyl group was modeled as a two-component disorder, with one conformer pointing up and the other pointing down.

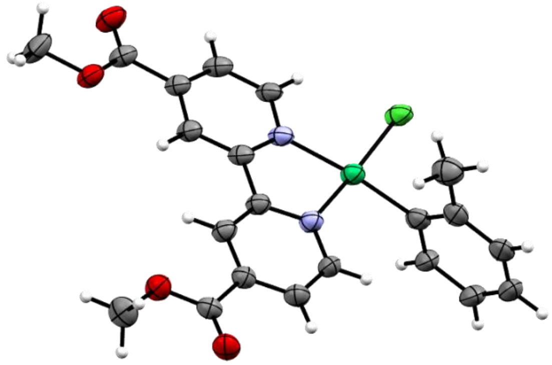

Figure S6. The refined crystal structure of Ni(II)(^{MeO_2C}bpy)(*o*-tolyl)Cl showing only one aryl ligand conformer for clarity.

**Compound**	**Ni(II)(^{MeO_2C}bpy)(*o*-tolyl)Cl**	
Empirical formula	$C_{21}H_{19}ClN_2NiO_4$	
Formula weight	457.54 g mol^{-1}	
Temperature	100(2) K	
Wavelength	1.54178 Å	
Crystal system	Monoclinic	
Space group	$P2_1/n$	
Unit cell dimensions	a = 7.1614(11) Å	a= 90°
	b = 22.156(2) Å	b= 102.715(10)°
	c = 12.972(3) Å	g = 90°
Volume	2007.8(6) Å^3	
Z	4	
Density (calculated)	1.514 Mg/m^3	
Absorption coefficient	2.881 mm^{-1}	
F(000)	944	
Crystal size	0.150 x 0.100 x 0.050 mm^3	
Theta range for data collection	3.990 to 74.733°.	
Index ranges	$-8 \leq h \leq 8$, $-27 \leq k \leq 27$, $-16 \leq l \leq 12$	
Reflections collected	23344	
Independent reflections	4086 [R(int) = 0.1627]	
Completeness to theta = 67.679°	99.9 %	
Absorption correction	Semi-empirical from equivalents	
Max. and min. transmission	0.7538 and 0.5432	
Refinement method	Full-matrix least-squares on F^2	
Data / restraints / parameters	4086 / 384 / 330	
Goodness-of-fit on F^2	1.073	
Final R indices [I>2sigma(I)]	R1 = 0.0699, wR2 = 0.1544	
R indices (all data)	R1 = 0.1140, wR2 = 0.1754	
Extinction coefficient	n/a	
Largest diff. peak and hole	0.674 and –0.450 e.Å^{-3}	

S1.4. Steady-State UV-vis-NIR Spectroscopy.

Steady-State Spectra

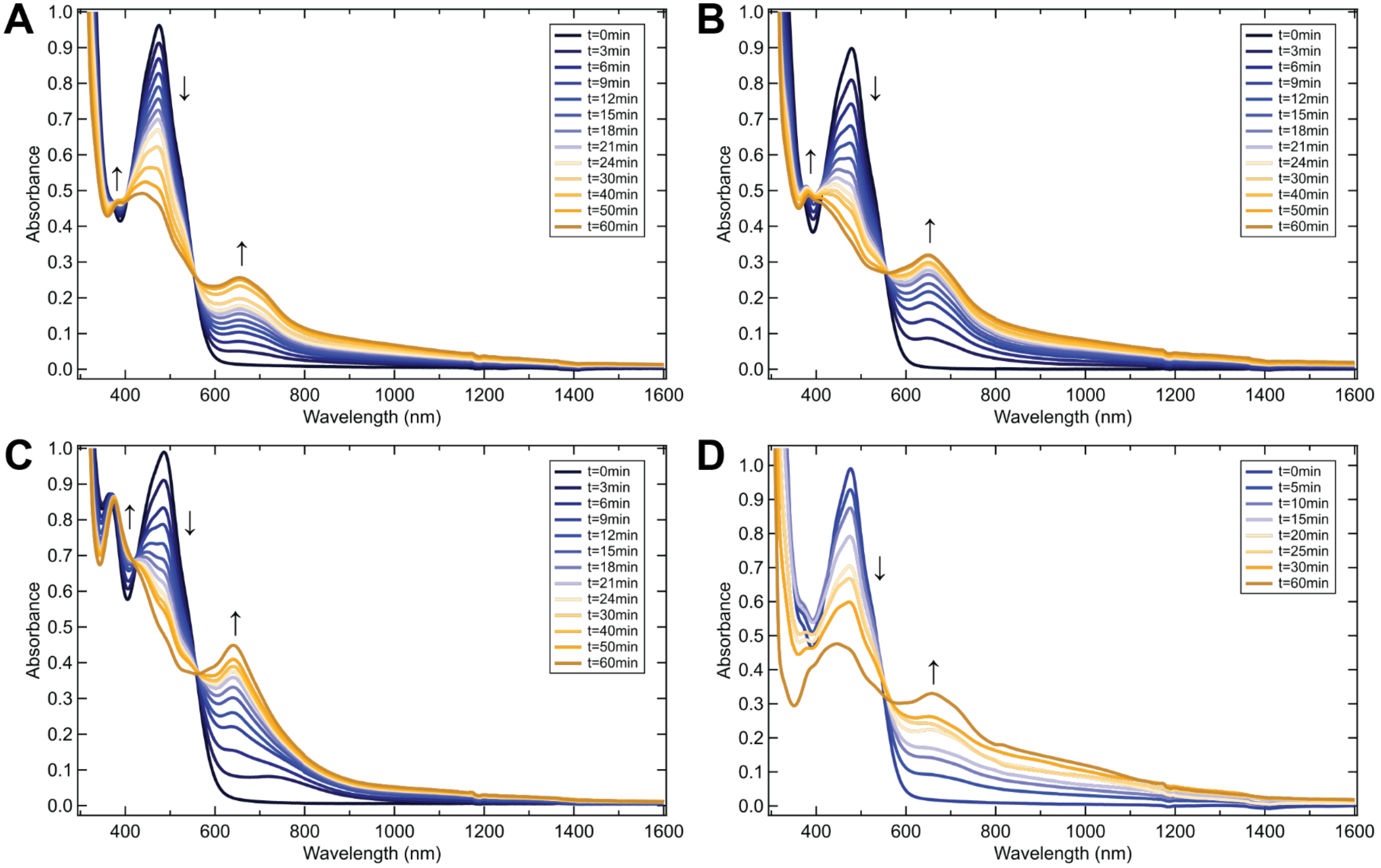

Figure S7. Time course UV-vis-NIR spectra in THF of the parent Ni(II)–bpy aryl halide compounds (blue line) being photogenerated to the Ni(I)–bpy halide complexes (orange line) examined in this work. (A) Photochemical conversion of Ni(II)($^{t\text{-Bu}}$bpy)(*o*-tolyl)Cl to Ni(I)($^{t\text{-Bu}}$bpy)Cl, **1**. (B) Photochemical conversion of Ni(II)($^{t\text{-Bu}}$bpy)(*o*-tolyl)Br to Ni(I)($^{t\text{-Bu}}$bpy)Br, **1-Br**. (C) Photochemical conversion of Ni(II)($^{t\text{-Bu}}$bpy)(*o*-tolyl)I to Ni(I)($^{t\text{-Bu}}$bpy)I, **1-I**. (D) Photochemical conversion of Ni(II)(^{Me}bpy)(*o*-tolyl)Cl to Ni(I)(^{Me}bpy)Cl, **2**.

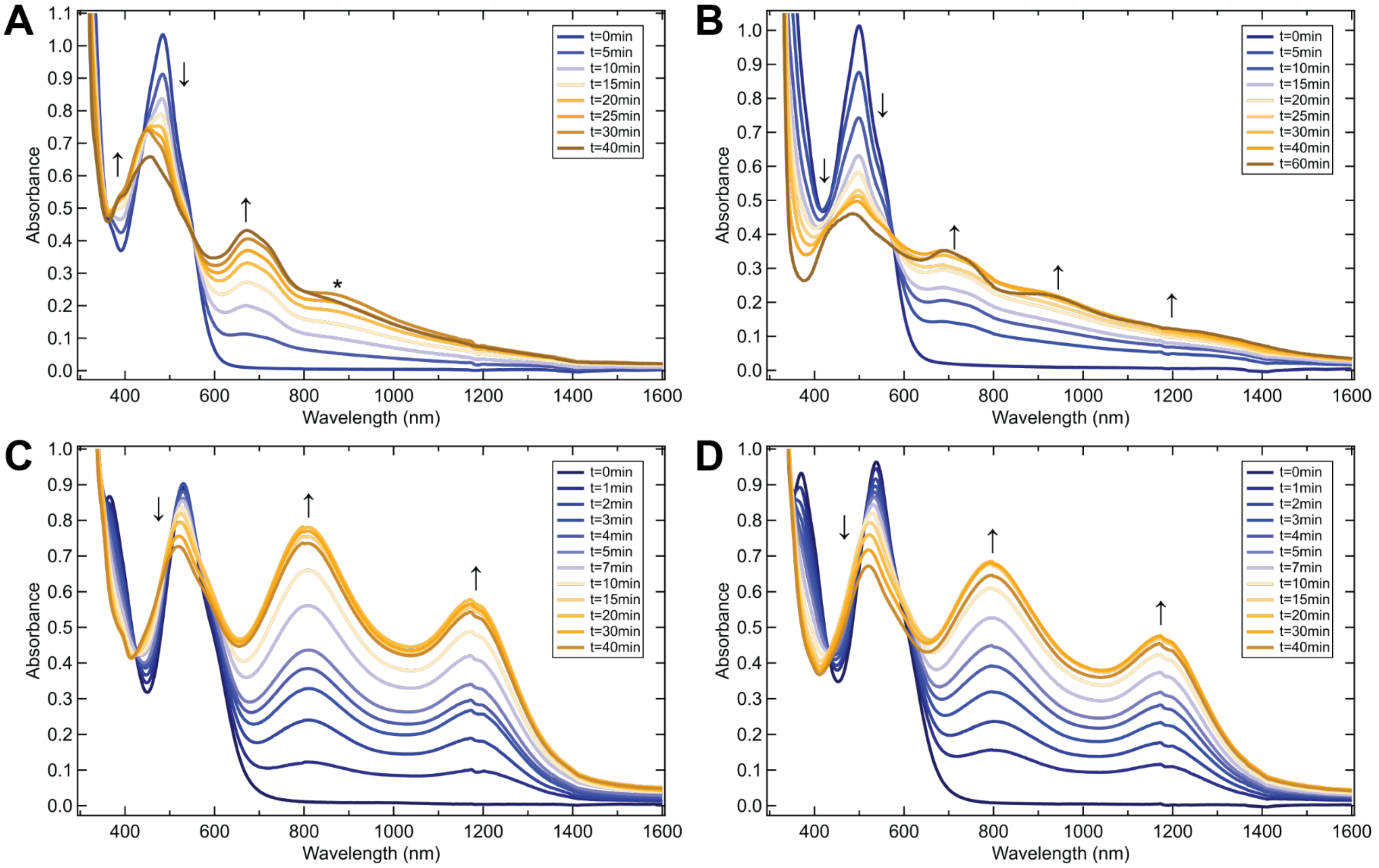

Figure S8. Time course UV-vis-NIR spectra in THF of the parent Ni(II)–bpy aryl halide compounds (blue line) being photogenerated to the Ni(I)–bpy halide complexes (orange line) examined in this work. (A) Photochemical conversion of Ni(II)(^{H}bpy)(*o*-tolyl)Cl to Ni(I)(^{H}bpy)Cl, **3**. Starred peak corresponds to an aggregation between Ni(II) and Ni(I) which is seen at low Ni(II) conversion.[6] (B) Photochemical conversion of Ni(II)(^{Ph}bpy)(*o*-tolyl)Cl to Ni(I)(^{Ph}bpy)Cl, **4**. (C) Photochemical conversion of Ni(II)(^{MeO_2C}bpy)(*o*-tolyl)Cl to Ni(I)(^{MeO_2C}bpy)Cl, **5**. (D) Photochemical conversion of Ni(II)(^{MeO_2C}bpy)(*o*-tolyl)Br to Ni(I)(^{MeO_2C}bpy)Br, **5-Br**.

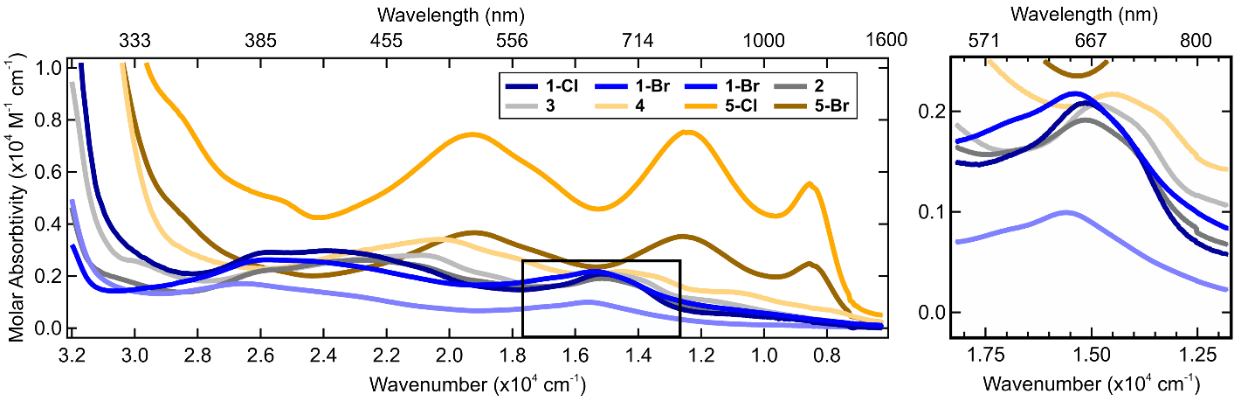

Figure S9. UV-vis-NIR absorption spectra of the photochemically generated Ni(I)–bpy halides in THF. Boxed section is expanded on the right. Analogous figure with wavelength axis is given as Figure 2.

The electronic structure of **1-5** and related compounds have been assigned by electron paramagnetic resonance (EPR) and quantum chemical calculations as having a $[d(\mathrm{xy})]^2$, $[d(\mathrm{yz})]^2$, $[d(\mathrm{xz})]^2$, $[d(\mathrm{z}^2)]^2$, $[d(\mathrm{x}^2–\mathrm{y}^2)]^1$ ground state.[11–14] The four β-β ligand field transitions are predicted by time-dependent density functional theory (TDDFT) to require only ~0.5–1.1 eV (~4050–8900 cm^{-1}) of photonic energy, placing them all in the IR to NIR region (~2500-1100 nm, see Supporting Information Section 2.4). Given the formal electric dipole forbidden nature of these transitions, their intensities would be much lower than those seen here. Contrastingly, the bpy π* orbitals lie energetically between the metal-based HOMO and LUMO (β-3$d(\mathrm{x}^2–\mathrm{y}^2)$), allowing for numerous spin- and orbitally-allowed *d-π** transitions across the full wavelength range shown. From these observations, the absorption bands can be assigned as Ni(I)-to-bpy MLCTs, consistent with our previous work.[11]

Fitting of Steady-State Spectra

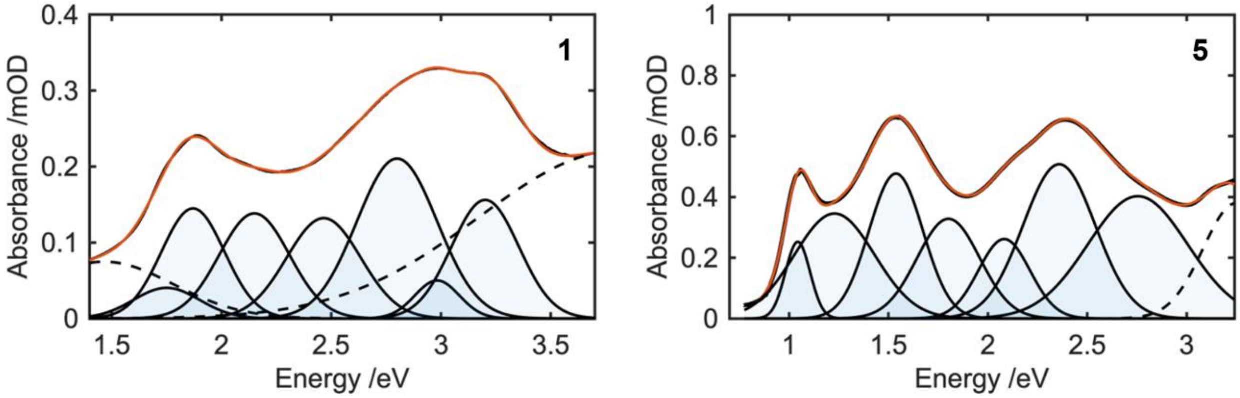

Figure S10. Fits to absorption spectra (red) alongside the raw spectrum (solid black) for compound **1** (left) and **5** (right) and the components to the fit as individual Gaussians underneath. The data were fit only in the wavelength region shown; components required to satisfy the boundaries are shown as dotted lines and not included in the fit.

We focus here on compounds **1** and **5** as representative examples. Given the number of transitions expected in these compounds and the inaccuracy of the TD-DFT, we cannot fit the data to a series

of vibronic progressions. This would provide the best comparison to the values obtained from equation 3, but the number of unknowns would severely limit the reliability of the fit. Therefore, we instead approximate the lineshape of each transition as a simple Gaussian and the absorption spectrum can be fitted to estimate the contributions of each transition to the absorption spectrum, $A(E)$. Mathematically, we fit to

$$A(E) = \sum_{i=1}^{N} a_i \exp\left(-\frac{(E - b_i)^2}{2c_i^2}\right), \tag{S1}$$

where E is photon energy, and a_i, b_i and c_i are fitting parameters which describe the amplitude, center, and width of each transition. Excellent fits to the data with only minor deviations are achieved using $N = 9$ for **1** and $N = 8$ for **5** with all parameters shown in Table S2; these are plotted in Figure S10. The dashed Gaussians are used to account for the boundaries of the fit and are not included the subsequent analysis. The total reorganization energy, λ, can be approximated from the peak widths as

$$\lambda \sim \frac{c^2}{2k_BT} \tag{S2}$$

Averaging over λ and taking the error on the mean gives $\lambda = 0.47(5)$ eV and $\lambda = 0.6(1)$ eV for **1** and **5**, respectively. Given the number of fitting parameters used, the overlapping nature of the peaks, and the assumed lineshape as a perfect Gaussian (no vibronic considerations) this fit is unlikely to be unique and constitutes an underestimate of the true λ. Despite this, all fitted peaks have similar widths suggesting that this is a reasonable approximation.

Table S2. Parameters for the fits shown in Figure S10 for complexes **1** and **5**. Components not included in the average are underneath the dotted line.

**1**			**5**		
a **(OD)**	***b*** **(eV)**	***c*** **(eV)**	***a*** **(OD)**	***b*** **(eV)**	***c*** **(eV)**
0.040	1.75	0.15	0.25	1.04	0.06
0.14	1.87	0.14	0.35	1.23	0.20
0.14	2.15	0.16	0.48	1.53	0.14
0.13	2.47	0.17	0.33	1.80	0.16
0.050	2.98	0.10	0.26	2.08	0.13
0.16	3.2	0.15	0.51	2.35	0.18
0.21	2.80	0.18	0.40	2.75	0.25
0.075	*1.46*	*0.31*	*0.38*	*3.25*	*0.17*
0.22	*3.81*	*0.64*	-	-	-

S1.5 Time-Resolved Spectroscopy

Transient Absorption Spectra

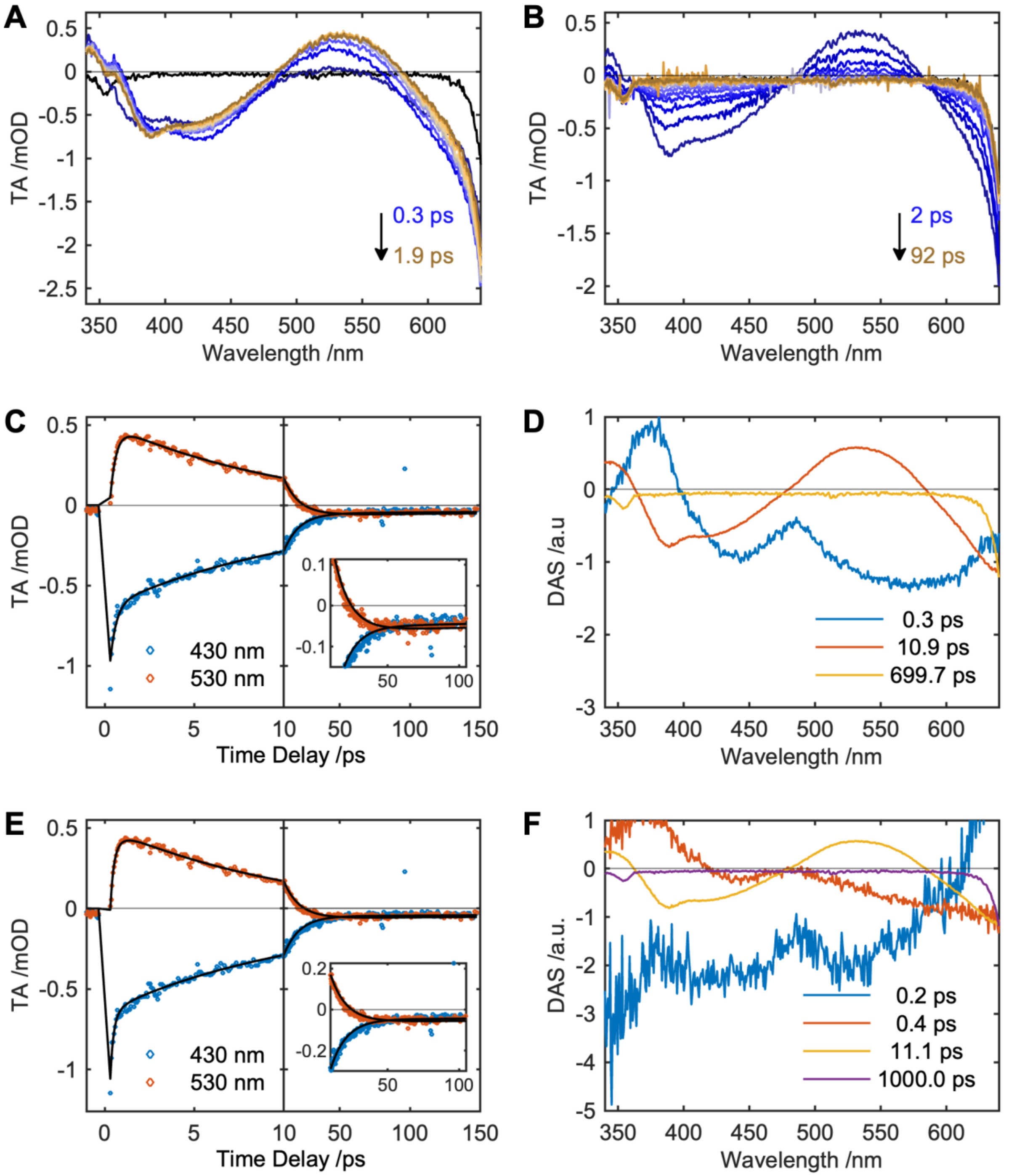

Figure S11. TA data for **1** in THF following 700 nm, 1 μJ photoexcitation. (A,B) Cascaded difference spectra across the two time regions indicated. (C,D) and (E,F) show results of three and four component global fits, respectively. (C,E) Kinetic traces at indicative wavelengths across two time regions. Inset is an enlarged view of the traces at long times. (D,F) DAS corresponding to the time constants indicated in the legend.

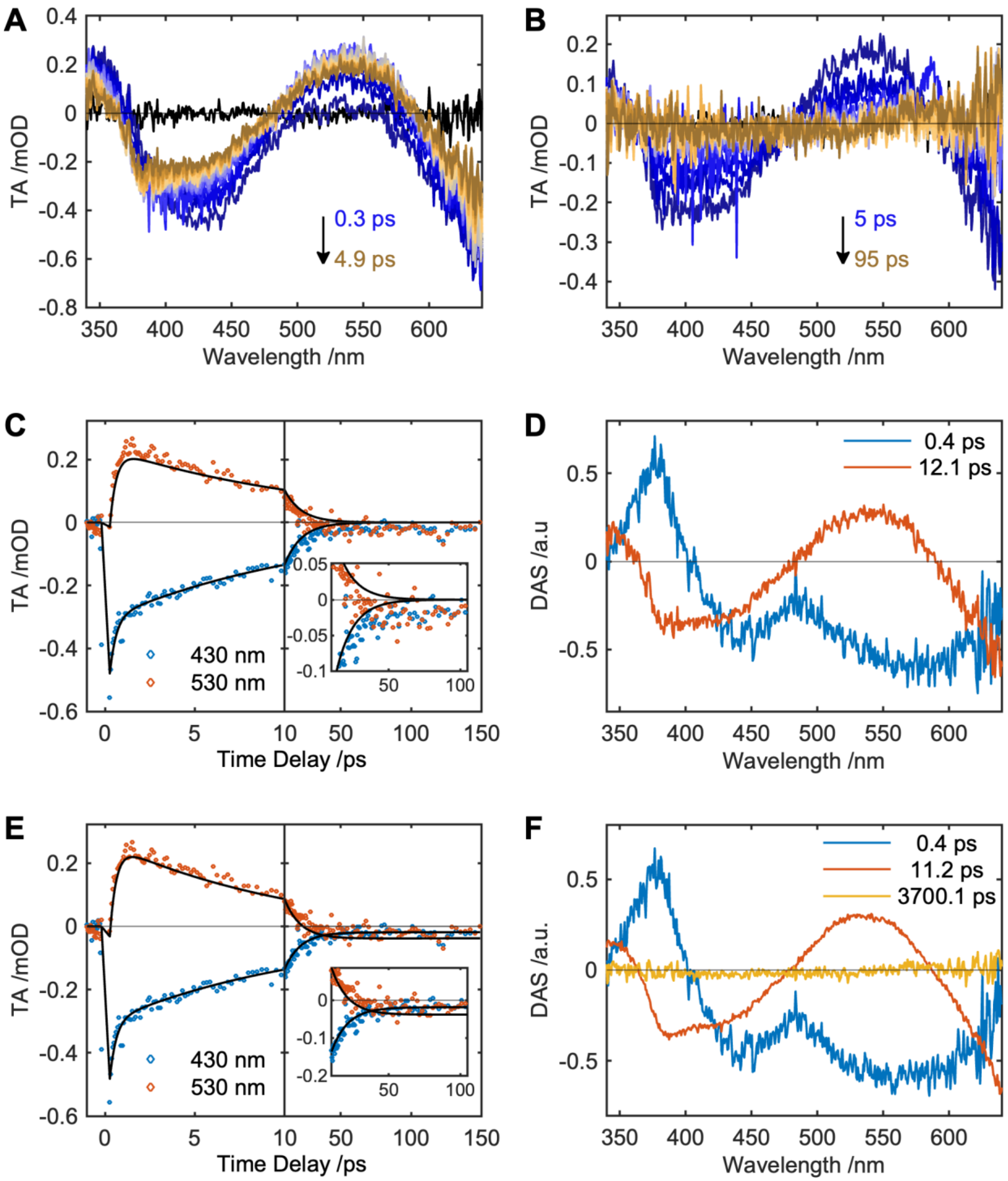

Figure S12. TA data for **1** in THF following 700 nm, 0.3 μJ photoexcitation. (A,B) Cascaded difference spectra across the two time regions indicated. (C,D) and (E,F) show results of three and four component global fits, respectively. (C,E) Kinetic traces at indicative wavelengths across two time regions. Inset is an enlarged view of the traces at long times. (D,F) DAS corresponding to the time constants indicated in the legend.

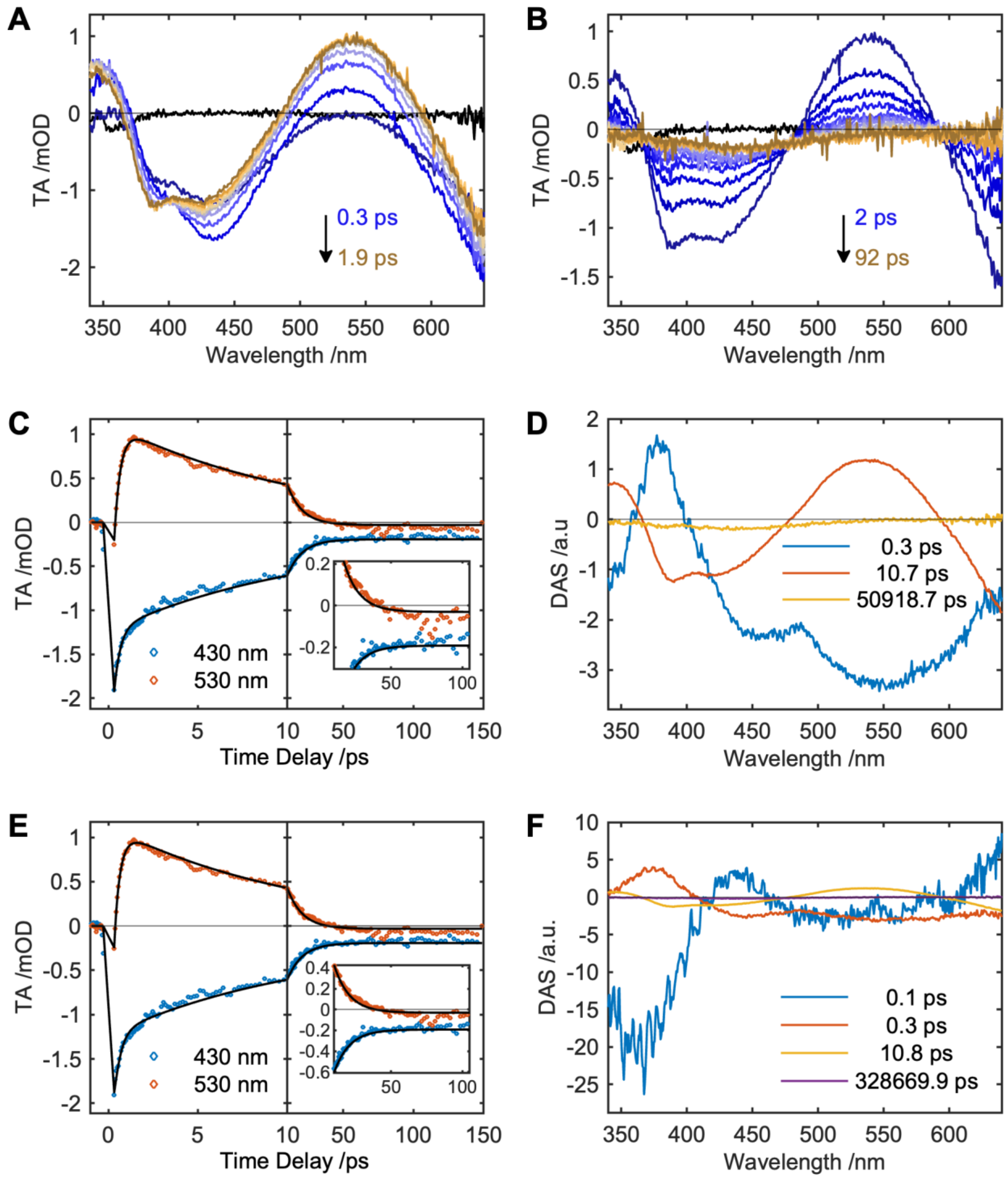

Figure S13. TA data for **1** in THF following 700 nm, 1.5 μJ photoexcitation. (A,B) Cascaded difference spectra across the two time regions indicated. (C,D) and (E,F) show results of three and four component global fits, respectively. (C,E) Kinetic traces at indicative wavelengths across two time regions. Inset is an enlarged view of the traces at long times. (D,F) DAS corresponding to the time constants indicated in the legend.

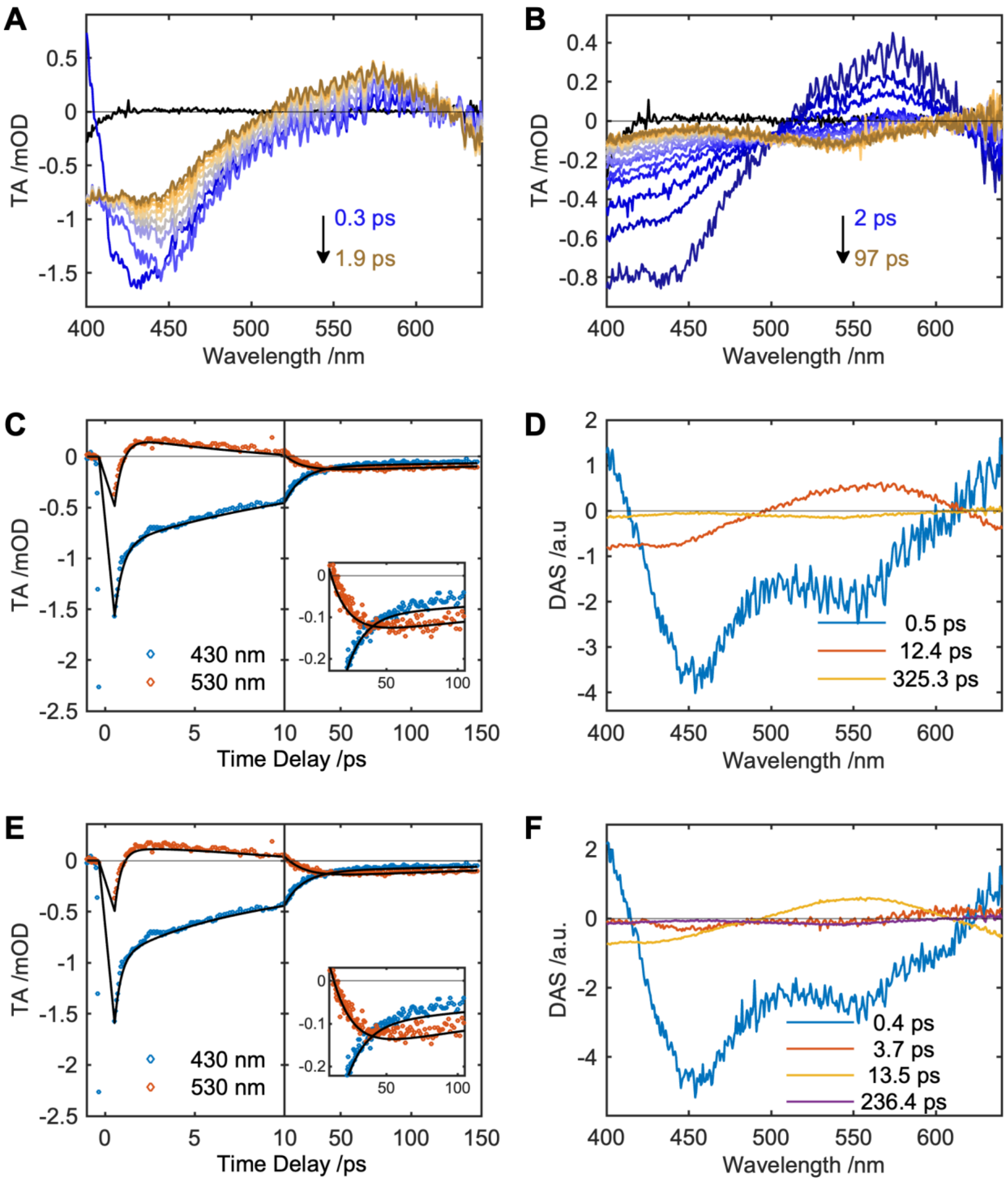

Figure S14. TA data for **1** in toluene following 700 nm, 1 μJ photoexcitation. (A,B) Cascaded difference spectra across the two time regions indicated. (C,D) and (E,F) show results of three and four component global fits, respectively. (C,E) Kinetic traces at indicative wavelengths across two time regions. Inset is an enlarged view of the traces at long times. (D,F) DAS corresponding to the time constants indicated in the legend.

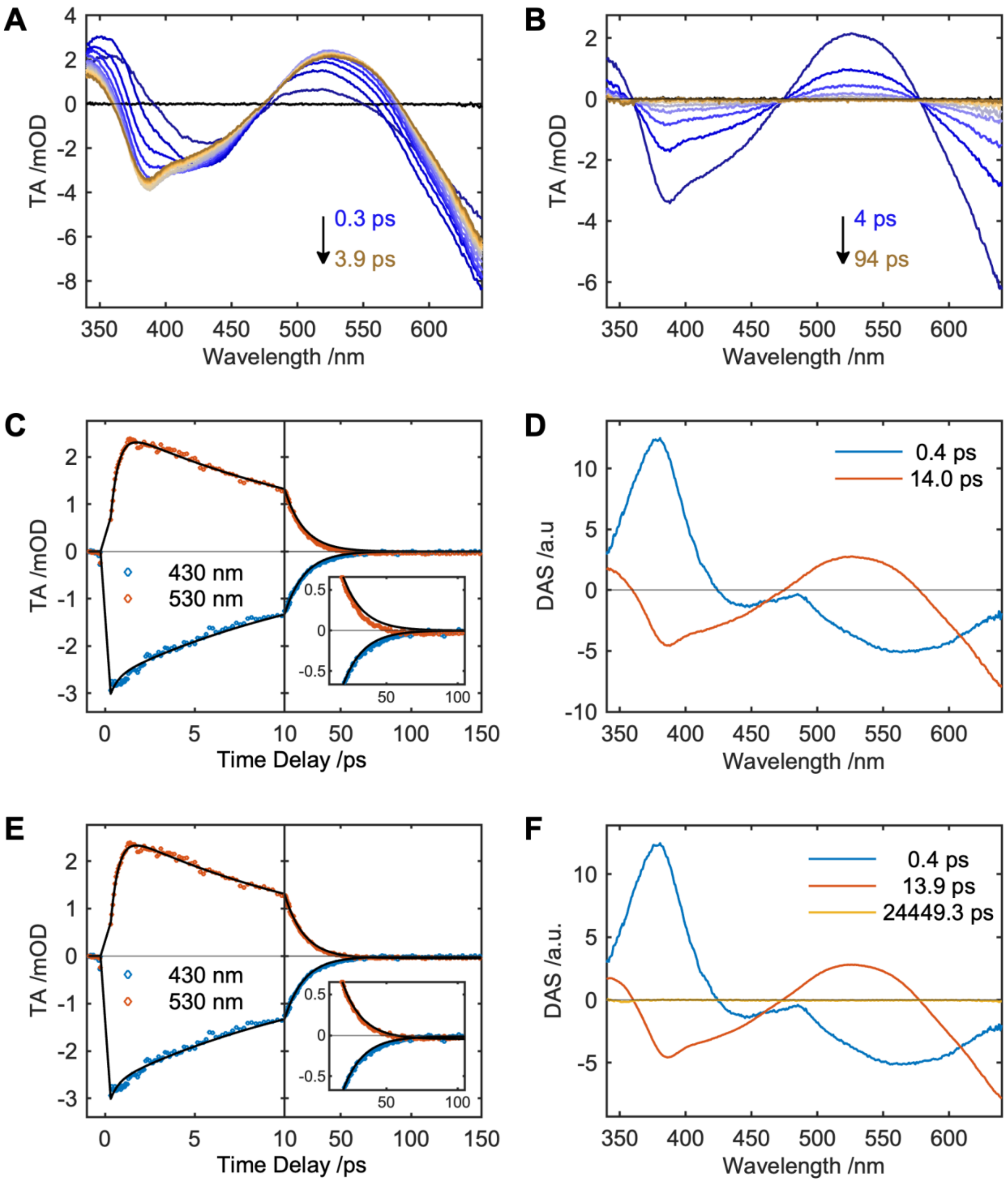

Figure S15. TA data for **1-Br** in THF following 700 nm, 1 μJ photoexcitation. (A,B) Cascaded difference spectra across the two time regions indicated. (C,D) and (E,F) show results of three and four component global fits, respectively. (C,E) Kinetic traces at indicative wavelengths across two time regions. Inset is an enlarged view of the traces at long times. (D,F) DAS corresponding to the time constants indicated in the legend.

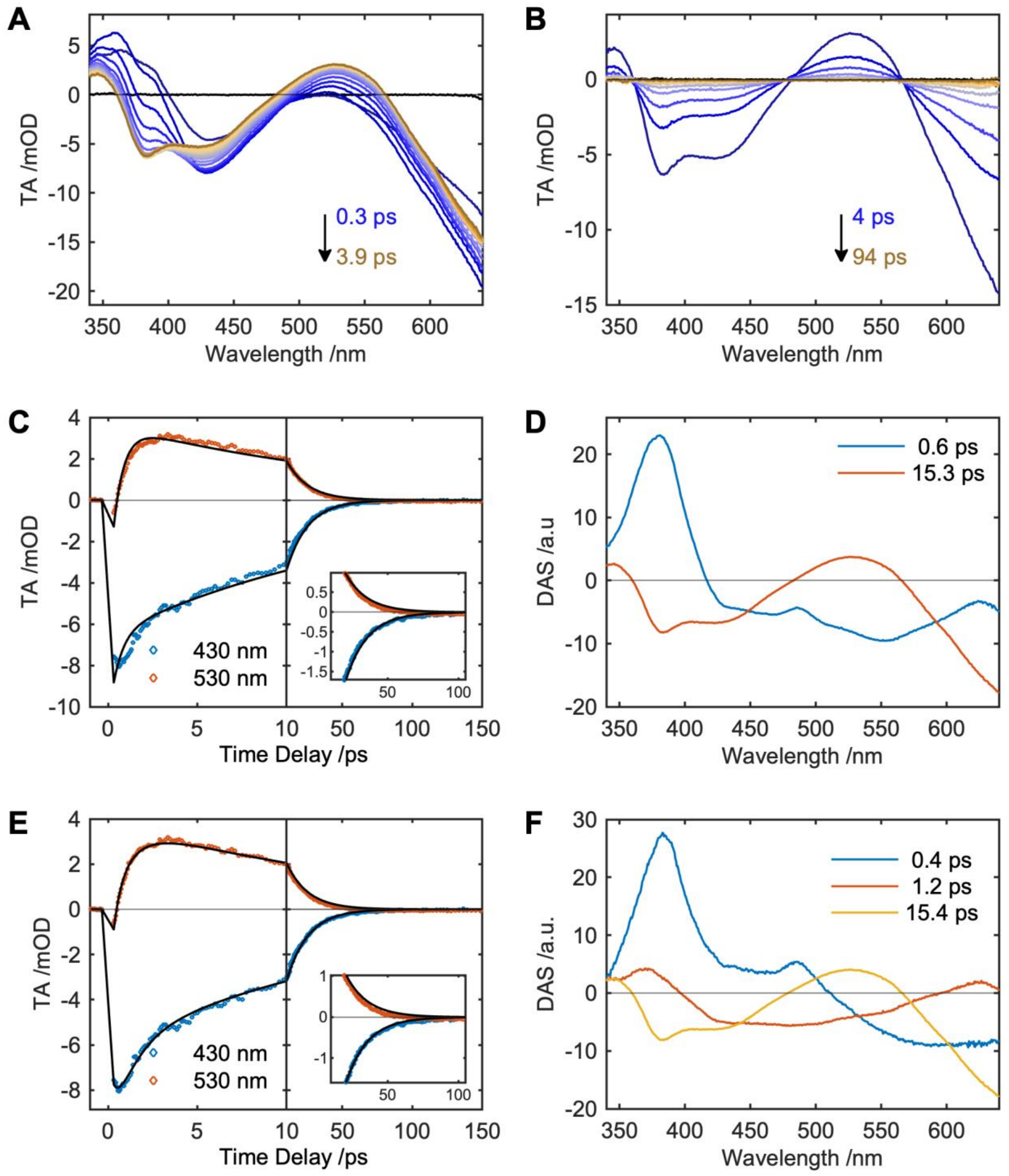

Figure S16. TA data for **1-I** in THF following 700 nm, 1 μJ photoexcitation. (A,B) Cascaded difference spectra across the two time regions indicated. (C,D) and (E,F) show results of three and four component global fits, respectively. (C,E) Kinetic traces at indicative wavelengths across two time regions. Inset is an enlarged view of the traces at long times. (D,F) DAS corresponding to the time constants indicated in the legend.

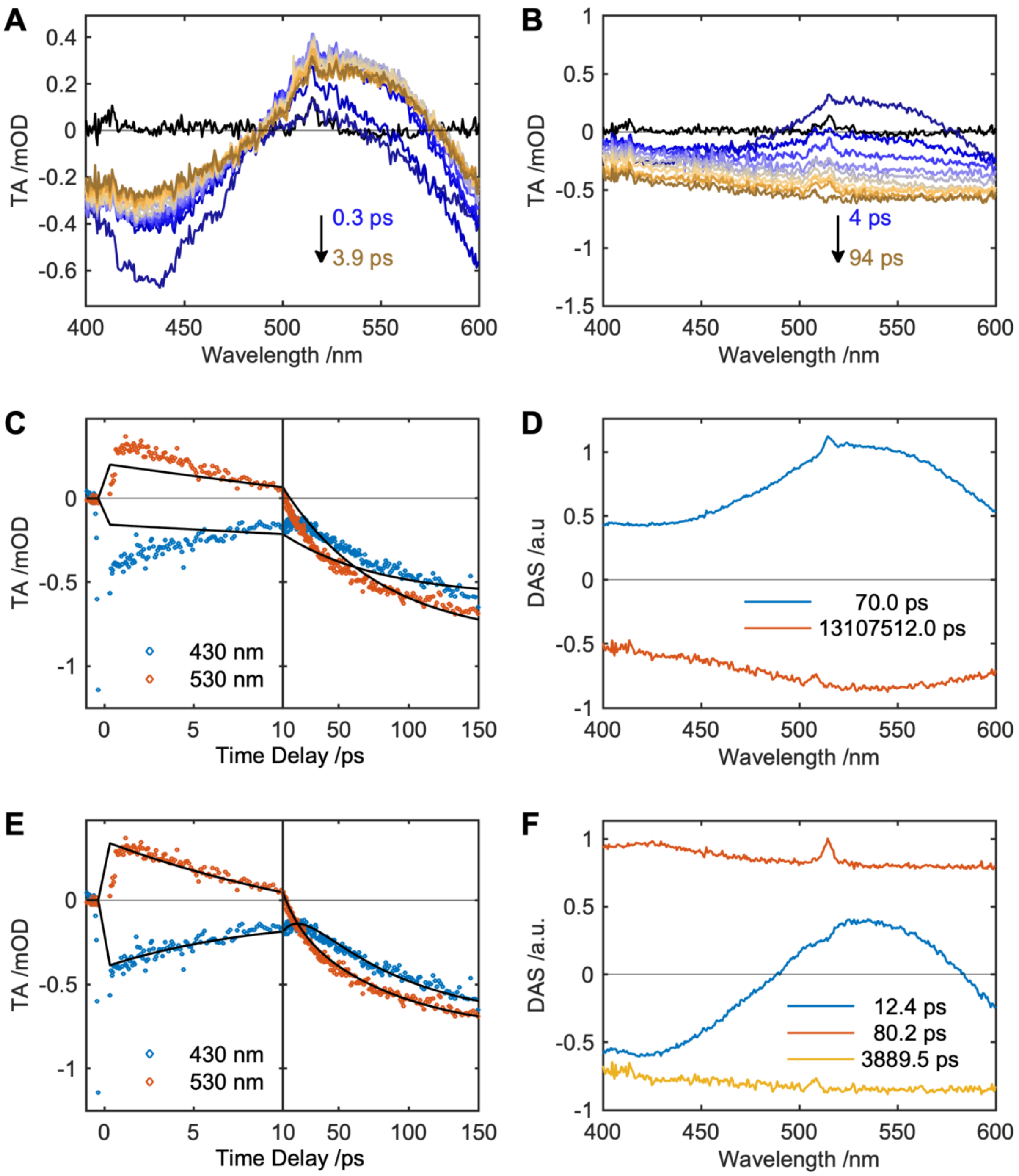

Figure S17. TA data for **2** in THF following 700 nm, 1 μJ photoexcitation. (A,B) Cascaded difference spectra across the two time regions indicated. (C,D) and (E,F) show results of three and four component global fits, respectively. (C,E) Kinetic traces at indicative wavelengths across two time regions. Inset is an enlarged view of the traces at long times. (D,F) DAS corresponding to the time constants indicated in the legend. Peak at 510 nm is a result of pump scatter.

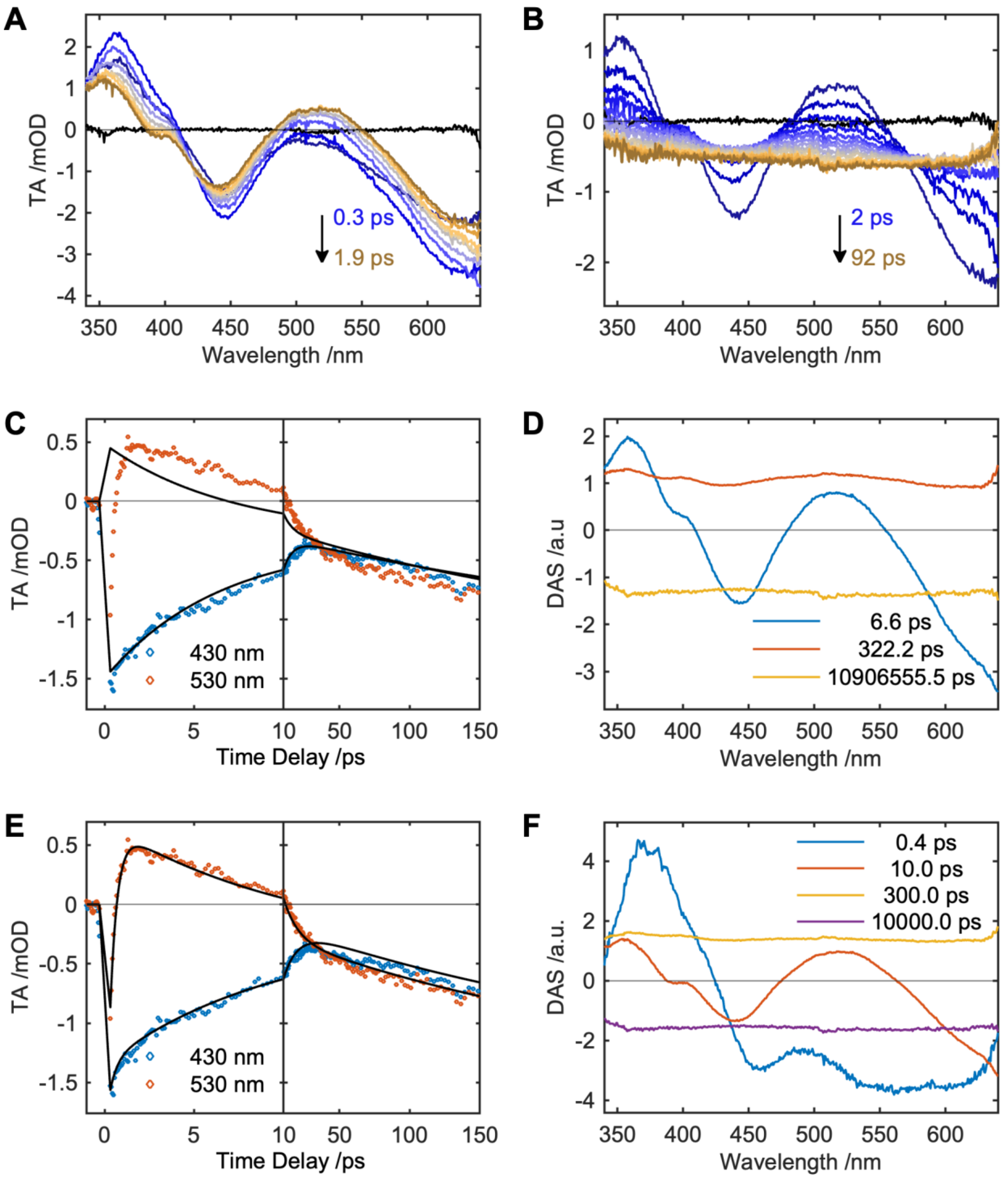

Figure S18. TA data for **3** in THF following 700 nm, 1 μJ photoexcitation. (A,B) Cascaded difference spectra across the two time regions indicated. (C,D) and (E,F) show results of three and four component global fits, respectively. (C,E) Kinetic traces at indicative wavelengths across two time regions. Inset is an enlarged view of the traces at long times. (D,F) DAS corresponding to the time constants indicated in the legend.

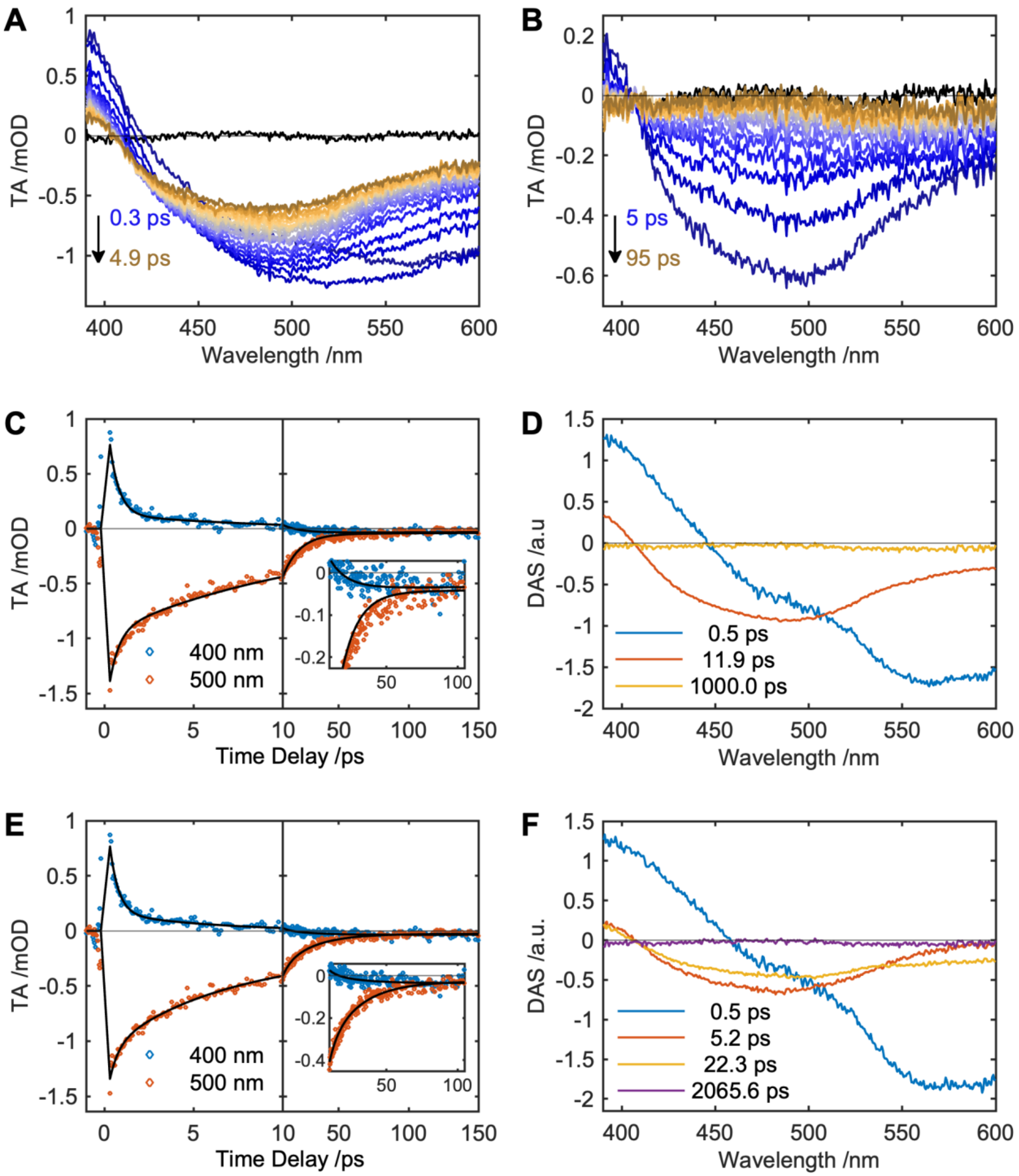

Figure S19. TA data for **4** in THF following 700 nm, 1 μJ photoexcitation. (A,B) Cascaded difference spectra across the two time regions indicated. (C,D) and (E,F) show results of three and four component global fits, respectively. (C,E) Kinetic traces at indicative wavelengths across two time regions. Inset is an enlarged view of the traces at long times. (D,F) DAS corresponding to the time constants indicated in the legend.

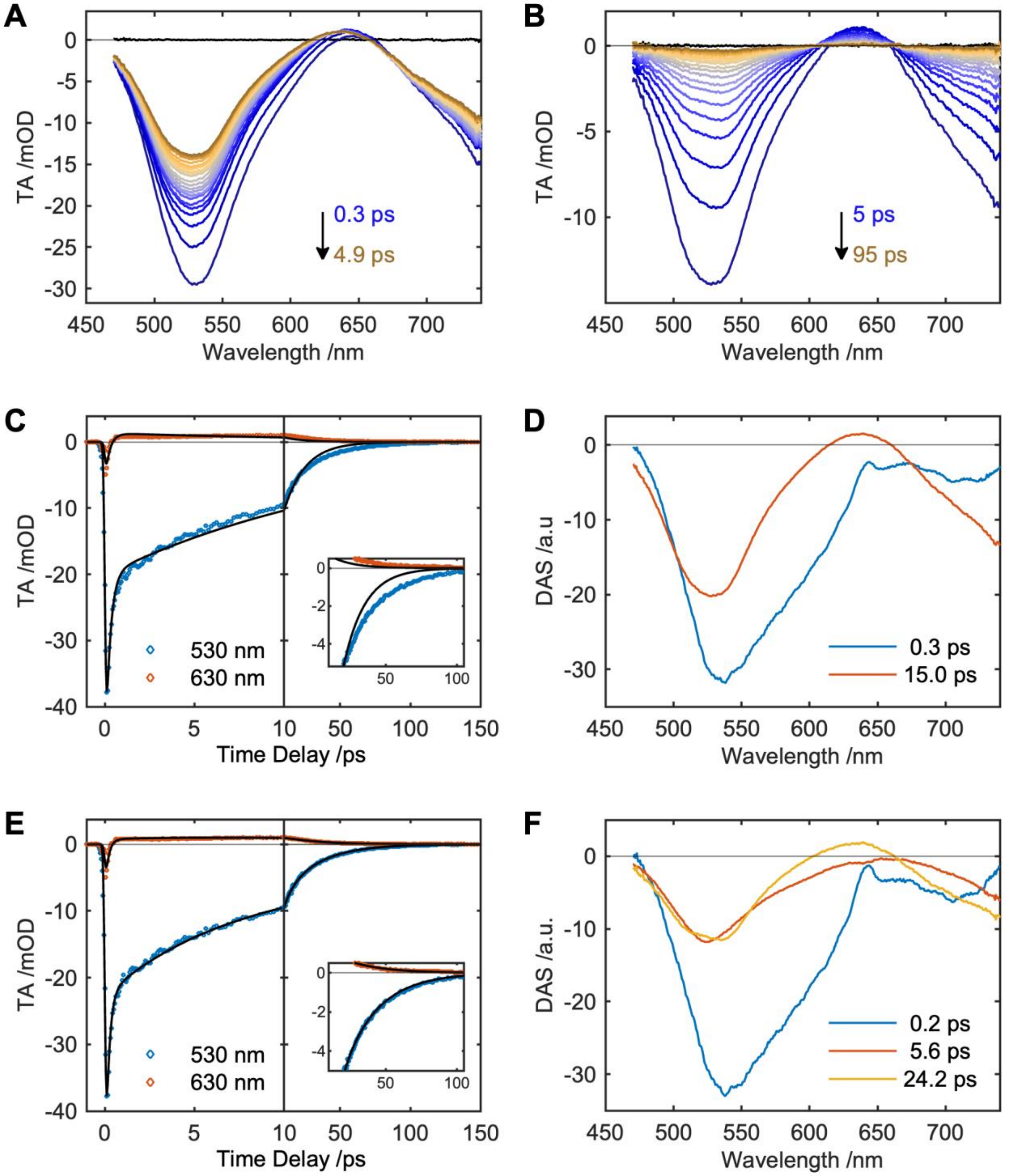

Figure S20. TA data for **5** in THF following 800 nm, 1 μJ photoexcitation. (A,B) Cascaded difference spectra across the two time regions indicated. (C,D) and (E,F) show results of three and four component global fits, respectively. (C,E) Kinetic traces at indicative wavelengths across two time regions. Inset is an enlarged view of the traces at long times. (D,F) DAS corresponding to the time constants indicated in the legend.

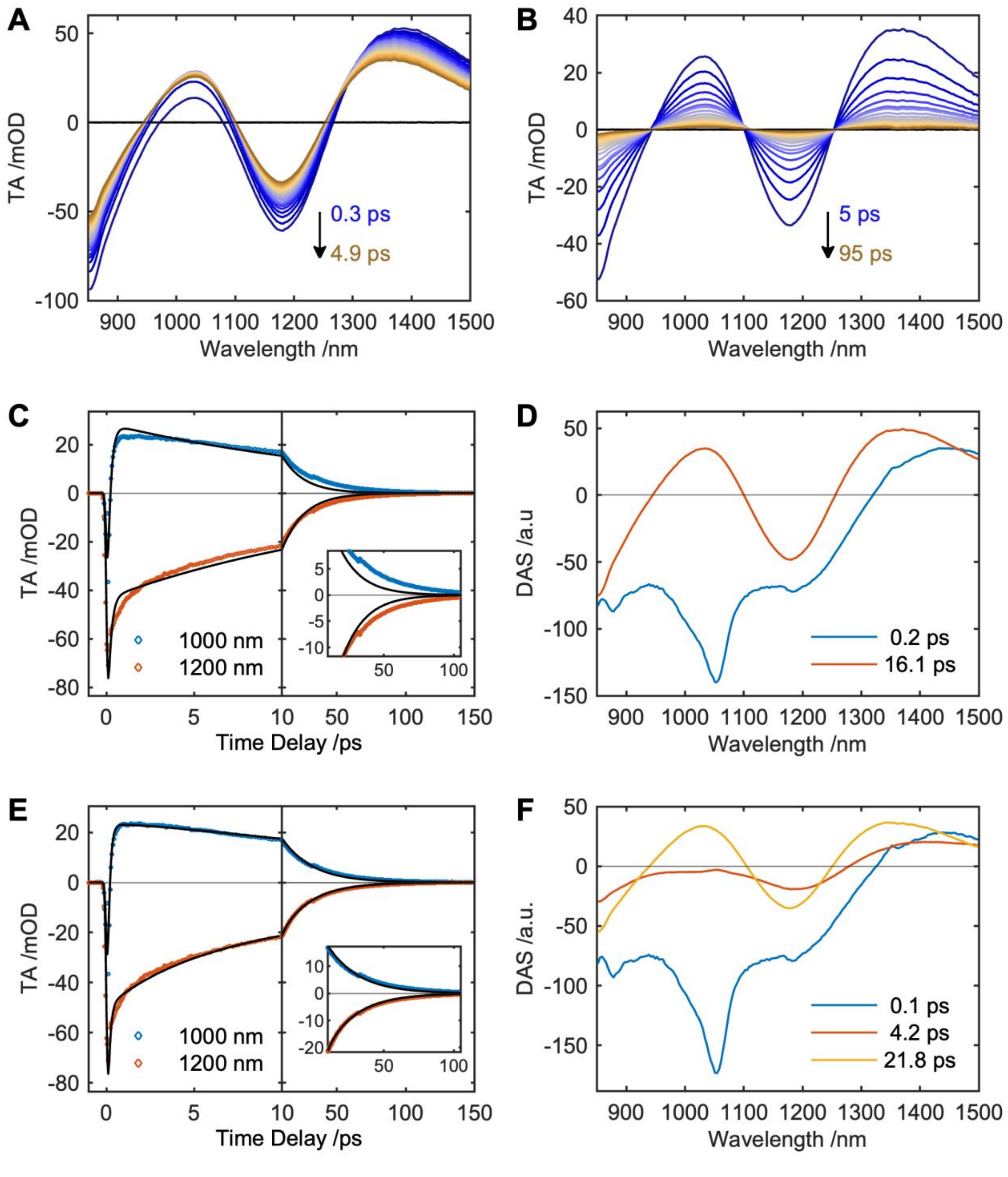

Figure S21. TA data for **5** in THF following 800 nm, 1 µJ photoexcitation. (A,B) Cascaded difference spectra across the two time regions indicated. (C,D) and (E,F) show results of three and four component global fits, respectively. (C,E) Kinetic traces at indicative wavelengths across two time regions. Inset is an enlarged view of the traces at long times. (D,F) DAS corresponding to the time constants indicated in the legend.

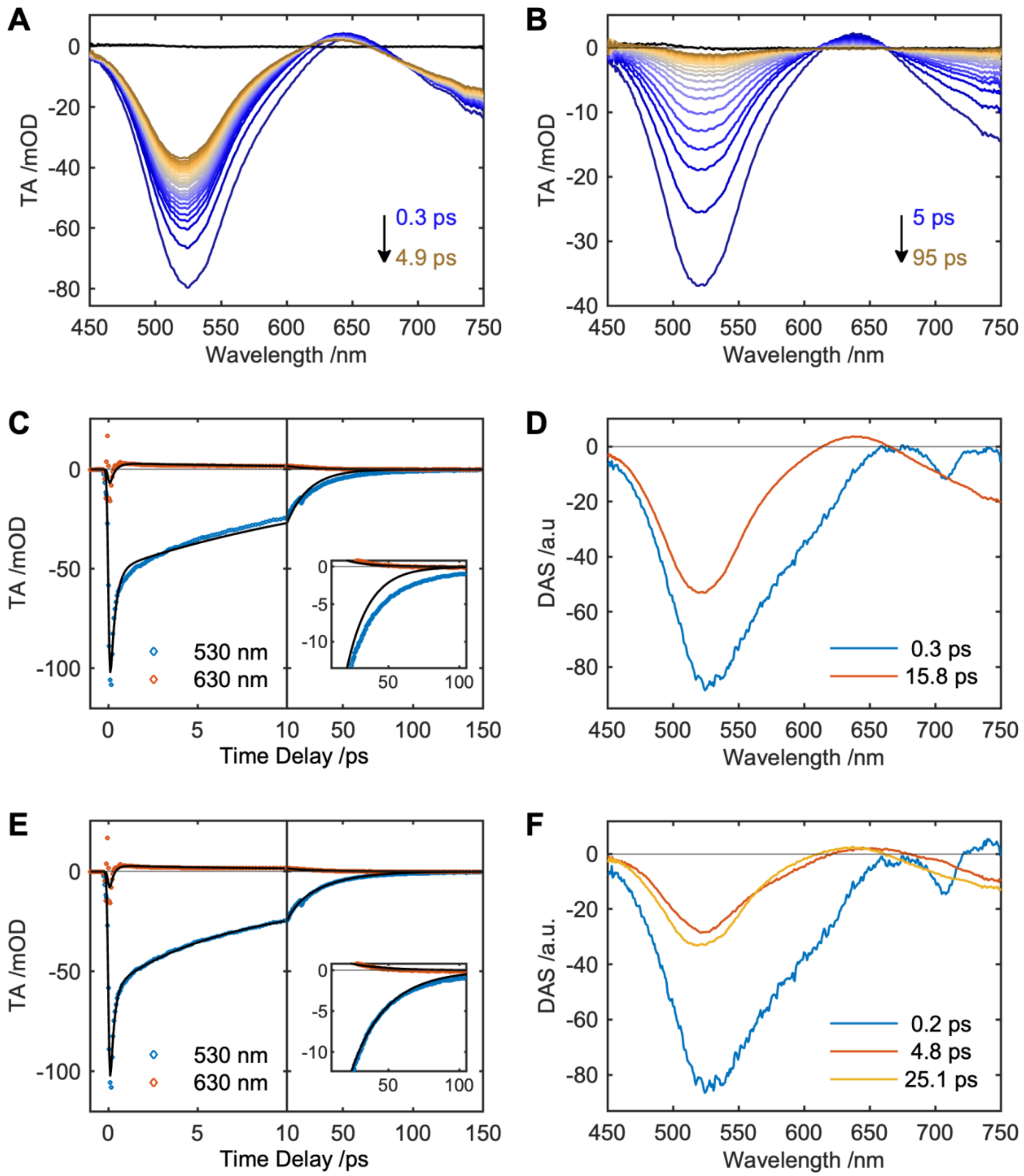

Figure S22. TA data for **5** in THF following 1200 nm photoexcitation. (A,B) Cascaded difference spectra across the two time regions indicated. (C,D) and (E,F) show results of three and four component global fits, respectively. (C,E) Kinetic traces at indicative wavelengths across two time regions. Inset is an enlarged view of the traces at long times. (D,F) DAS corresponding to the time constants indicated in the legend.

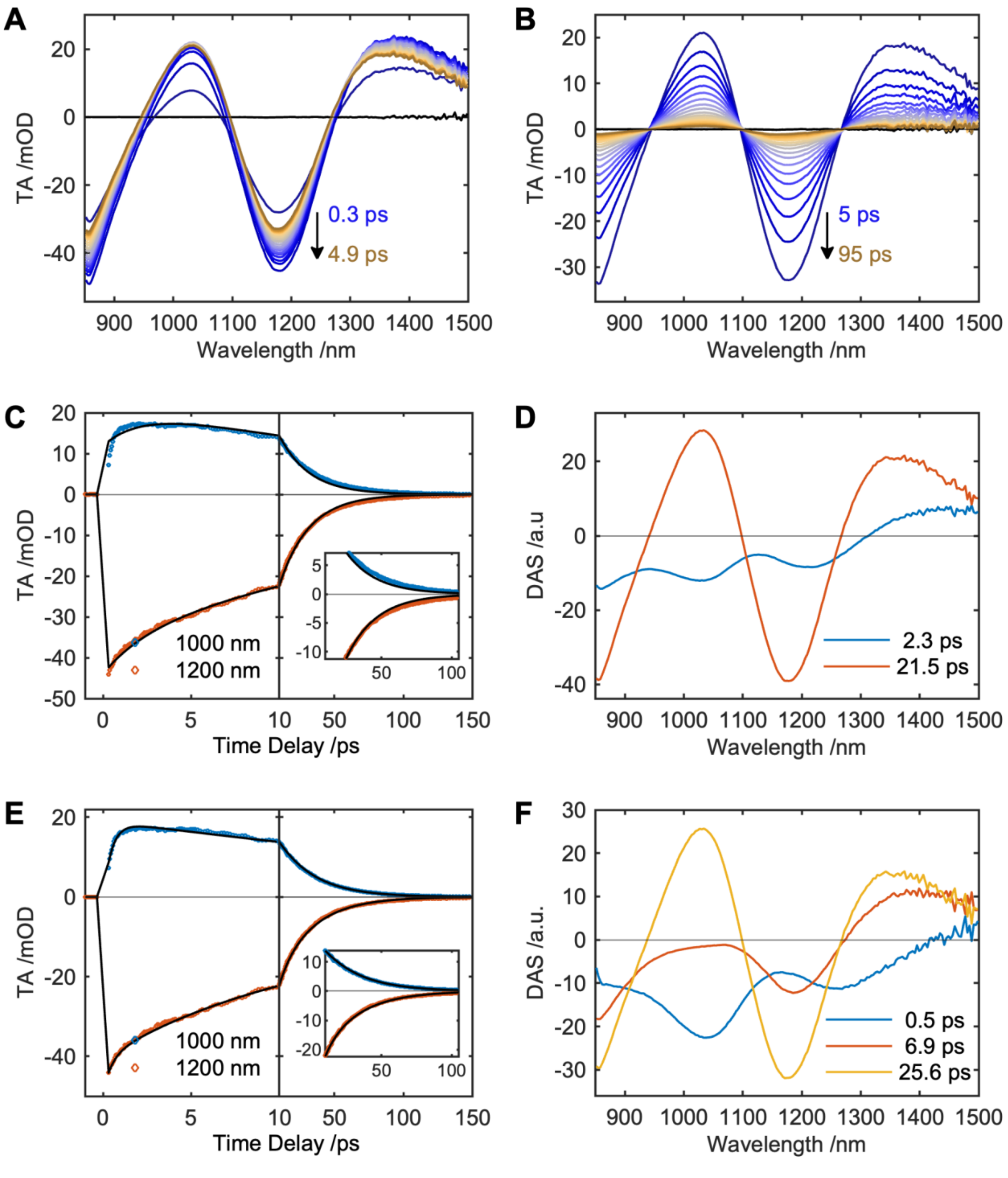

Figure S23. TA data for **5** in THF following 560 nm, 1 μJ photoexcitation. (A,B) Cascaded difference spectra across the two time regions indicated. (C,D) and (E,F) show results of three and four component global fits, respectively. (C,E) Kinetic traces at indicative wavelengths across two time regions. Inset is an enlarged view of the traces at long times. (D,F) DAS corresponding to the time constants indicated in the legend. The peak at ~1120 nm is due to scattered pump light.

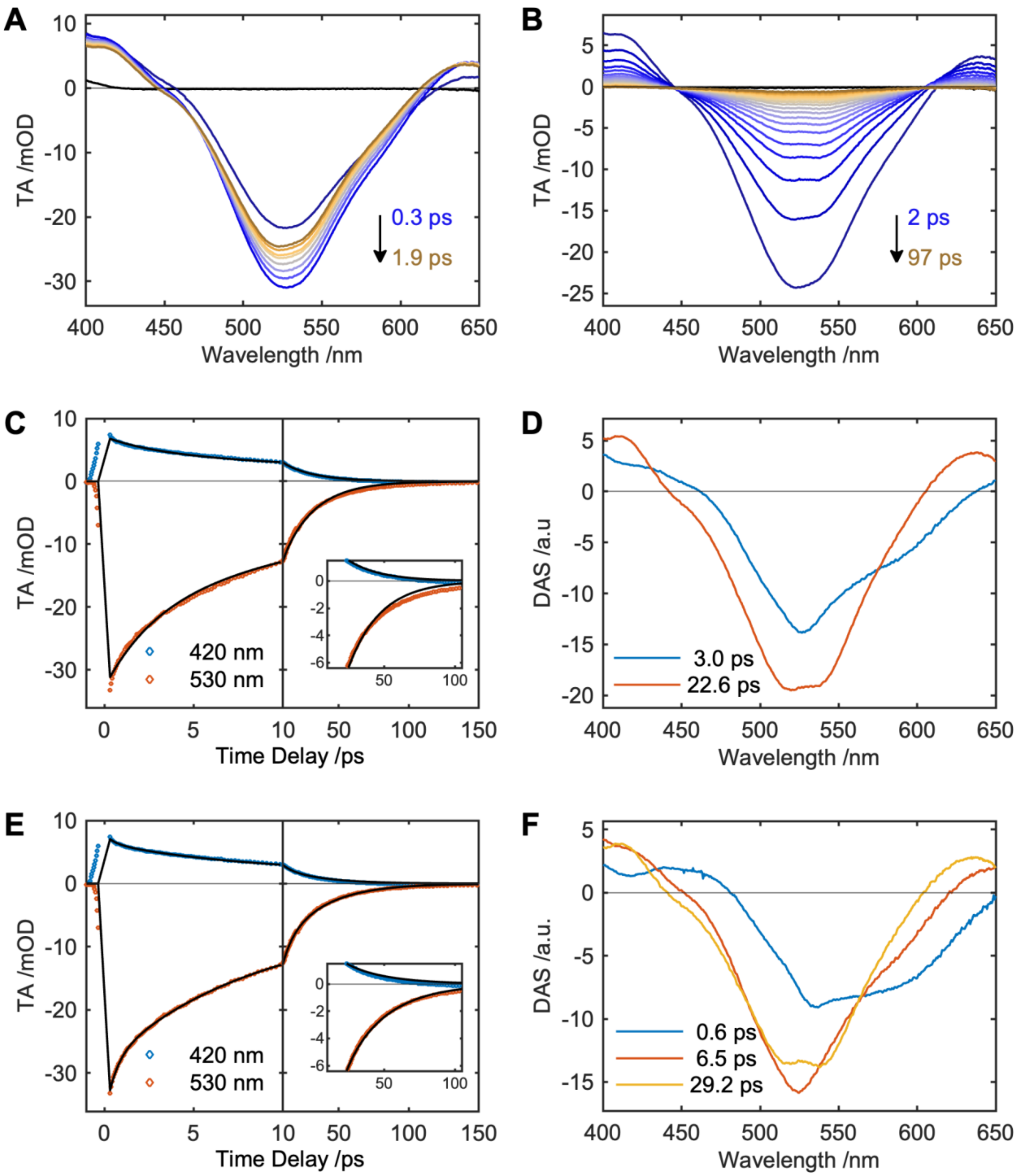

Figure S24. TA data for **5** in toluene following 800 nm, 1 μJ photoexcitation. (A,B) Cascaded difference spectra across the two time regions indicated. (C,D) and (E,F) show results of three and four component global fits, respectively. (C,E) Kinetic traces at indicative wavelengths across two time regions. Inset is an enlarged view of the traces at long times. (D,F) DAS corresponding to the time constants indicated in the legend.

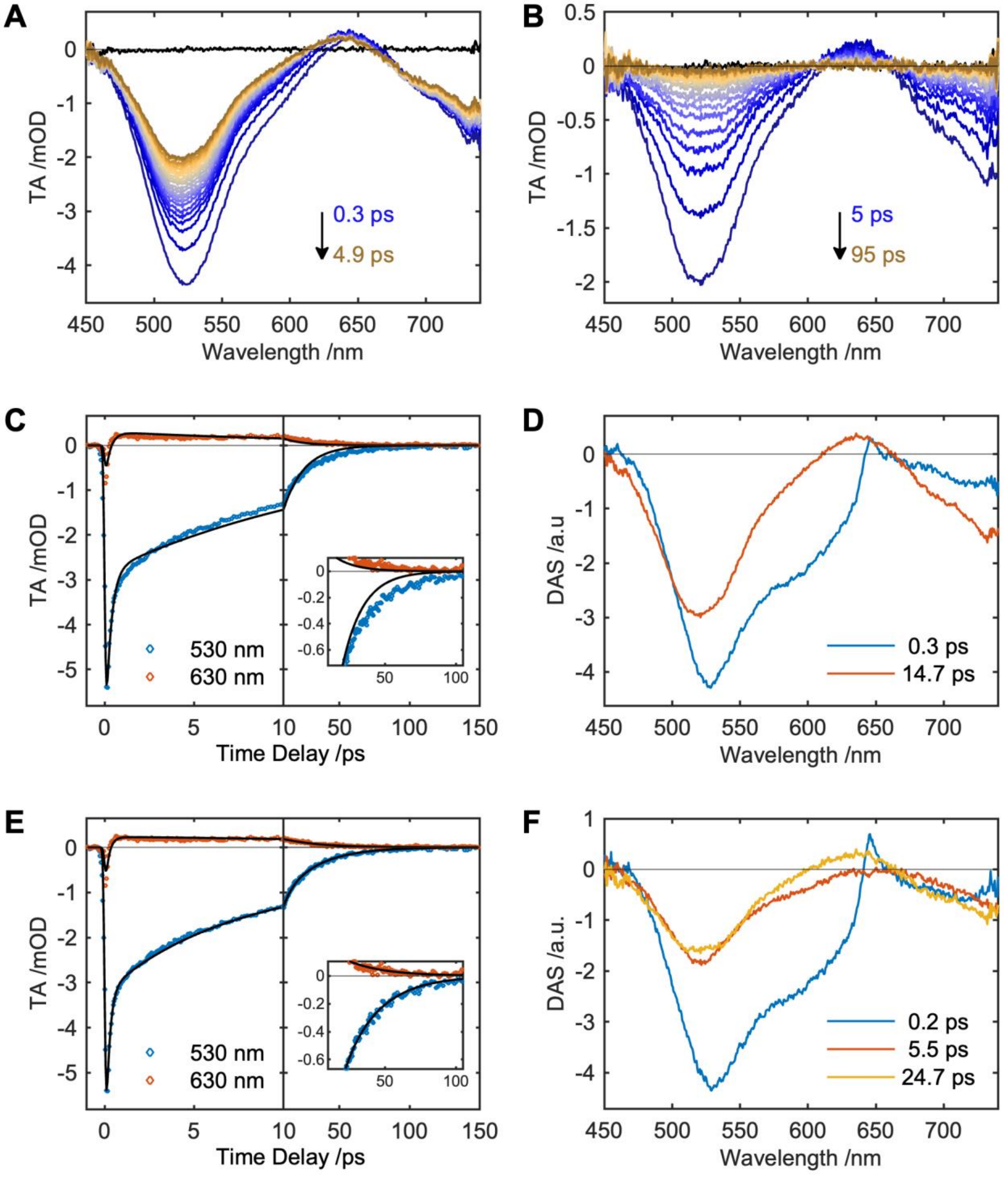

Figure S25. TA data for **5** in THF following 800 nm, 0.15 μJ photoexcitation. (A,B) Cascaded difference spectra across the two time regions indicated. (C,D) and (E,F) show results of three and four component global fits, respectively. (C,E) Kinetic traces at indicative wavelengths across two time regions. Inset is an enlarged view of the traces at long times. (D,F) DAS corresponding to the time constants indicated in the legend.

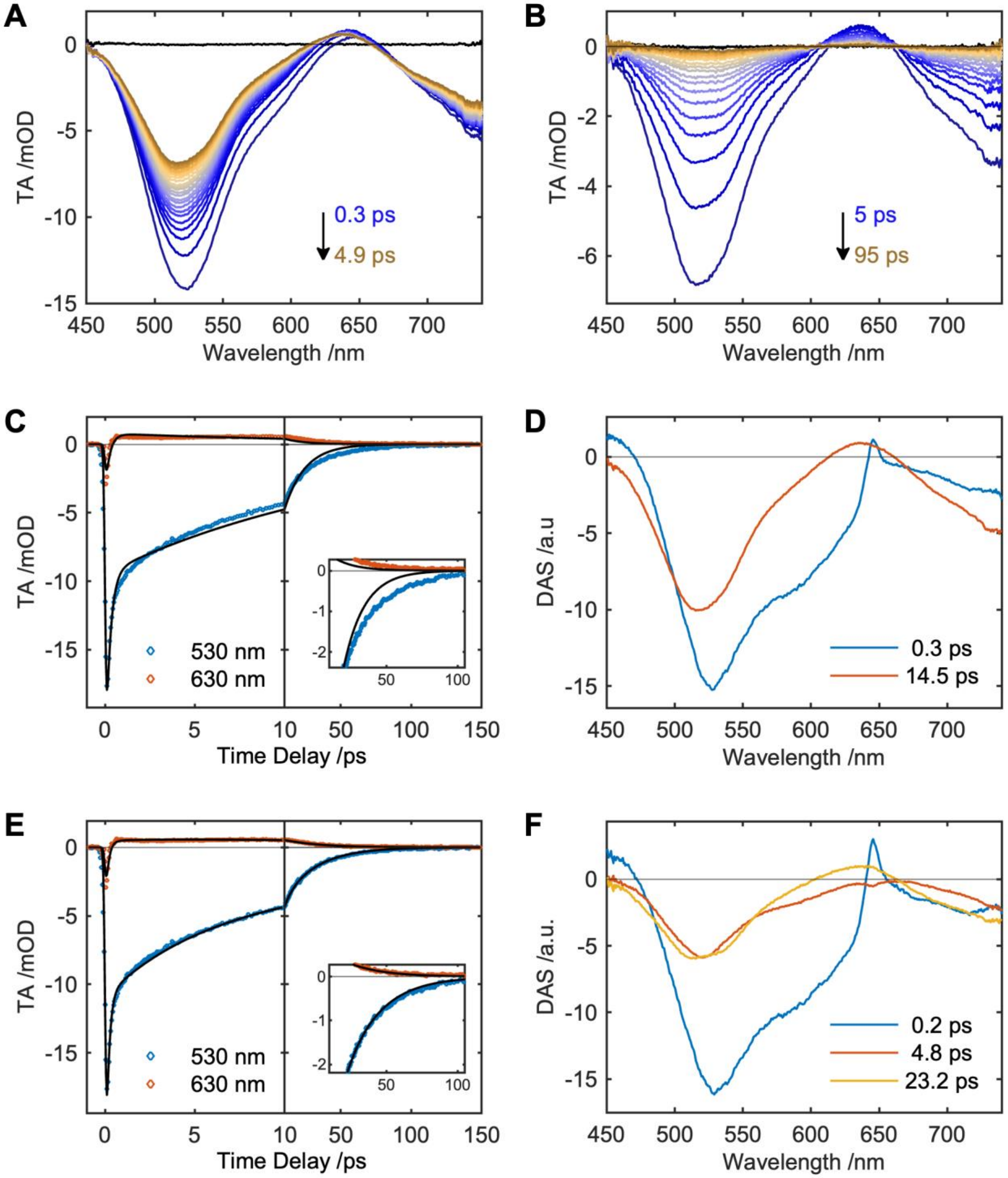

Figure S26. TA data for **5** in THF following 800 nm, 0.3 μJ photoexcitation. (A,B) Cascaded difference spectra across the two time regions indicated. (C,D) and (E,F) show results of three and four component global fits, respectively. (C,E) Kinetic traces at indicative wavelengths across two time regions. Inset is an enlarged view of the traces at long times. (D,F) DAS corresponding to the time constants indicated in the legend.

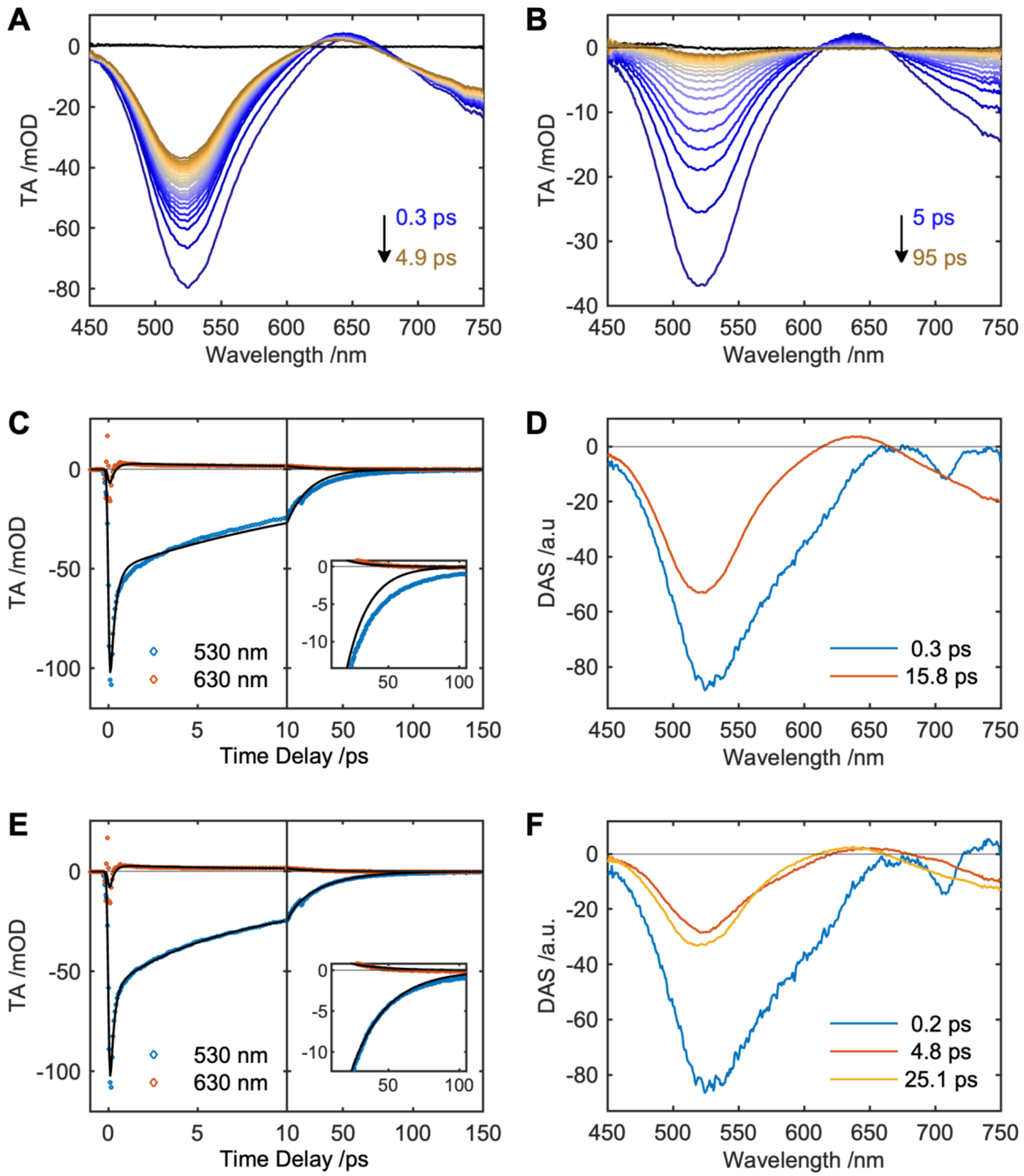

Figure S27. TA data for **5** in THF following 800 nm, 2 μJ photoexcitation. (A,B) Cascaded difference spectra across the two time regions indicated. (C,D) and (E,F) show results of three and four component global fits, respectively. (C,E) Kinetic traces at indicative wavelengths across two time regions. Inset is an enlarged view of the traces at long times. (D,F) DAS corresponding to the time constants indicated in the legend.

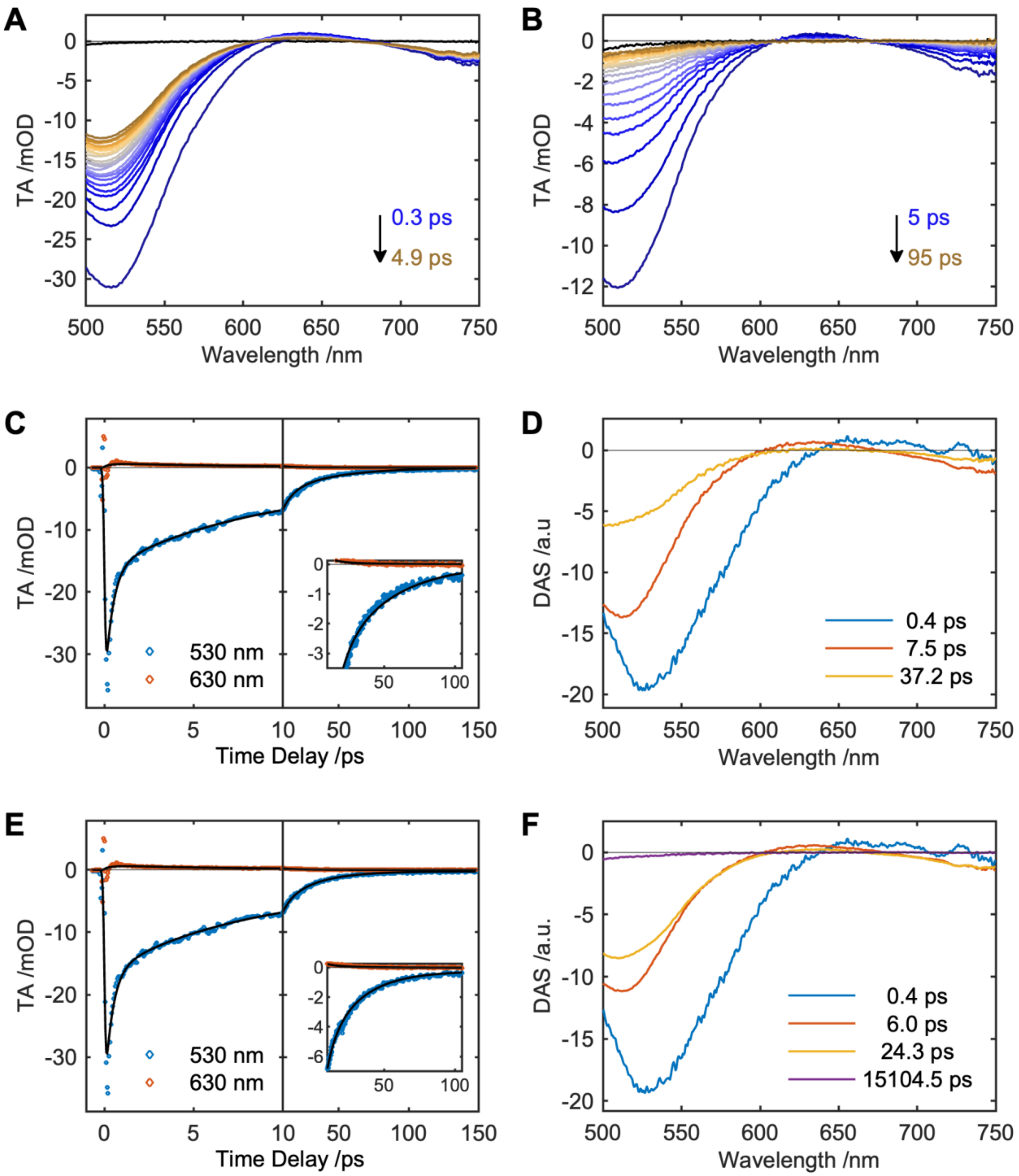

Figure S28. TA data for **5′** in THF following 1200 nm photoexcitation. (A,B) Cascaded difference spectra across the two time regions indicated. (C,D) and (E,F) show results of three and four component global fits, respectively. (C,E) Kinetic traces at indicative wavelengths across two time regions. Inset is an enlarged view of the traces at long times. (D,F) DAS corresponding to the time constants indicated in the legend.

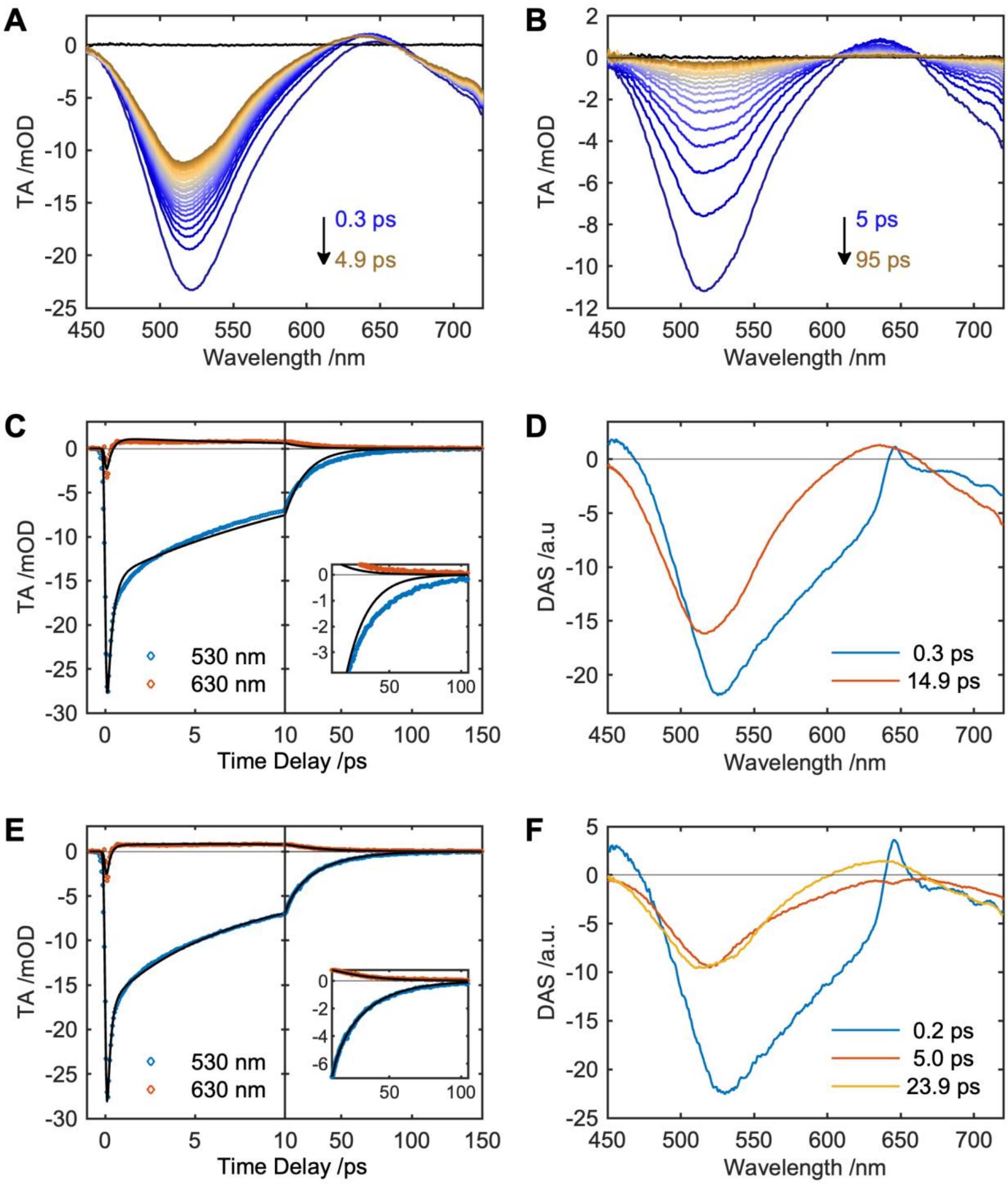

Figure S29. TA data for **5′** in THF following 800 nm, 1 μJ photoexcitation. (A,B) Cascaded difference spectra across the two time regions indicated. (C,D) and (E,F) show results of three and four component global fits, respectively. (C,E) Kinetic traces at indicative wavelengths across two time regions. Inset is an enlarged view of the traces at long times. (D,F) DAS corresponding to the time constants indicated in the legend.

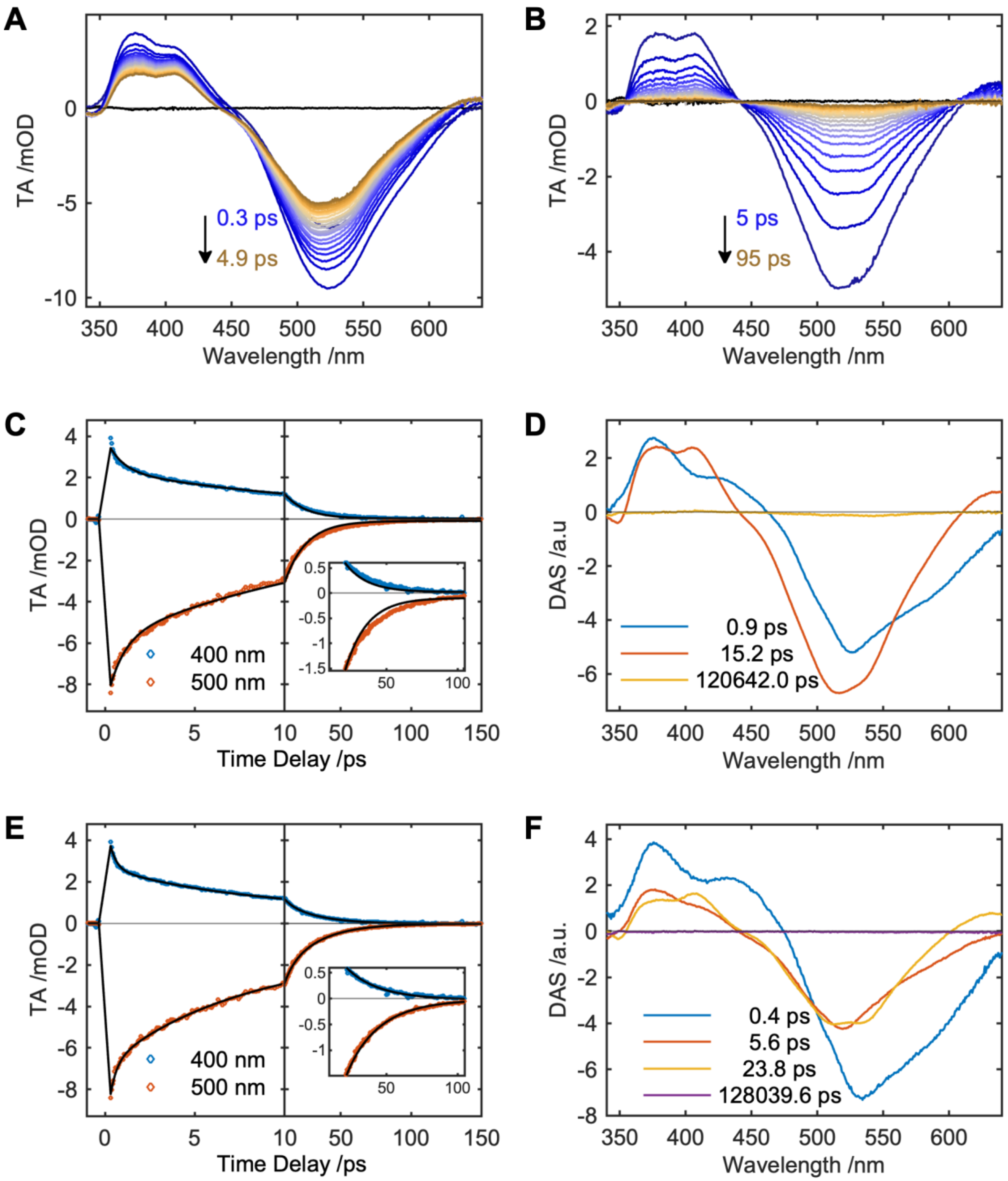

Figure S30. TA data for **5-Br** in THF following 800 nm, 1 μJ photoexcitation. (A,B) Cascaded difference spectra across the two time regions indicated. (C,D) and (E,F) show results of three and four component global fits, respectively. (C,E) Kinetic traces at indicative wavelengths across two time regions. Inset is an enlarged view of the traces at long times. (D,F) DAS corresponding to the time constants indicated in the legend.

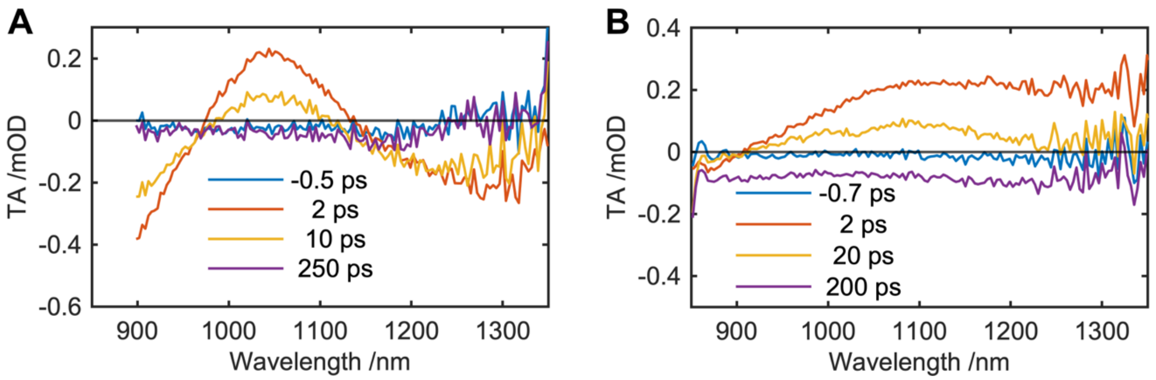

Figure S31. NIR difference spectra for (A) **4** and (B) **5-Br** at selected time delays following 700 nm and 800nm, 1 μJ photoexcitation, respectively. The GSB is partially obscured by the overlapping ESA in **4**. Noise precluded reliable fitting, but the features are shown for comparison to other measurements.

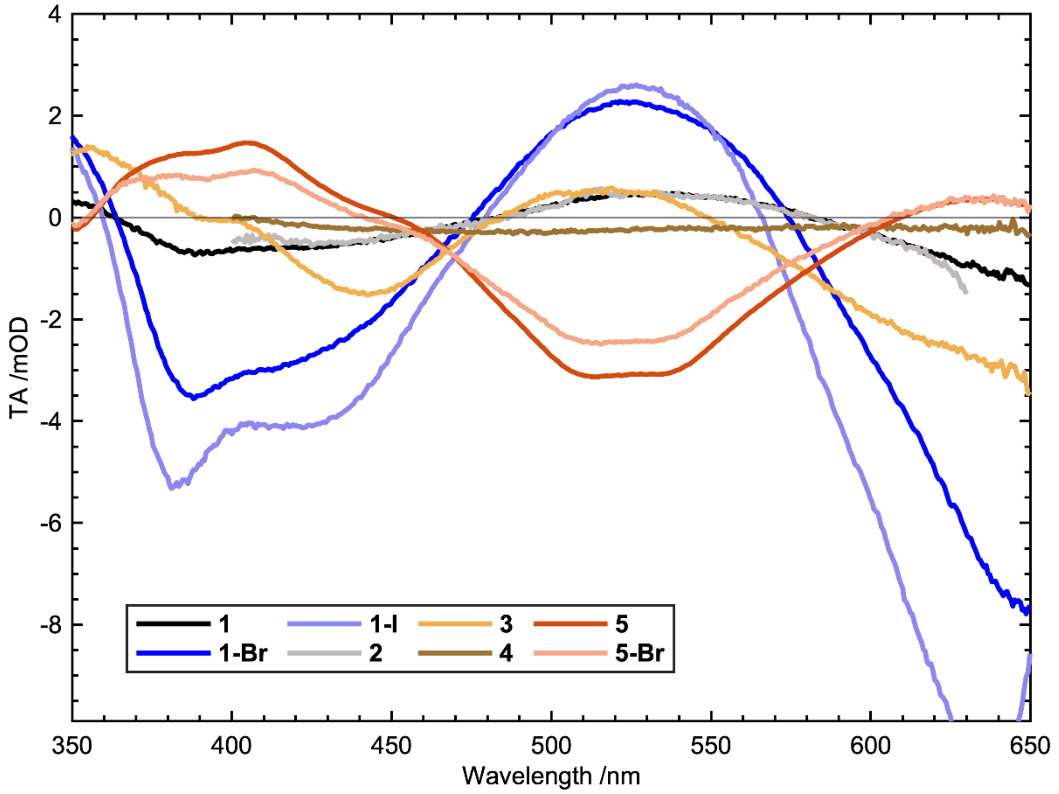

Figure S32. Difference spectra for all compounds overlaid following 1 μJ photoexcitation at either 700 or 800 nm. Time delays chosen to represent difference spectrum of rate-limiting process: compounds **1**, **1-Br**, **2** and **3** are shown at 2 ps, **1-I** at 5 ps, and **4**, **5** and **5-Br** at 15 ps. The spectrum for **5** has been scaled by 0.1 to fit on the same y-axis as the other compounds.

In this section, we have presented TA spectra measured for each compound (Figures S11-S31) using the procedure described in Section S1. Where possible, each dataset is shown with fits to

two kinetic models. The parameters obtained from the better of these two fits are given in Table S3 below.

TA spectra were also measured for **5** following photoexcitation into its MLCT bands at 1200 and 560 nm (Figures S22-S23). The resulting spectra and time constants agree well with the results for 800 nm photoexcitation, indicating the difference in behavior between **1** and **5** is not the result of pumping different MLCT bands; the lowest-energy MLCT dictates the overall ultrafast behavior.

We additionally questioned if solvent coordination to Ni obfuscated the comparison between **1** and **5**. Thus, TA spectra were also measured in toluene. The spectral and kinetic profiles of **1** and **5** were largely unchanged (still requiring two- and three-component fits, respectively) and both exhibited slightly slower relaxation to the ground state (12.4 ps in **1** and 29.2 ps in **5**). The difference in kinetic behavior between **1** and **5** is therefore not due to solvent coordination; we do not observe any evidence of a difference in relaxation pathway between solvents.

Finally, we repeated the above TA measurements on **1** and **5** at a range of pump powers to ensure the observed differences in dynamics were not due to saturation or multi-photon effects. For both **1** and **5**, the kinetics were independent of pump power from 0.15 to 2 mW (Table S3).

Table S3. Table of relaxation time constants for all compounds in THF.

Compound	Pump Wavelength (nm)	Pump Power (μJ)	Solvent	τ_1 (ps)	τ_2 (ps)	τ_3 (ps)	τ_4 (ps)[a]	τ_5 (ps)[a]
**1**	700	1.0	THF	0.3	-	10.9	-	∞
**1**	700	0.3	THF	0.4	-	11.2	-	∞
**1**	700	1.5	THF	0.3	-	10.7	-	∞
**1**	700	1.0	Toluene	0.5	-	12.4	-	∞
**1-Br**	700	1.0	THF	0.4	-	13.9	-	∞
**1-I**	700	1.0	THF	0.4	1.2	15.4	-	-
**2**	700	1.0	THF	-	-	12	80	∞
**3**	700	1.0	THF	0.4	-	10	300	∞
**4**	800	1.0	THF	0.5	5.2	22.3	-	∞
**5**	800	1.0	THF	0.6	5.6	24.2	-	-
**5**[b]	800	1.0	THF	0.1	4.2	21.8	-	-
**5**	1200	n.d[c]	THF	0.2	4.8	25.1	-	-
**5**[b]	560	1.0	THF	0.5	6.9	25.6	-	-
**5**	800	1.0	Toluene	0.6	6.5	29.2	-	-
**5**	800	0.15	THF	0.2	5.5	24.7	-	-
**5**	800	0.3	THF	0.2	4.8	23.2	-	-
**5**	800	2.0	THF	0.2	4.8	25.1	-	-
**5′**	1200	n.d[c]	THF	0.4	6.0	24.3	-	∞
**5′**	800	1.0	THF	0.2	5.0	23.9	-	-
**5-Br**	800	1.0	THF	0.4	5.6	23.8	-	∞

[a]Time constants correspond to growth of long-lived feature discussed below, with those much longer than the time window considered infinite. [b]Spectra recorded with NIR probe. [c]Not determined due to lack of a suitable NIR power meter.

Discussion of Anomalous Long-lived Feature

As noted in the main text, a long-lived feature is observed in some spectra. This feature is negative and flat with little-to-no shape and appears to increase in magnitude as time-delay increases. The DAS corresponding to the shortest time constants show signals that clearly correspond to the growth or decay of the GSB and ESA of interest. Given the overlap of the positions of the GSB with the peaks in the static absorbance spectrum, these features must correspond to the Ni(I) species. In contrast, the final time constant corresponds to a constant offset which does not decay on the timescale of the experiment. The lack of any GSB features in the DAS corresponding to the static Ni(I) absorbance show that this signal does not arise from the Ni(I) species. For most compounds, this long-lived signal is very small and has minimal impact on the results other than necessitating an extra constant-offset component in the fitting procedure. However, in the samples containing **3** (Figure S18), this long-lived feature is non-negligible, and four components are needed for a reasonable fit to the data.

To better understand the kinetics of **3**, we turn again to the DAS. The shortest two components clearly correspond to the Ni(I) complex, due to GSB peaks with the same positions as peaks in the steady-state absorbance spectrum. The near-UV band (~350 nm) in the 10 ps DAS is often diagnostic of the presence of reduced bpy (an MLCT state). In contrast, the other two DAS are largely flat, and as such are not included in the analysis in the rest of this work. These latter two time components correspond to the slow growth of the flat feature and persist beyond the experimental timescale. Notably, consecutive repeats of this TA (Figure S33A) overlap exactly, showing that the signal is not due to sample degradation over the course of the experiment. Thus, we find a strong Ni(I) signal which decays alongside a slowly growing, broad, negative feature.

Given that the long-lived signal is largest in **3**, we investigated it in more depth using **3** as a model system. Photogeneration of **3** from its parent Ni(II) complex resulted in the formation of some precipitate. We brought the sample into the glove box, filtered the cuvette solution through a Kimwipe, and returned the filtrate for TA analysis. TA on this solution did not exhibit a noticeable change to the unfiltered sample. We further questioned if this signal was from a Ni(I) complex. Ni(I) species are known to react quickly with aryl halides. In the glove box, aryl bromide was added to the solution. Doing so removed the TA signal originating from the Ni(I) species, but the long-lived signal was still present in the sample (Figure S33B). Therefore, we find that the short-lived signals assigned to **3** do indeed arise from the Ni(I)–bpy halide complex, but that the long-lived signal does not.

We next turned to a photogeneration time-course experiment to try to identify the origin of the signal. A solution of the Ni(II) parent complex for **3** was irradiated for different lengths of time and the TA signal at 1 ns was plotted alongside the UV-vis data (Figure S33C). Pumping the compound at 700 nm avoids exciting any parent Ni(II) left in solution. Furthermore, by looking at only the TA signal at a long time delay (1 ns) wherein all photoexcited **3** will be back in the ground state, we can select for only the long-lived feature. From the absorbance data, the Ni(II) peak (~480 nm) decreases monotonically with time, while the peak around 670 nm grows in and then decreases. This lower energy peak is indicative of the formation and then subsequent thermal degradation of **3**. In the corresponding TA, a monotonic growth of the signal magnitude with increasing irradiation time is observed. The signal presents as a broad feature that slowly grows towards the blue with two small peaks around 350 and 450 nm.

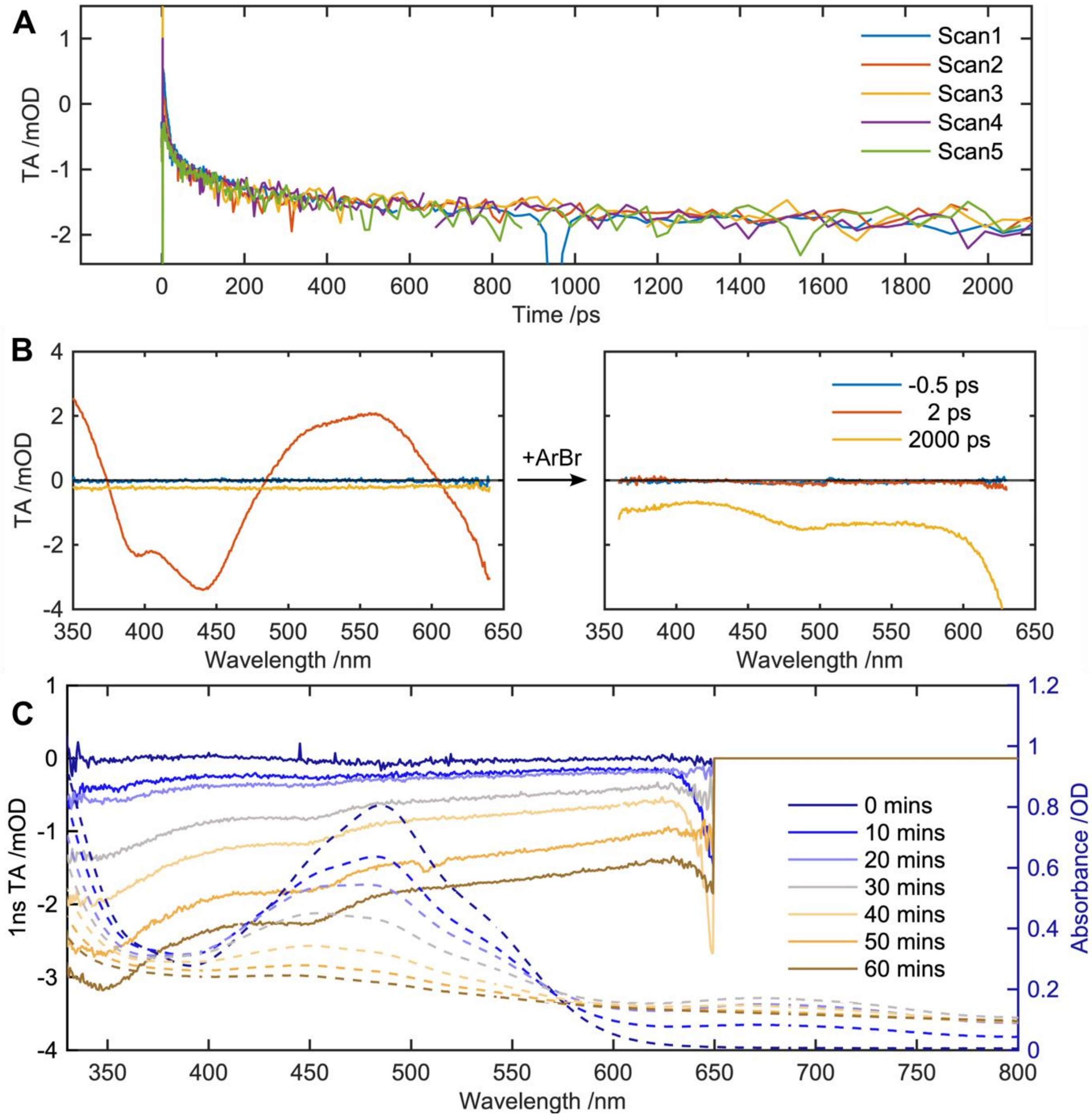

Figure S33. TA spectra for sample containing **3** under various conditions following 700 nm, 1 μJ photoexcitation. (A) Consecutive scans of the TA measurement showing the reproducibility of the long-lived feature. (B) Difference spectra before and after addition of aryl bromide showing disappearance of Ni(I) signal. (C) Difference spectra at 1 ns (solid lines) recorded at different irradiation times alongside the corresponding absorption spectra (dotted lines).

From comparison to Figure S18, it is clear that the shape of the long-lived feature varies between samples but is reproducible for multiple measurements of the same solution. The dependence of the long-lived TA signal on irradiation time is markedly different than that of the steady-state Ni(I) peak at 670 nm, further supporting the idea that this long-lived feature does not originate from **3**.

While we can conclude that the long-lived feature originates from neither photoexcited **3** nor its Ni(II) parent, its true identity is unknown. Previous studies into the speciation which occurs when Ni(II)–bpy aryl halide complexes are irradiated present three potential other candidates: Ni(II)/Ni(I) bimetallic aggregates,[6,16] Ni(I)/Ni(I) dimers,[6,17,18] or high-spin Ni(II)–bpy dihalide.[6,7] The first of these require there be significant Ni(II) parent in solution, which does not fit with the above kinetic observations. Most likely for our case is the insoluble (or sparingly soluble) Ni(I)/Ni(I) dimer which forms irreversibly and depends only on the Ni(I) concentration (formation of the dimer is expected to be the main thermal degradation pathway for **3**)[6] or further downstream decomposition resulting in the high-spin Ni(II)–bpy dihalide.

Therefore, we tentatively postulate that the pump pulse deposits a significant amount of thermal energy into the system causing dissolved dimers or high-spin Ni species to precipitate. The newly formed precipitate at the focus of the light would then slowly diffuse outwards, scattering a greater proportion of light as the delay time increases. This would be evidenced by a growing GSB in the TA signal, such as what we observe. Then, over the subsequent microseconds, the precipitate would diffuse far enough away from the focused light as to not affect the transmitted signal (or the precipitate could redissolve), thereby making the TA repeatable. Whatever the origin of the signal, it arises from neither photoexcited parent Ni(II) nor the Ni(I) complexes studied herein.

S1.6 Fits to Alternative Relaxation Models

In an attempt to rationalize the differences between **1-3** and **4-5**, we considered the possibility of alternative decay pathways, including relaxation through the intermediacy of an optically dark metal-centered state (^{2}d-d) or a ^{4}MLCT state accessed by intersystem crossing. Rate-limiting decay of a dark ^{2}d-d state can be excluded by considering that the relaxation proceeds through simultaneous recovery of the ESA and GSB with isosbestic points for all compounds and the presence of strong ESA in the visible. Therefore, if a dark ^{2}d-d state is formed, it must relax much faster than the ^{2}MLCT. The ^{2}d-d → ^{2}GS transition has an energy gap similar to that of the MLCT state and is formally orbitally forbidden, making it unlikely that it is significantly faster than a rate-limiting ^{2}MLCT → ^{2}d-d step (Figure S44). Conversely, optical TA is not sensitive to state multiplicity, allowing for the possibility of relaxation from ^{2}MLCT → ^{4}MLCT by fast intersystem crossing followed by spin-forbidden relaxation to the ground state (^{4}MLCT → ^{2}GS, Figure S42). Albeit not completely unprecedented, the observed values for τ_3 would constitute very short time constants for reverse intersystem crossing to the ground state.[5] We further find that the relaxation kinetics of this step show no dependence on pump wavelength (Table S3). As such, we propose that all compounds relax from the same MLCT excited state (Figure 5A).

Several models have been presented for intramolecular electron transfer (ET) over the past decades; in this section we discuss four of these to understand the data presented in main text Figure 5B. These models all rely on the same four approximations:

1. The series of compounds relax from the same MLCT excited state.
2. E_{MLCT} is an accurate reflection of the lowest MLCT state.
3. The electronic coupling is roughly the same along the series.
4. The series of compounds show roughly the same reorganization energies between ground and excited states.

That all compounds in the series relax from the same MLCT state is discussed above, and the association of this state to E_{MLCT} is supported by the TD-DFT assignments (Tables S5-S12) of the absorption spectra presented in Section S1.4. The lowest MLCT transition energy is only slightly lower than that of the lowest high-absorbance MLCT, justifying this approximation. The latter two are assumed for almost all literature examples of Marcus theory discussed below, and we see them as equally valid in this case. Only the bpy substituents or the halide change between compounds; the bpy coordination sites that have most influence on the MLCT character are mostly unchanged by these modifications. Therefore, we expect that the electronic coupling and reorganization energies are similar across all compounds. Any additional model-specific assumptions are discussed where relevant in the following paragraphs.

Weak Coupling Model

Jortner and co-workers[19,20] developed one such model to describe the non-radiative relaxation of excited states. Their framework relates the rate of relaxation to the deformation of the excited state relative to the ground state. If the deformation is relatively small – the so-called weak coupling limit – then the intersection between the ground- and excited-state potential energy surfaces (PESs) lies at very high energy. Therefore, relaxation proceeds through coupling of the excited state to high-energy vibronic modes of the ground state. At room temperature, this leads to Eq. (S3).

$$k = \frac{V^2}{\hbar}\sqrt{\frac{2\pi}{\hbar\omega_M \Delta G^\circ}} \exp\left(-\frac{\Delta G^\circ}{\hbar\omega_M}\left[\log\left(\frac{\Delta G^\circ}{d\lambda}\right) - 1\right]\right) \tag{S3}$$

Here $\hbar\omega_M$ is the energy of the highest vibrational mode with degeneracy, d. V is the matrix element for the transition, and ΔG° is the energy gap between the relaxed excited and ground states. Given the explicit dependence on the maximum vibrational frequency, the rate is expected to show a strong dependence on the character of this transition. To derive Eq. (S4), it is assumed that the reorganizational energy is small such that $\left[\log\left(\frac{\Delta G^\circ}{d\lambda}\right) - 1\right] > 0$. This results in a slightly stronger than exponential dependence on ΔG° and thus recovers the empirical energy gap law. Our main interest here lies with the dependence on ΔG° so we can simplify to

$$k = \frac{A}{\sqrt{\mathrm{E_{MLCT}} - \lambda}} \exp\left(-\frac{\mathrm{E_{MLCT}} - \lambda}{\hbar\omega_M}\left[\log\left(\frac{\mathrm{E_{MLCT}} - \lambda}{d\lambda}\right) - 1\right]\right), \tag{S4}$$

where the pre-factors have been condensed into A. The same assumption as in the main text that $\Delta G^o = \lambda - E_{MLCT}$ is also made to relate the model back to the experimentally accessible vertical transition energies. For all compounds, the highest vibrational modes are C–H stretches on the bpy at around 0.39 eV (Table S4); six of these are in common to all **1**–**5**. While not strictly degenerate, these are similar in energy so we follow literature precedent[19,21] in setting $d = 6$ and fit for the remaining parameters. This results in the fit shown in Figure 5B, with $\lambda = 0.09$ eV, $\hbar\omega_M = 0.41$ eV and $A = 0.26$ $\mathrm{eV}^{1/2}\,\mathrm{ps}^{-1}$. While it can describe the high-energy results well, the monotonically increasing function cannot account for the slower relaxation of the lower-energy compounds. Despite this, it does seem to recover the energy of the C–H stretches, suggesting that there may be some merit to the fit in the high energy region. Observation of a large change in rate upon deuteration of the bpy could interrogate this experimentally, as the energy change from C–H to C–D vibrational modes ($\hbar\omega_M$(C–D) ~ 0.28 eV)[19] are expected to result a sizable changes to the coupling between the ground and excited states.

Importantly, a globally poor fit to our data is to be expected from the weak coupling model given the assumptions taken in its derivation. Here we observe MLCT transitions where there will, by definition, be a significant redistribution of charge. Therefore, unlike a $\pi \rightarrow \pi^*$ transition for example, the reorganization energy here is relatively large, as is observed in the absorption spectra in Figure S10. Thus, again we find that our system most likely does not lie within the weak coupling limit.

Strong Coupling and Classical Marcus Models

When the excited state deformation is large enough, the intersection between the surfaces becomes thermally accessible. The system will instead move along the vibrational coordinate with a small activation energy into the ground-state PES through the intersection. This behavior is shown schematically in the main text Figure 5A and corresponds to the strong coupling limit presented by Jortner *et al.*[19] and given mathematically by main text Eq. (1). As ΔG° decreases, excited- and ground-state PESs transition from nested to displaced, and the resulting change in activation energy yields a parabolic dependence on ΔG°. In deriving Eq. (1), it is approximated that probability of surmounting the energy barrier is determined by the mean vibrational energy. Here we again relate back to E_{MLCT} to get Eq. (2) and then fit this to the observed rate constants. A good fit is achieved (Figure 5B) with fitting parameters: $\lambda = 0.77(3)$ eV, $\ln(A) = 25.7(6), \langle\hbar\omega\rangle = 0.13(7)$ eV. As discussed in the main text, $\langle\hbar\omega\rangle$ is indeed close to the calculated average

vibrational energy; the reorganizational energy is larger than that obtained from fitting the steady-state spectra, but of similar magnitude.

The most successful theory of electron transfer (ET) is Marcus theory.[22–24] However, this was developed for ET between two relatively independent species rather than the non-radiative relaxation we observe here.[25] These could be different molecules in the case of intermolecular ET or even a large molecule with well-separated donor and acceptor. This places limits on the applicability of the model to intramolecular systems. Despite this fact, we can still utilize Marcus theory to approximate the dynamics of charge transfer transitions. Making the same assumption that $\Delta G^{o} = \lambda - E_{MLCT}$ leads to Eq. (S5)

$$\ln(k) = \ln(A'') - \frac{(E_{\text{MLCT}} - 2\lambda)^2}{4\lambda k_B T}. \tag{S5}$$

Fitting this to the data in Figure 5B and fixing T to 298 K, yields $\lambda = 0.76(1)$ eV, $\ln(A) = 27.1(2)$. This fit is plotted alongside the other three models in Figure 5B, where it is clearly worse than that of Eq. (2) yet still captures the main features of the data; the reorganization energy is consistent between the two models. The worse fit is to be expected given that the ET in question is short-range and intramolecular, and there is one fewer fitted parameter. Likewise, the consistency in λ likely only reflects the very similar functional forms of the models.

Nonetheless, the loose applicability of classical Marcus theory here is borne out by the similarities between the Marcus equation and the strong coupling model discussed in the main text and Eq. (2). Both the classical Marcus theory and strong coupling models have the same form, save for the substitution $\langle \hbar\omega \rangle = 2k_BT$, which physically corresponds to the charge transfer being driven by vibrational modes rather than thermal energy. This difference follows from their derivation in classical and quantum regimes, respectively. Accordingly, the semiclassical derivation of Marcus theory leads to the same dependence on $\langle \hbar\omega \rangle$.[26]

Vibronic Marcus Model

Classical Marcus theory can be extended into a quantum regime by considering coupling of the electron transfer with vibronic modes.[27] Summation over all vibronic modes is not practical for fitting experimental data, so this can be simplified by assuming quantum coupling to just one mode – either one dominant mode, or a representative average mode – while low frequency modes are treated classically.[28] This semiclassical vibronic Marcus model was first applied in the context of long-distance intramolecular electron transfer[29] and splits the reorganization energy into contributions from the solvent and vibrations, λ_S and λ_V, respectively. Mathematically, this gives rate constants of

$$k = V^2 \sqrt{\frac{\pi}{\hbar^2 \lambda_S k_B T}} \sum_{w=0}^{\infty} \frac{e^S S^n}{n!} \exp\left(- \frac{(\Delta G^\circ + \lambda_\text{S} + n\hbar\omega)^2}{4\lambda_S k_B T} \right), \tag{S6}$$

where $S = \lambda_V/\hbar\omega$ and $\hbar\omega$ is the energy of the mode to which the ET couples. The sum over w accounts for transitions into each of the ground-state vibronic modes and the weighting of the exponential corresponds to the Franck-Condon factor for each transition. Substituting $\Delta G^{o} = \lambda - E_{MLCT} = (\lambda_S + \lambda_V) - E_{MLCT}$ results in

$$k = A' \sum_{n=0}^{\infty} \frac{e^S S^n}{n!} \exp\left(-\frac{(\mathrm{E_{MLCT}} - 2\lambda_\mathrm{S} - \lambda_V - n\hbar\omega)^2}{4\lambda_S k_B T}\right), \tag{S7}$$

where the pre-factors are again collated into A. This constitutes the fourth model we fit to our data, resulting in parameters $A = 0.044\ \mathrm{ps}^{-1}, \hbar\omega = 0.21\ \mathrm{eV}, \lambda_S = 0.54\ \mathrm{eV}, \lambda_V = 0.22\ \mathrm{eV}$; the fit to this vibronic Marcus model is plotted in Figure 5B. Accurate estimates of errors on these values could not be obtained due to the infinite sum requiring a more involved fit. Given the explicit dependence on solvent, we exclude the toluene datapoints from the fit here, but their inclusion has a negligible impact on the fitted parameters. The Franck-Condon weighted sum results in an asymmetric, near-parabolic dependence that gives the best fit to the data out of all models considered here (albeit with the greatest number of fitted parameters).

Previous studies on polypyridyl-Fe complexes[15] supposed that bpy breathing modes couple to MLCT transitions. Vibronic coupling to a particular mode is typically accompanied by corresponding vibronic progressions into the absorption spectrum. However, given the linewidth and number of transitions expected in the spectra for **1**–**5**, we are not able to say whether significant vibronic transitions are present. Despite this, the 0.21 eV value obtained from the fit does lie within the range of bpy breathing modes of **1**–**5**, which are predicted by DFT to be ~0.2 eV. It is also often assumed that ET along organic spacers is coupled to general skeletal modes in the region of 0.19 eV,[29–31] further corroborating the fitted value.

Only the solvent contribution to the reorganization energy affects the linewidths of the peaks in the absorption spectrum. Indeed, λ matches the widths obtained from Section S1.4. This value is also identical to that observed for Zn porphyrin complexes in THF.[30] The vibrational reorganization energy is much harder to determine experimentally, but it can be approximated through calculations of the potential energy surfaces in Figure S44. These calculations (with implicit solvation model, CPCM) do not account for reorganization of the solvent, reducing the predicted contribution of λ_S. Consequently, they should roughly correspond to λ_V and are found to be 0.18 eV and 0.33 in **1** and **5**, respectively, which is close to the 0.22 eV given by the fit. However, as discussed in Section S2.5, there are several limitations to DFT in these systems. Literature values for λ_V are found in the range 0.15–0.6 eV,[29–32] reflecting the dependence on excited-state distortion, which can vary greatly between compounds. The total reorganization energy $\lambda = \lambda_S + \lambda_V = 0.76$ eV found by the vibronic Marcus fit is consistent with the classical Marcus and strong coupling models.

Comparing Eqs. S6 and S7, $V = 1.4$ meV $\ll k_B T$, indicating that the relaxation is nonadiabatic, which is assumed by the Marcus model and implies that crossing the PES is the rate determining step.[29]

Despite being developed for longer-range ET, the vibronic Marcus model still appears to provide a self-consistent description of the data here. The model has also been applied with success to short-range intramolecular charge transfer transitions in both inorganic and organic systems, suggesting that its semiclassical nature is not a significant limitation.[15,30–35] Both strong coupling and vibronic Marcus models appear to give good descriptions of the data, and both of these arise from considering the intersection of displaced PESs.

Therefore, while Marcus theory was initially developed for long-distance charge transfer, it is functionally the same as strong coupling in this case. In compounds **1–3** with their larger E_{MLCT}, the ground-state and MLCT PESs are nested (known as “inverted” in the language of Marcus theory) and so behave in a manner reminiscent of the energy gap law. For **4–5**, which have smaller E_{MLCT}, the PES are displaced (normal) so the energy gap law behavior is effectively reversed.

S2. Computational Section.

S2.1. General Computational Details.

All the computations were performed using ORCA 5.0.3 software.[36,37] Molecular structures were optimized with density functional theory (DFT) using the BP86 functional[38,39] with the def2-TZVP basis set[40] on all atoms except Ni which received def2-TZVPP. The Weigend auxiliary basis set, def2/J was used.[41] The calculations were expedited by employing the resolution-of-identity (RI) approximation. The D3BJ dispersion correction[42,43] was applied; the conductor-like polarizable continuum (CPCM) solvation model[44,45] was used for implicit solvation.

Single point calculations with the hybrid B3LYP functional[46,47] and def2-TZVP+def2-TZVPP(Ni) basis set were used to refine the electronic energies and molecular properties. Again, def2/J was used as auxiliary basis, alongside D3BJ and CPCM for dispersion correction and solvation modeling, respectively. The chain-of-spheres approximation, RIJCOSX,[48] was utilized as is default for hybrid-DFT in ORCA 5.

For equilibrium geometries, the terms contributing to Gibbs free energy were calculated as follows:

$$G = E_{\mathrm{el}} + G_{\mathrm{solv}} + [E_{\mathrm{ZPVE}} + RT - RT \ln (Q)], \qquad \text{(S8)}$$

where, *i*) E_{el} is the *in vacuo* electronic energy; calculated using RI-B3LYP-D3 method as above, *ii*) G_{solv} is the free energy of solvation; calculated using CPCM, *iii*) $[E_{\mathrm{ZPVE}} + RT - RT \ln(Q)]$ corresponds to the thermal enthalpic and entropic contributions to the solute energy with E_{ZPVE} and Q being the zero-point vibrational energy and the molecular partition function, respectively; obtained from frequency calculations with the rigid rotor/harmonic oscillator approximation (for $p = 1$ bar, $T = 298$ K).

The spin states considered in our computational analysis of **1-5** were all doublets (S = 1/2), taken following experimental data. For **1** and **5**, intersystem crossing to a quartet (S = 3/2) was also considered.

On top of the DFT-optimized geometries, time-dependent density functional theory (TD-DFT) was used to predict the excited states and compare the computational absorption patterns with experimental UV-vis-NIR spectra. For each TD-DFT calculation, 100 roots were considered. Relaxed excited state geometries were also found using TD-DFT via the iRoot keyword in ORCA.

The XYZ coordinate system used for labeling orbitals throughout the manuscript was selected according to the parent Ni(II) complexes to maintain consistency with the previous studies,[4,6,49] i.e., the x and y axes are oriented along the Ni–N(bpy) axes, making the singly occupied orbital to be the $3d(x^2\text{-}y^2)$. By this, the orbitals parallel and perpendicular to the Ni–halide axis are the mixtures of d(xz) and d(yz) orbitals. To distinguish them, we thus label the orbitals as d(xz/yz,∥) and d(xz/yz,⊥) according to their parallel and perpendicular orientation to the Ni–halide axis.

S2.2. Sample ORCA Inputs.

Example DFT Geometry Optimization	Example DFT Single Point Calculation
! UKS BP86 def2-TZVP def2/J RI D3BJ<br>! TightSCF CPCM(THF) SlowConv<br>! OPT FREQ<br><br>%basis<br>newgto Ni "def2-TZVPP" end<br>end<br><br>*xyzfile 0 2 structure.xyz	! UKS B3LYP def2-TZVP def2/J RIJCOSX<br>! D3BJ CPCM(THF) SlowConv<br>! MOREAD<br>! SP<br><br>%moinp "optimization.gbw"<br><br>%basis<br>newgto Ni "def2-TZVPP" end<br>end<br><br>*xyzfile 0 2 optimized-structure.xyz
**Example TD-DFT Calculation**	**Example TD-DFT Excited State Optimization**
! UKS B3LYP def2-TZVP def2/J RIJCOSX<br>! D3BJ CPCM(THF) SlowConv<br><br>%basis<br>newgto Ni "def2-TZVPP" end<br>end<br><br>%tddft<br>nroots 100<br>maxdim 5<br>end<br><br>*xyzfile 0 2 optimized-structure.xyz	! UKS B3LYP def2-TZVP def2/J RIJCOSX<br>! D3BJ CPCM(THF) SlowConv<br>! OPT Keepdens<br><br>%basis<br>newgto Ni "def2-TZVPP" end<br>end<br><br>%tddft<br>nroots 5<br>maxdim 5<br>IRoot 1<br>end<br><br>* xyzfile 0 2 optimized-structure.xyz

S2.3. DFT Molecular Orbital Diagrams and Vibrational Energies

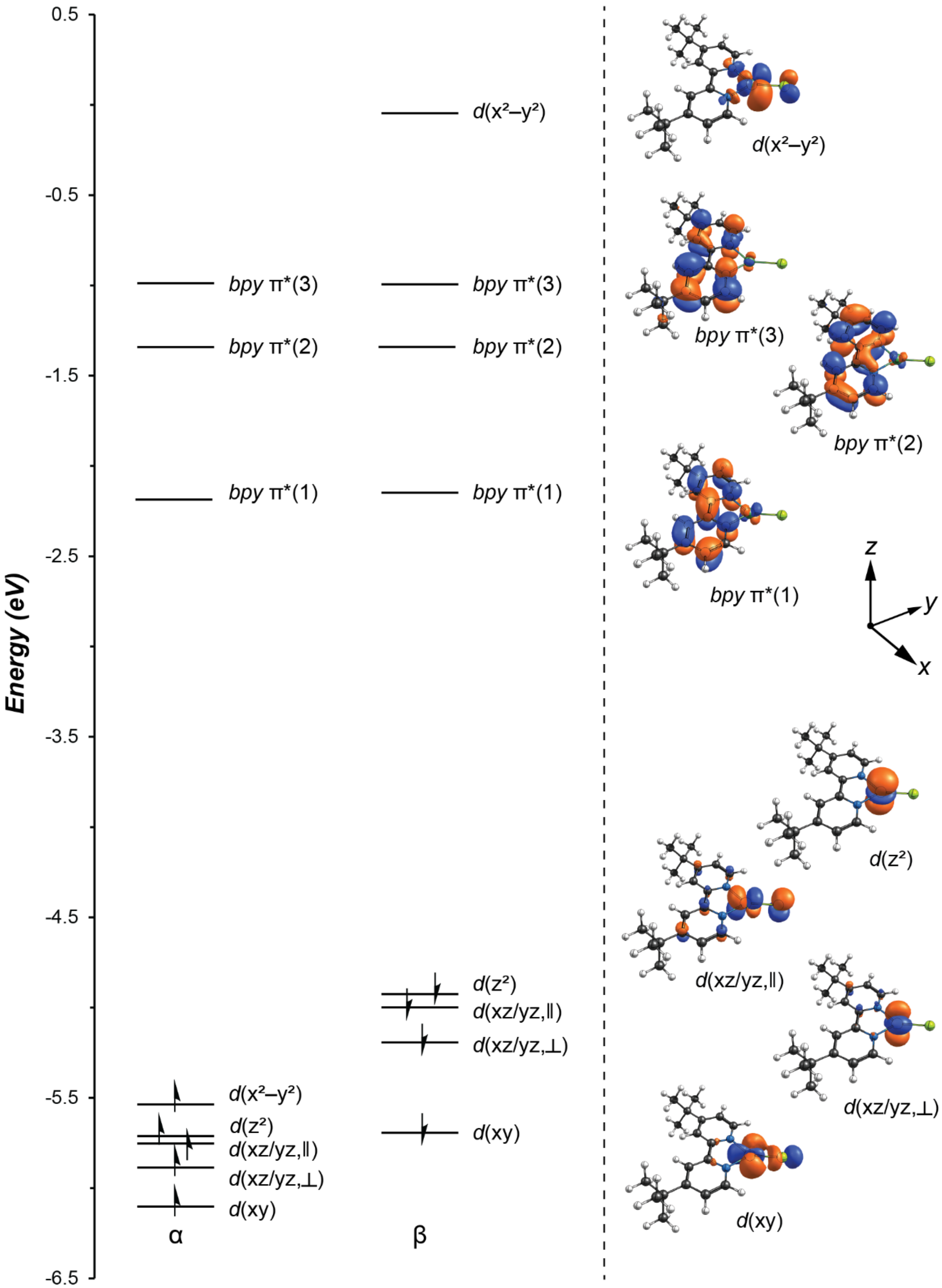

Figure S34. Molecular orbital diagram for **1** at the DFT(B3LYP) level.

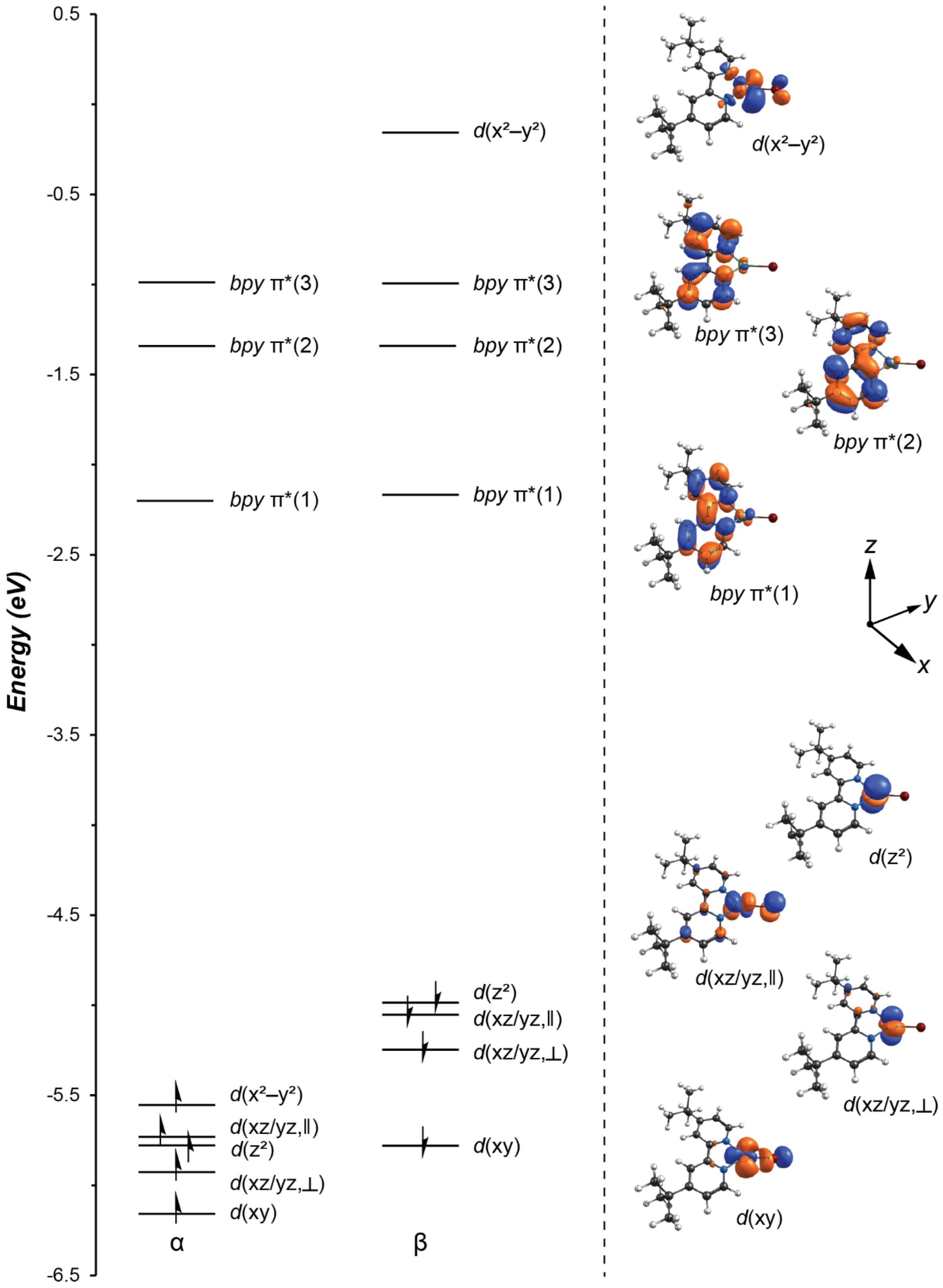

Figure S35. Molecular orbital diagram for **1-Br** at the DFT(B3LYP) level.

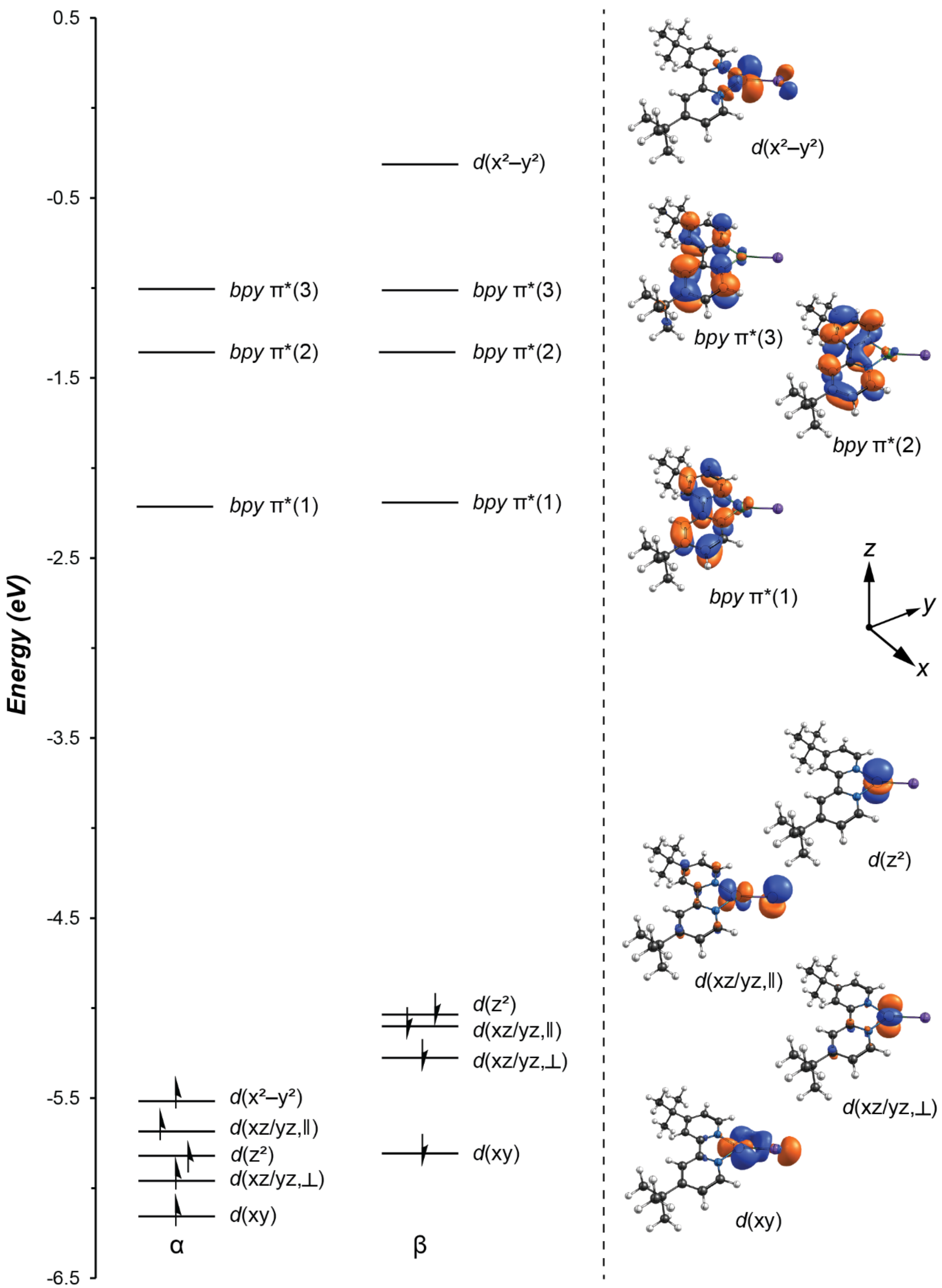

Figure S36. Molecular orbital diagram for **1-I** at the DFT(B3LYP) level.

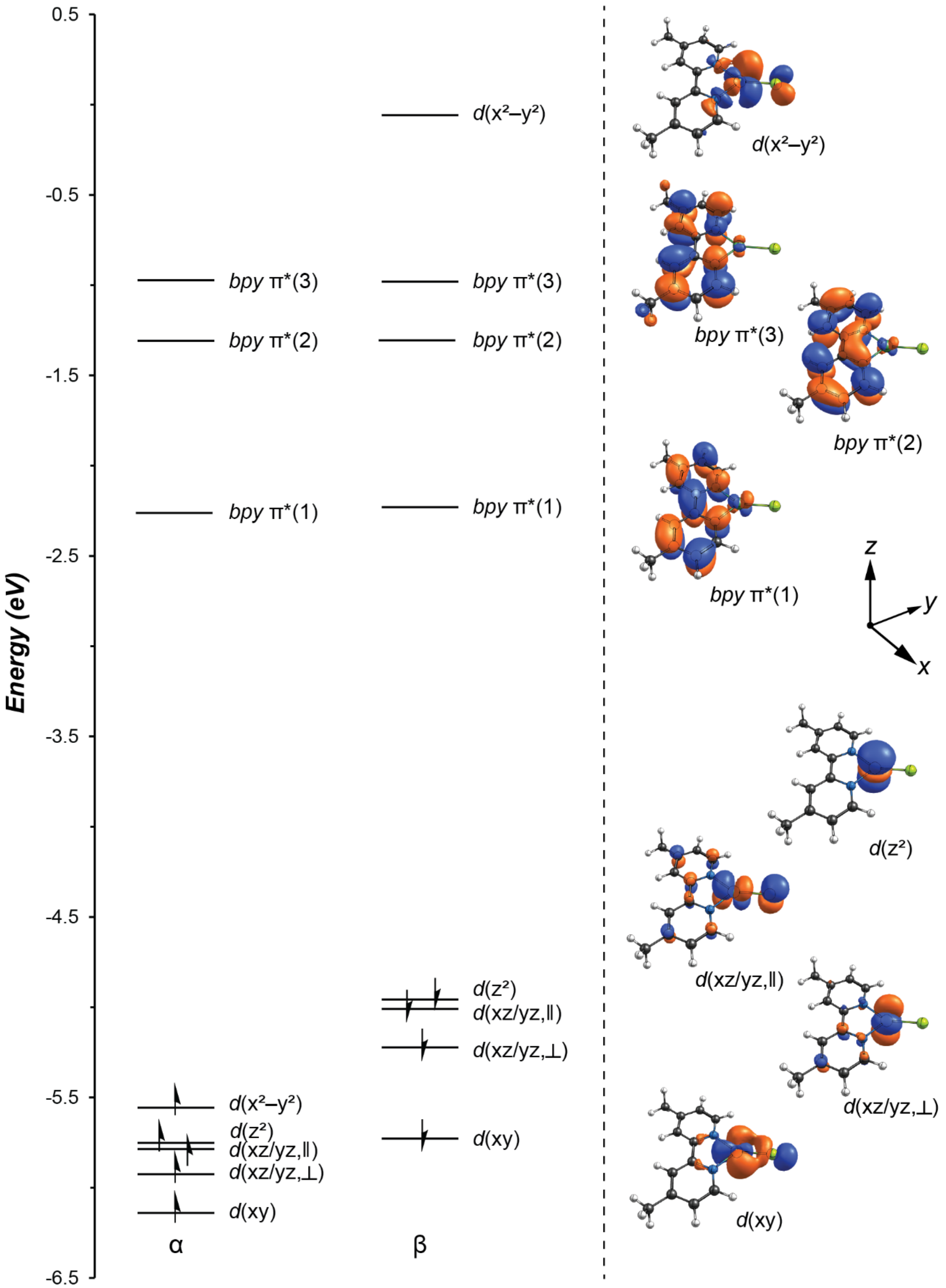

Figure S37. Molecular orbital diagram for **2** at the DFT(B3LYP) level.

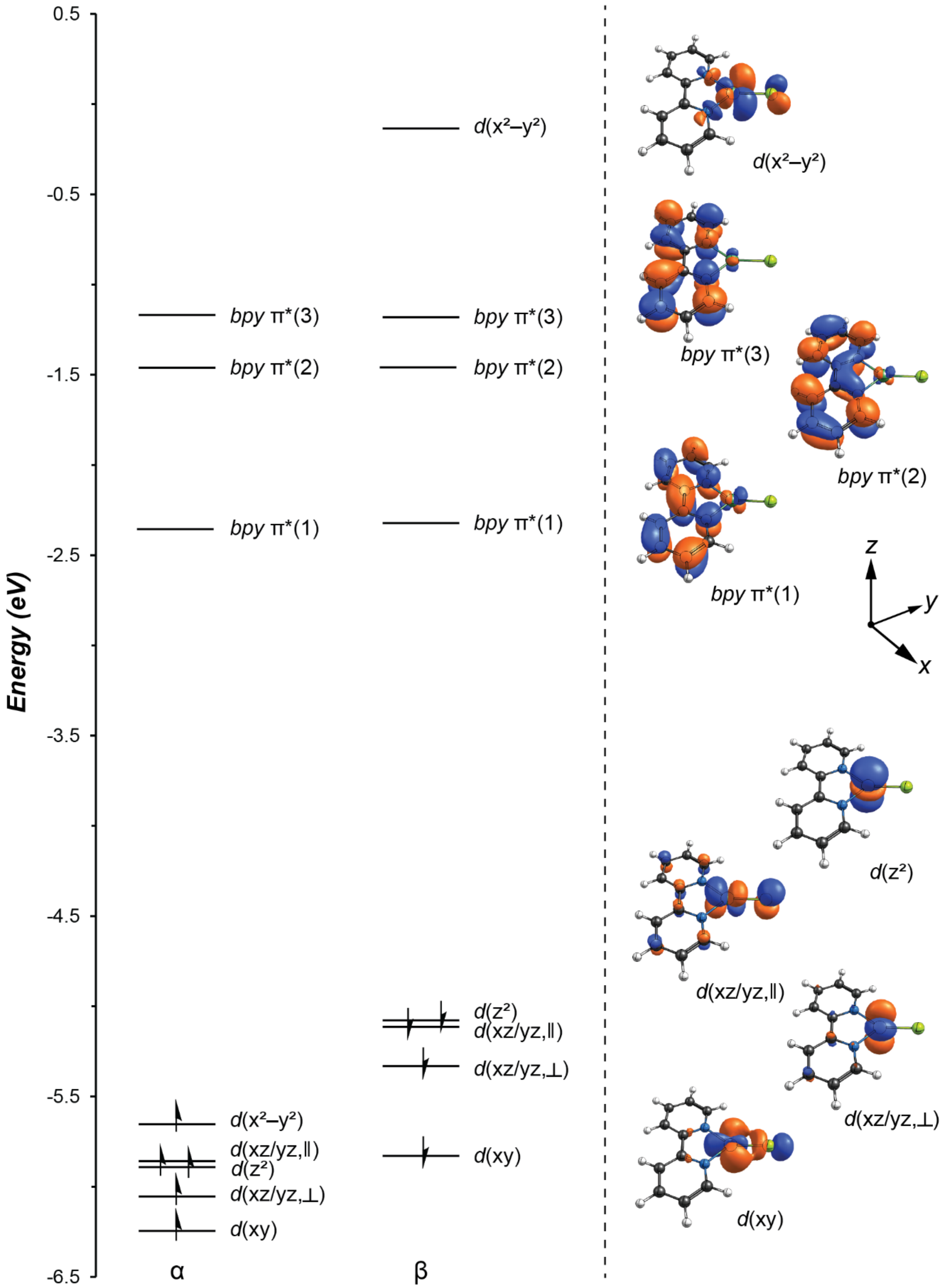

Figure S38. Molecular orbital diagram for **3** at the DFT(B3LYP) level.

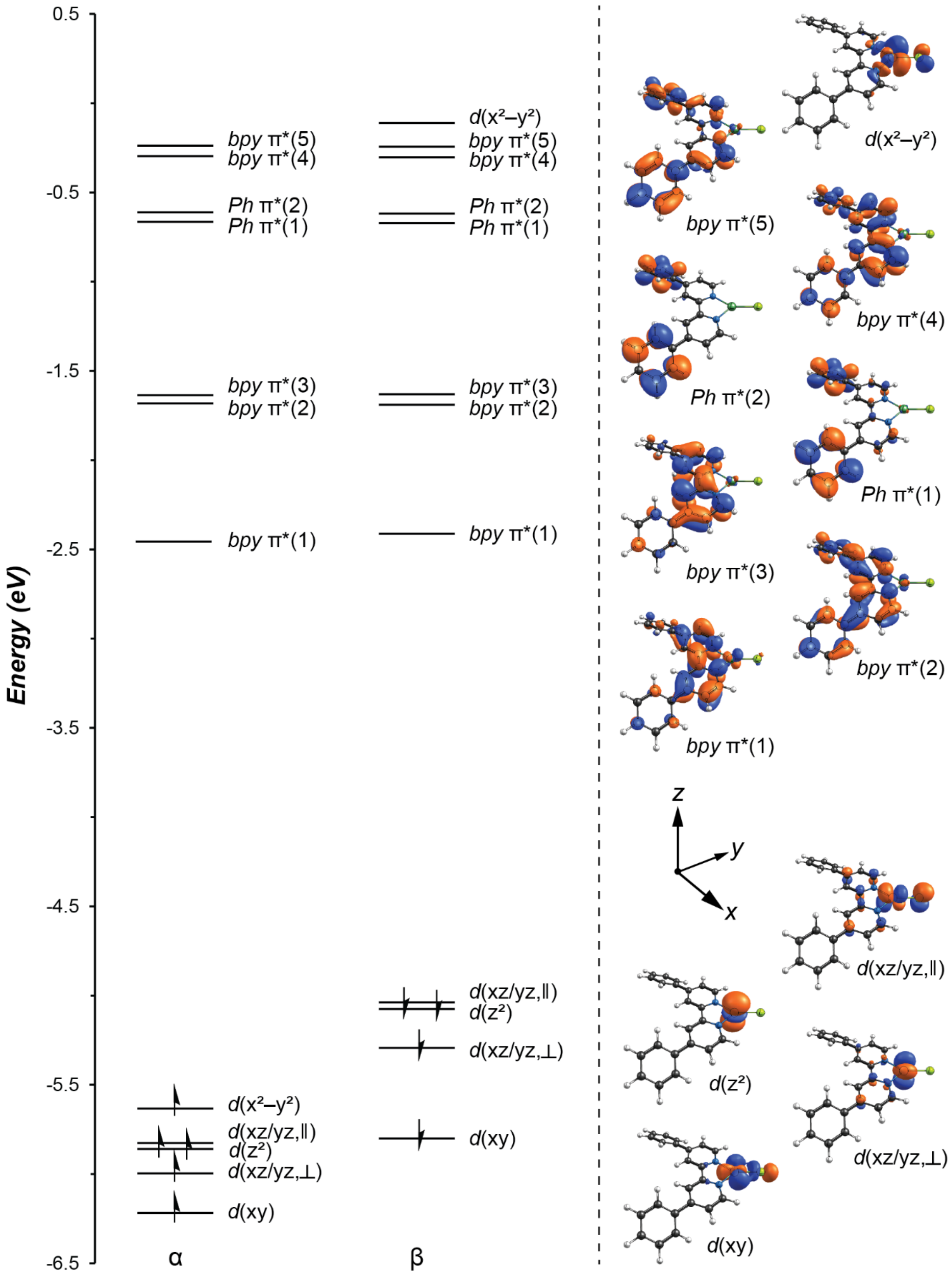

Figure S39. Molecular orbital diagram for **4** at the DFT(B3LYP) level.

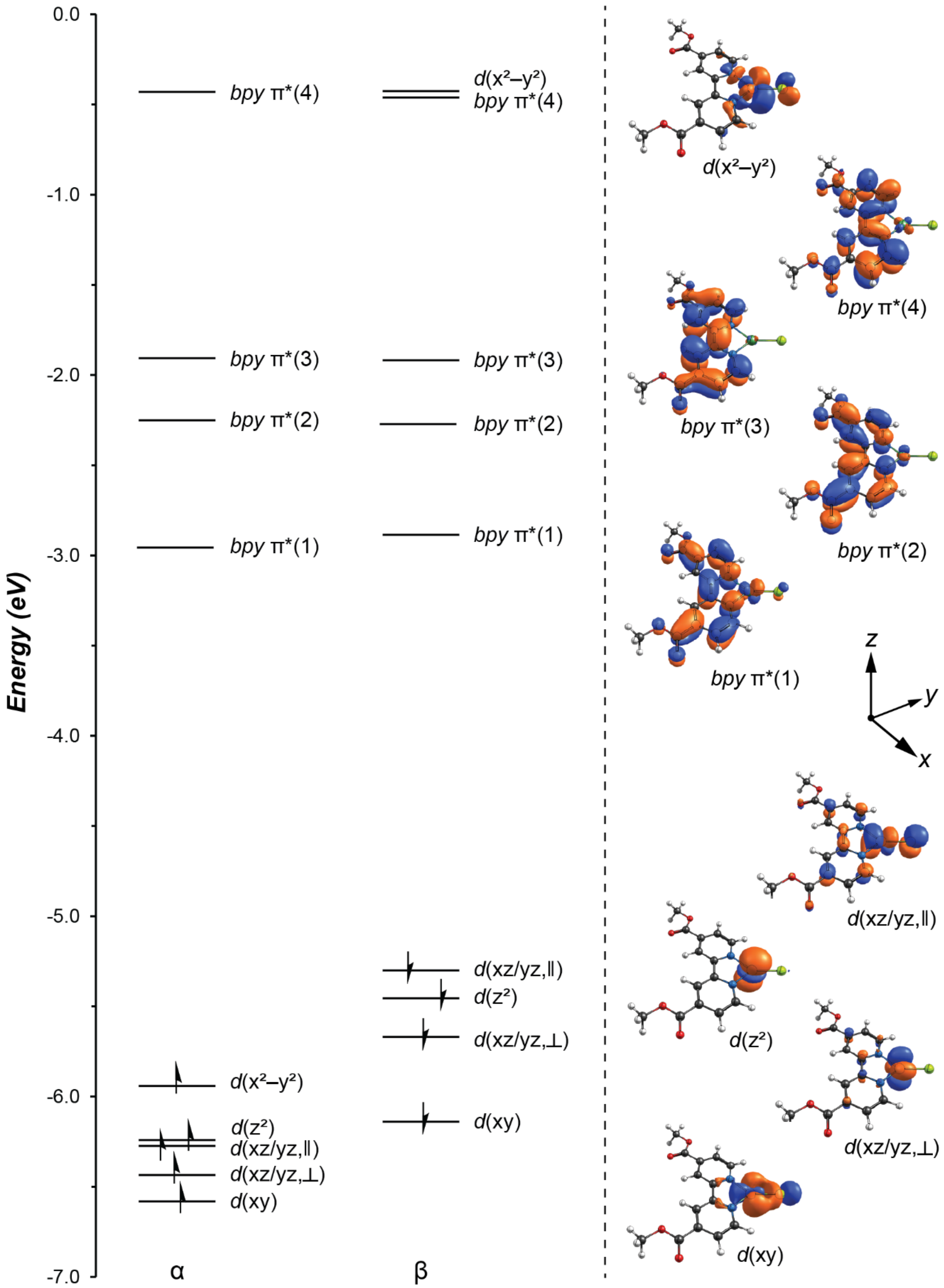

Figure S40. Molecular orbital diagram for **5** at the DFT(B3LYP) level.

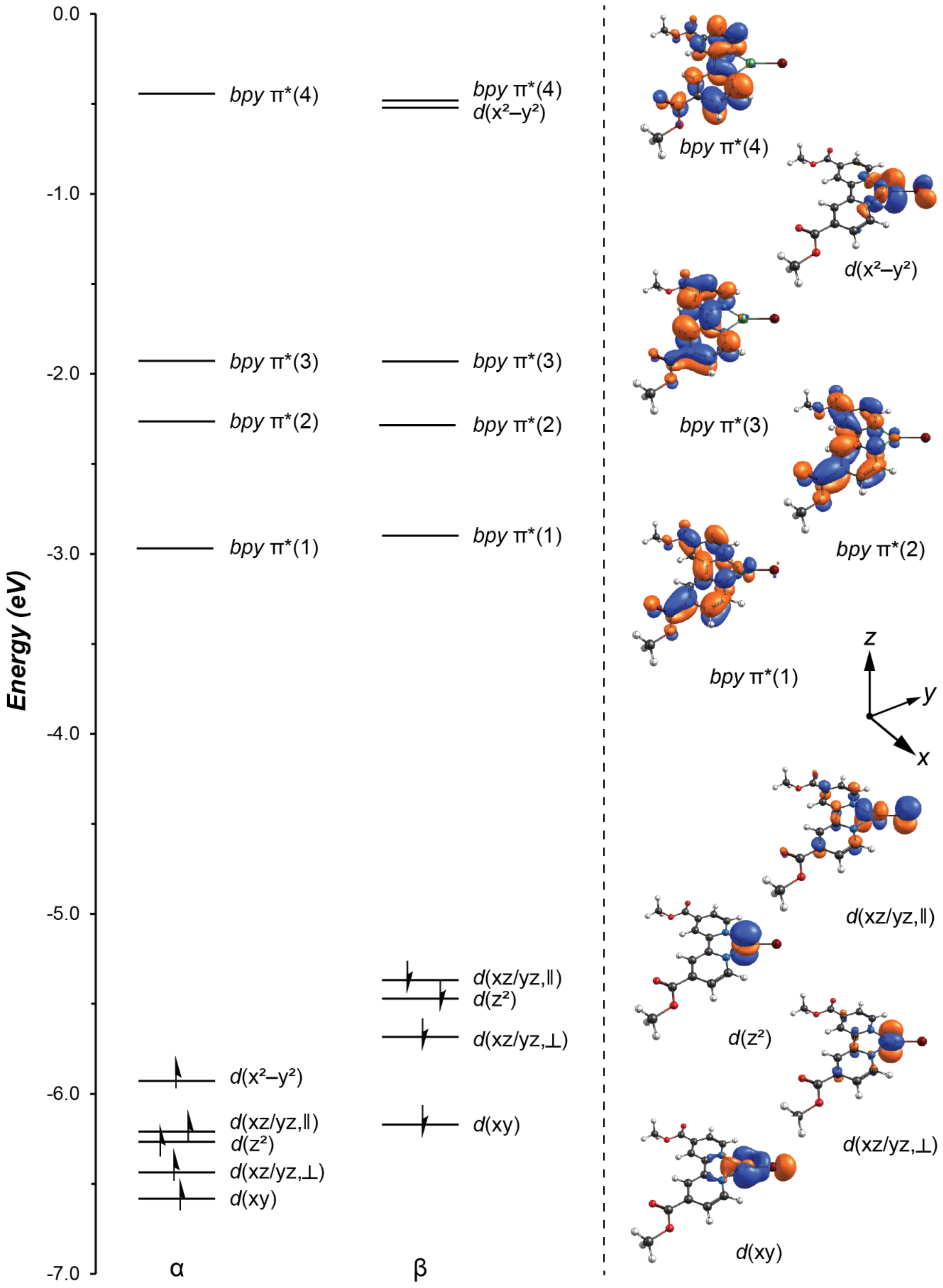

Figure S41. Molecular orbital diagram for **5-Br** at the DFT(B3LYP) level.

Table S4. Vibrational energies for complexes **1-5** computed at the DFT (BP86) level. The highest vibrational level for all the complexes is a C–H stretching frequency on the bipyridine ligand.

Compound	Average Vibrational Energy, $\langle \hbar\omega \rangle$ (cm^{-1} / eV)	Highest Vibrational Energy (cm^{-1} / eV)
**1**	1241.1 / 0.154	3145.3 / 0.390
**1-Br**	1240.0 / 0.154	3145.1 / 0.390
**1-I**	1239.9 / 0.154	3148.1 / 0.390
**2**	1175.9 / 0.146	3124.6 / 0.387
**3**	1142.8 / 0.142	3141.4 / 0.389
**4**	1145.1 / 0.142	3135.7 / 0.389
**5**	1087.4 / 0.135	3155.3 / 0.391
**5-Br**	1086.3 / 0.135	3155.4 / 0.391
**Average**	1169.8 / 0.145	3143.9 / 0.390

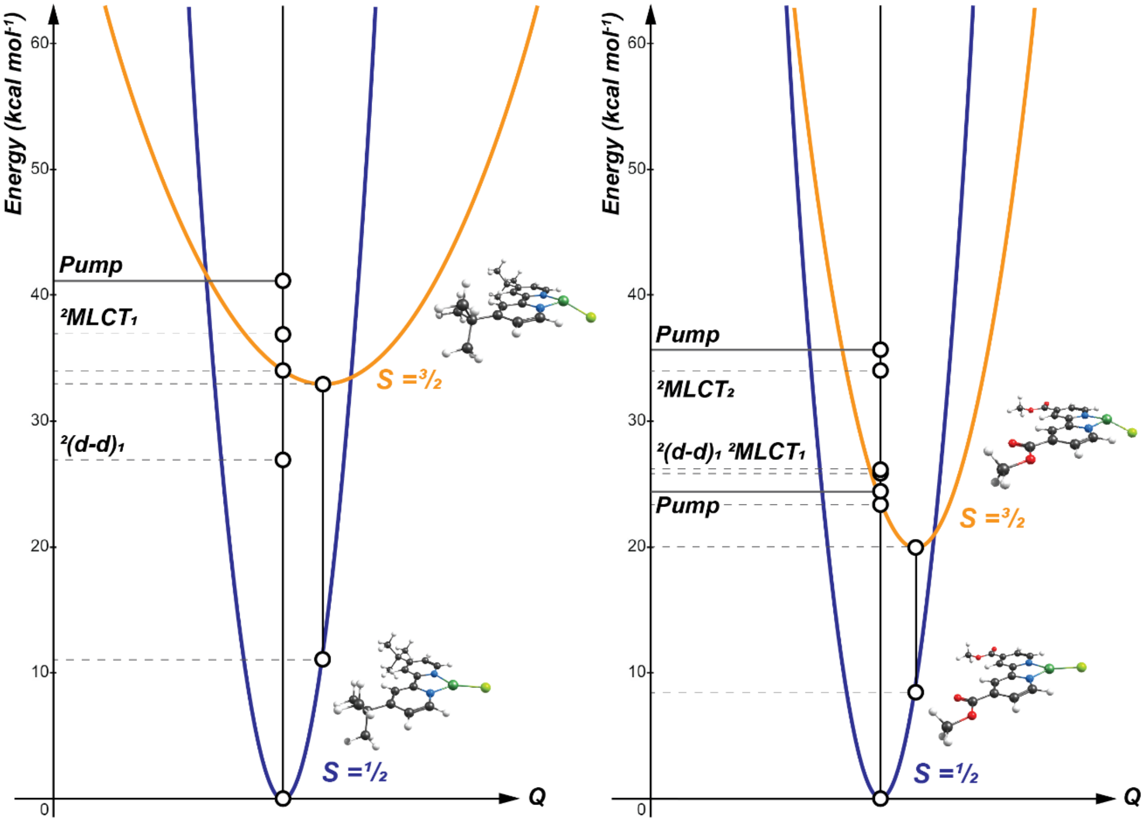

Figure S42. Computed potential energy surfaces for **1** (left) and **5** (right) showing the positions of the calculated TD-DFT transitions (see below) relative to the TA pump (800 or 1200 nm). Relaxed structures computed at the DFT BP86 level for the doublet ground state (blue surface) and relaxed quartet excited state (orange surface) are shown. Open circles indicate computed values and correspond to the energy markers on the y-axis. Calculations were performed to investigate the possibility of intersystem crossing to a quartet state (see Supporting Information Section 1.6; Fits to Alternative Relaxation Models for more details). Since both quartets are nested in the doublet surface (energy gap law behavior/Marcus inverted region), one would expect **5** to relax more quickly than **1** given its lower energy (a result opposite of that to the experiment). Thus, DFT suggests that a rate-limiting ^{4}MLCT → ^{2}GS is unlikely. We note, however, that accurate computed displacements are precluded without the use of an explicit solvation model to account for outer sphere reorganization energy. Vertical and relaxed excited state energies of **5** are likely to be overestimated (see Supporting Information Section 2.5; Limitations of DFT/TD-DFT for more details).

S2.4. TDDFT Spectra and Tabulated Transitions

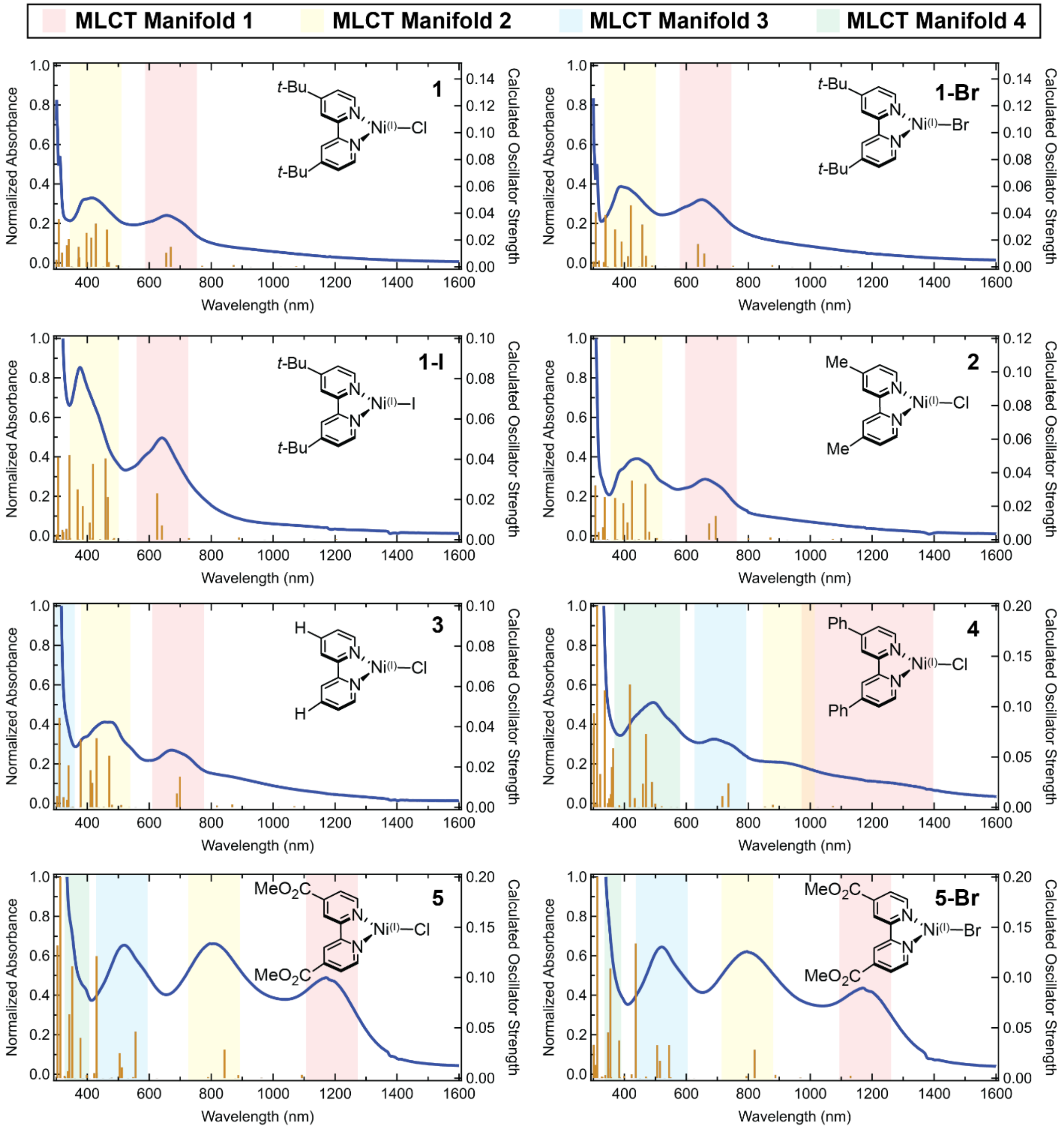

Figure S43. Experimental UV-vis-NIR spectra of complexes **1-5** (blue lines) and their predicted excited state transitions (orange sticks) using TDDFT (B3LYP) with the CPCM(THF) solvation model. MLCT Manifolds are labelled from lowest to highest energy and are denoted by the highlighted regions (Manifold 1 = magenta, 2 = yellow, 3 = cyan, 4 = green). Complexes **4**, **5**, and **5-Br** are not well modelled by TD-DFT (note the absence of the low energy MLCT Manifold 1).

Table S5. Absorption transitions for the equilibrium structure of **1** at the TDDFT(B3LYP) level with the CPCM(THF) solvation model.

State	*E* (cm^{-1})	*E* (nm)	f_{osc}	Transition Assignment		
1	9305	1075	0.0004300	β-Ni 3d(z^2)	→	β-Ni 3d(x^2-y^2)
2	9758	1025	0.0000523	β-Ni 3d(xz/yz,⊥)	→	β-Ni 3d(x^2-y^2)
3	10781	928	0.0000023	β-Ni 3d(xz/yz,∥)	→	β-Ni 3d(x^2-y^2)
4	11462	873	0.0014185	β-Ni 3d(xy)	→	β-Ni 3d(x^2-y^2)
5	12973	771	0.0008758	β-Ni 3d(z^2)	→	β-π*(1)
6	14931	670	0.0148499	β-Ni 3d(xz/yz,∥)	→	β-π*(1)
7	15269	655	0.0105068	β-Ni 3d(xz/yz,⊥)	→	β-π*(1)
8	18675	536	0.0000537	α-Ni 3d(x^2-y^2)	→	α-π*(1)
9	19490	513	0.0000902	β-Ni 3d(xy)	→	β-π*(1)
10	20225	494	0.0010854	α-Ni 3d(z^2)	→	α-π*(1)
11	21293	470	0.0035294	α-Ni 3d(xz/yz,⊥)	→	α-π*(1)
12	21575	464	0.0276969	α-Ni 3d(xz/yz,∥)	→	α-π*(1)
13	22119	452	0.0002490	β-Ni 3d(z^2)	→	β-π*(2)
14	23376	428	0.0321762	β-Ni 3d(xz/yz,∥)	→	β-π*(2)
15	23483	426	0.0000779	α-Ni 3d(xy)	→	α-π*(1)
16	24201	413	0.0217040	β-Ni 3d(xz/yz,⊥)	→	β-π*(2)
17	24448	409	0.0000061	β-Ni 3d(z^2)	→	β-π*(3)
18	25159	398	0.0253318	β-Ni 3d(xz/yz,∥)	→	β-π*(3)
19	26720	374	0.0069969	α/β-π	→	α/β-π*(1)
20	26879	372	0.0148811	β-Ni 3d(xz/yz,⊥)	→	β-π*(2)
21	26899	372	0.0060159	α-Ni 3d(x^2-y^2)	→	α-π*(2)
22	28373	352	0.0000002	β-Ni 3d(xy)	→	β-π*(2)
23	28664	349	0.0002781	α-Ni 3d(z^2)	→	α-π*(2)
24	29219	342	0.0000696	α-Ni 3d(x^2-y^2)	→	α-π*(3)
25	29386	340	0.0208177	α-Ni 3d(xz/yz,∥)	→	α-π*(2)
26	29945	334	0.0160776	α-Ni 3d(xz/yz,⊥)	→	α-π*(2)
27	30712	326	0.0000008	β-Ni 3d(xy)	→	β-π*(3)
28	31189	321	0.0000156	α-Ni 3d(z^2)	→	α-π*(3)
29	31378	319	0.0105837	α-Ni 3d(xz/yz,∥)	→	α-π*(3)
30	31867	314	0.0000012	α-Ni 3d(xy)	→	α-π*(2)
31	32464	308	0.0356698	α-Ni 3d(xz/yz,⊥)	→	α-π*(3)
32	33280	301	0.0052250	α/β-π	→	α/β-π*(2)

Table S6. Absorption transitions for the equilibrium structure of **1-Br** at the TDDFT(B3LYP) level with the CPCM(THF) solvation model.

State	E (cm^{-1})	E (nm)	f_{osc}	Transition Assignment		
1	8910	1122	0.0003991	β-Ni 3d(z²)	→	β-Ni 3d(x²-y²)
2	9364	1068	0.0000572	β-Ni 3d(xz/yz,⊥)	→	β-Ni 3d(x²-y²)
3	10612	942	0.0000099	β-Ni 3d(xz/yz,∥)	→	β-Ni 3d(x²-y²)
4	11396	878	0.0012884	β-Ni 3d(xy)	→	β-Ni 3d(x²-y²)
5	13312	751	0.0008388	β-Ni 3d(z²)	→	β-π*(1)
6	15204	658	0.0098469	β-Ni 3d(xz/yz,⊥)	→	β-π*(1)
7	15677	638	0.0169887	β-Ni 3d(xz/yz,∥)	→	β-π*(1)
8	18794	532	0.0001154	α-Ni 3d(x²-y²)	→	α-π*(1)
9	20078	498	0.0002039	β-Ni 3d(xy)	→	β-π*(1)
10	20432	489	0.0009542	α-Ni 3d(z²)	→	α-π*(1)
11	21288	470	0.0082070	α-Ni 3d(xz/yz,⊥)	→	α-π*(1)
12	21841	458	0.0316036	α-Ni 3d(xz/yz,∥)	→	α-π*(1)
13	22335	448	0.0002508	β-Ni 3d(z²)	→	β-π*(2)
14	23755	421	0.0457485	β-Ni 3d(xz/yz,∥)	→	β-π*(2)
15	23832	420	0.0000714	α-Ni 3d(xy)	→	α-π*(1)
16	24292	412	0.0079633	β-Ni 3d(xz/yz,⊥)	→	β-π*(2)
17	24722	405	0.0000136	β-Ni 3d(z²)	→	β-π*(3)
18	25572	391	0.0187942	β-Ni 3d(xz/yz,∥)	→	β-π*(3)
19	26754	374	0.0000144	α/β-π	→	α/β-π*(1)
20	26952	371	0.0017301	α-Ni 3d(x²-y²)	→	α-π*(2)
21	27024	370	0.0279220	β-Ni 3d(xz/yz,⊥)	→	β-π*(2)
22	28843	347	0.0000016	β-Ni 3d(xy)	→	β-π*(2)
23	28893	346	0.0002863	α-Ni 3d(z²)	→	α-π*(2)
24	29363	341	0.0002532	α-Ni 3d(x²-y²)	→	α-π*(3)
25	29497	339	0.0386636	α-Ni 3d(xz/yz,∥)	→	α-π*(2)
26	29991	333	0.0036054	α-Ni 3d(xz/yz,⊥)	→	α-π*(2)
27	31259	320	0.0000037	β-Ni 3d(xy)	→	β-π*(3)
28	31360	319	0.0000669	α-Ni 3d(z²)	→	α-π*(3)
29	31468	318	0.0047503	α-Ni 3d(xz/yz,∥)	→	α-π*(3)
30	32167	311	0.0000001	α-Ni 3d(xy)	→	α-π*(2)
31	32539	307	0.0407090	α-Ni 3d(xz/yz,⊥)	→	α-π*(3)
32	33281	301	0.0040320	α/β-π	→	α/β-π*(2)

Table S7. Absorption transitions for the equilibrium structure of **1-I** at the TDDFT(B3LYP) level with the CPCM(THF) solvation model.

State	E (cm^{-1})	E (nm)	f_{osc}	Transition Assignment		
1	8308	1204	0.0003008	β-Ni 3d(z²)	→	β-Ni 3d(x²-y²)
2	8829	1133	0.0000591	β-Ni 3d(xz/yz,⊥)	→	β-Ni 3d(x²-y²)
3	10281	973	0.0000098	β-Ni 3d(xz/yz,∥)	→	β-Ni 3d(x²-y²)
4	11238	890	0.0011694	β-Ni 3d(xy)	→	β-Ni 3d(x²-y²)
5	13731	728	0.0007837	β-Ni 3d(z²)	→	β-π*(1)
6	15603	641	0.0071086	β-Ni 3d(xz/yz,⊥)	→	β-π*(1)
7	15983	626	0.0229720	β-Ni 3d(xz/yz,∥)	→	β-π*(1)
8	18840	531	0.0001058	α-Ni 3d(x²-y²)	→	α-π*(1)
9	20550	487	0.0008454	β-Ni 3d(xy)	→	β-π*(1)
10	20661	484	0.0003566	α-Ni 3d(z²)	→	α-π*(1)
11	21430	467	0.0212783	α-Ni 3d(xz/yz,⊥)	→	α-π*(1)
12	21779	459	0.0404195	α-Ni 3d(xz/yz,∥)	→	α-π*(1)
13	22648	442	0.0002552	β-Ni 3d(z²)	→	β-π*(2)
14	23905	418	0.0376620	β-Ni 3d(xz/yz,∥)	→	β-π*(2)
15	23985	417	0.0008700	α-Ni 3d(xy)	→	α-π*(1)
16	24509	408	0.0085618	β-Ni 3d(xz/yz,⊥)	→	β-π*(2)
17	25028	400	0.0000163	β-Ni 3d(z²)	→	β-π*(3)
18	25953	385	0.0167115	β-Ni 3d(xz/yz,∥)	→	β-π*(3)
19	26754	374	0.0001335	α/β-π	→	α/β-π*(1)
20	26919	372	0.0001459	α-Ni 3d(x²-y²)	→	α-π*(2)
21	27132	369	0.0250047	β-Ni 3d(xz/yz,⊥)	→	β-π*(2)
22	29026	345	0.0008326	α-Ni 3d(z^2)	→	α-π*(2)
23	29179	343	0.0007470	β-Ni 3d(xy)	→	β-π*(2)
24	29219	342	0.0420723	α-Ni 3d(xz/yz,∥)	→	α-π*(2)
25	29398	340	0.0003774	α-Ni 3d(x²-y²)	→	α-π*(3)
26	29821	335	0.0000016	β-Br p(x/y,⊥)	→	β-π*(1)
27	30087	332	0.0053483	α-Ni 3d(xz/yz,⊥)	→	α-π*(2)
28	31007	323	0.0038798	β-Br 3p(z)	→	β-π*(1)
29	31270	320	0.0049428	β-Ni 3d(xz/yz,∥)	→	β-π*(3)
30	31478	318	0.0000444	α-Ni 3d(z^2)	→	α-π*(3)
31	31624	316	0.0000028	β-Ni 3d(xy)	→	α-π*(3)
32	31796	315	0.0000026	α-Br 3p(x/y,⊥)	→	α-π*(1)
33	32222	310	0.0000639	α-Br 3p(x/y,∥)	→	α-π*(2)
34	32239	310	0.0005966	α-Br 3p(z)	→	α-π*(1)
35	32707	306	0.0410170	α-Ni 3d(xz/yz,⊥)	→	α-π*(3)
36	33242	301	0.0026747	α/β-π	→	α/β-π*(2)

Table S8. Absorption transitions for the equilibrium structure of **2** at the TDDFT(B3LYP) level with the CPCM(THF) solvation model.

State	*E* (cm^{-1})	*E* (nm)	f_{osc}	Transition Assignment		
1	9316	1073	0.0004425	β-Ni 3d(z^2)	→	β-Ni 3d(x^2-y^2)
2	9715	1029	0.0000437	β-Ni 3d(xz/yz,⊥)	→	β-Ni 3d(x^2-y^2)
3	10799	926	0.0000010	β-Ni 3d(xz/yz,∥)	→	β-Ni 3d(x^2-y^2)
4	11474	872	0.0013918	β-Ni 3d(xy)	→	β-Ni 3d(x^2-y^2)
5	12493	801	0.0008308	β-Ni 3d(z^2)	→	β-π*(1)
6	14396	695	0.0141462	β-Ni 3d(xz/yz,∥)	→	β-π*(1)
7	14840	674	0.0096912	β-Ni 3d(xz/yz,⊥)	→	β-π*(1)
8	18193	550	0.0000616	α-Ni 3d(x^2-y^2)	→	α-π*(1)
9	19026	526	0.0000801	β-Ni 3d(xy)	→	β-π*(1)
10	19810	505	0.0010316	α-Ni 3d(z^2)	→	α-π*(1)
11	20808	481	0.0047675	α-Ni 3d(xz/yz,⊥)	→	α-π*(1)
12	21373	468	0.0333436	α-Ni 3d(xz/yz,∥)	→	α-π*(1)
13	22397	447	0.0002836	β-Ni 3d(z^2)	→	β-π*(2)
14	23062	434	0.0000109	α-Ni 3d(xy)	→	α-π*(1)
15	23560	425	0.0351858	β-Ni 3d(xz/yz,∥)	→	β-π*(2)
16	24395	410	0.0103361	β-Ni 3d(xz/yz,⊥)	→	β-π*(2)
17	24653	406	0.0000010	β-Ni 3d(z^2)	→	β-π*(3)
18	25222	397	0.0217931	β-Ni 3d(xz/yz,∥)	→	β-π*(3)
19	26536	377	0.0002506	α/β-π	→	α/β-π*(1)
20	27022	370	0.0248685	β-Ni 3d(xz/yz,⊥)	→	β-π*(3)
21	27136	369	0.0000233	α-Ni 3d(x^2-y^2)	→	α-π*(2)
22	28672	349	0.0000001	β-Ni 3d(xy)	→	β-π*(2)
23	28956	345	0.0003385	α-Ni 3d(z^2)	→	α-π*(2)
24	29420	340	0.0000264	α-Ni 3d(x^2-y^2)	→	α-π*(3)
25	29714	337	0.0254983	α-Ni 3d(xz/yz,∥)	→	α-π*(2)
26	30185	331	0.0074432	α-Ni 3d(xz/yz,⊥)	→	α-π*(2)
27	30928	323	0.0000002	β-Ni 3d(xy)	→	β-π*(3)
28	31403	318	0.0000074	α-Ni 3d(z^2)	→	α-π*(3)
29	31574	317	0.0046334	α-Ni 3d(xz/yz,∥)	→	α-π*(3)
30	32170	311	0.0000011	α-Ni 3d(xy)	→	α-π*(2)
31	32618	307	0.0323619	α-Ni 3d(xz/yz,⊥)	→	α-π*(3)

Table S9. Absorption transitions for the equilibrium structure of **3** at the TDDFT(B3LYP) level with the CPCM(THF) solvation model.

State	*E* (cm^{-1})	*E* (nm)	f_{osc}	Transition Assignment		
1	9360	1068	0.0005099	β-Ni 3d(z^2)	→	β-Ni 3d(x^2-y^2)
2	9822	1018	0.0000331	β-Ni 3d(xz/yz,⊥)	→	β-Ni 3d(x^2-y^2)
3	11017	908	0.0000012	β-Ni 3d(xz/yz,∥)	→	β-Ni 3d(x^2-y^2)
4	11518	868	0.0014098	β-Ni 3d(xy)	→	β-Ni 3d(x^2-y^2)
5	12214	819	0.0008277	β-Ni 3d(z^2)	→	β-π*(1)
6	14304	699	0.0152190	β-Ni 3d(xz/yz, ∥)	→	β-π*(1)
7	14507	689	0.0068829	β-Ni 3d(xz/yz,⊥)	→	β-π*(1)
8	17984	556	0.0000108	α-Ni 3d(x^2-y^2)	→	α-π*(1)
9	18676	536	0.0000766	β-Ni 3d(xy)	→	β-π*(1)
10	19629	510	0.0011378	α-Ni 3d(xz/yz,∥)	→	α-π*(1)
11	20849	480	0.0012266	α-Ni 3d(xz/yz,⊥)	→	α-π*(1)
12	21240	471	0.0256761	α-Ni 3d(z^2)	→	α-π*(1)
13	22078	453	0.0001954	β-Ni 3d(z^2)	→	β-π*(2)
14	22873	437	0.0000108	α-Ni 3d(xy)	→	α-π*(1)
15	23254	430	0.0342562	β-Ni 3d(xz/yz,∥)	→	β-π*(2)
16	23809	420	0.0000003	β-Ni 3d(z^2)	→	β-π*(3)
17	24018	416	0.0120753	β-Ni 3d(xz/yz,⊥)	→	β-π*(2)
18	24352	411	0.0183904	β-Ni 3d(xz/yz,∥)	→	β-π*(3)
19	26411	379	0.0336622	β-Ni 3d(xz/yz,⊥)	→	β-π*(3)
20	26464	378	0.0008086	α/β-π	→	α/β-π*(1)
21	26658	375	0.0000053	α-Ni 3d(x^2-y^2)	→	α-π*(2)
22	28207	355	0.0000234	β-Ni 3d(xy)	→	β-π*(2)
23	28322	353	0.0002583	α-Ni 3d(x^2-y^2)	→	α-π*(3)
24	28756	348	0.0000260	α-Ni 3d(xz/yz,∥)	→	α-π*(2)
25	29489	339	0.0208373	α-Ni 3d(z^2)	→	α-π*(2)
26	29918	334	0.0036422	α-Ni 3d(xz/yz,⊥)	→	α-π*(2)
27	29965	334	0.0000001	β-Ni 3d(xy)	→	β-π*(3)
28	30568	327	0.0000013	α-Ni 3d(xz/yz,∥)	→	α-π*(3)
29	30928	323	0.0049812	α-Ni 3d(z^2)	→	α-π*(3)
30	31742	315	0.0000018	α-Ni 3d(xy)	→	α-π*(2)
31	32255	310	0.0443949	α-Ni 3d(xz/yz,⊥)	→	α-π*(3)
32	33020	303	0.0055649	α/β-π	→	α/β-π*(2)

Table S10. Absorption transitions for the equilibrium structure of **4** at the TDDFT(B3LYP) level with the CPCM(THF) solvation model.

State	*E* (cm^{-1})	*E* (nm)	f_{osc}	Transition Assignment		
1	9315	1074	0.0012938	β-Ni 3d(z²)	→	β-Ni 3d(x²-y²)
2	9757	1025	0.0000222	β-Ni 3d(xz/yz,⊥)	→	β-Ni 3d(x²-y²)
3	10904	917	0.0000046	β-Ni 3d(xz/yz,∥)	→	β-Ni 3d(x²-y²)
4	11368	880	0.0023779	β-Ni 3d(xy)	→	β-Ni 3d(x²-y²)
5	11712	854	0.0007462	β-Ni 3d(z²)	→	β-π*(1)
6	13592	736	0.0236330	β-Ni 3d(xz/yz, ∥)	→	β-π*(1)
7	13959	716	0.0110160	β-Ni 3d(xz/yz,⊥)	→	β-π*(1)
8	17543	570	0.0000432	α-Ni 3d(x²-y²)	→	α-π*(1)
9	18154	551	0.0000570	β-Ni 3d(xy)	→	β-π*(1)
10	19220	520	0.0010219	α-Ni 3d(z²)	→	α-π*(1)
11	20014	500	0.0036455	α-Ni 3d(xz/yz,⊥)	→	α-π*(1)
12	20455	489	0.0250978	α-Ni 3d(xz/yz,∥)	→	α-π*(1)
13	20888	479	0.0000001	β-Ni 3d(z²)	→	β-π*(2)
14	21296	470	0.0728130	β-Ni 3d(xz/yz,∥)	→	β-π*(2)
15	21315	469	0.0001460	β-Ni 3d(z²)	→	β-π*(3)
16	21742	460	0.0233042	β-Ni 3d(xz/yz,∥)	→	β-π*(3)
17	22418	446	0.0000110	α-Ni 3d(xy)	→	α-π*(1)
18	23073	433	0.0091206	β-Ni 3d(xz/yz,⊥)	→	β-π*(3)
19	23927	418	0.1216611	β-Ni 3d(xz/yz,⊥)	→	β-π*(2)
20	25614	390	0.0002358	α-Ni 3d(x²-y²)	→	α-π*(2)
21	25808	388	0.0000905	α-Ni 3d(x²-y²)	→	α-π*(3)
22	26063	384	0.0019861	α/β-π(1)	→	α/β-π*(1)
23	27039	370	0.0000042	β-Ni 3d(xy)	→	β-π*(2)
24	27418	365	0.0000243	β-Ni 3d(xy)	→	β-π*(3)
25	27492	364	0.0585519	α-Ni 3d(xz/yz,∥)	→	α-π*(2)
26	27685	361	0.0000517	α-Ni 3d(z²)	→	α-π*(2)
27	27815	360	0.0398972	α/β-π(2)	→	α/β-π*(1)
28	27900	358	0.0000528	α-Ni 3d(z²)	→	α-π*(3)
29	28080	356	0.0130280	α-Ni 3d(xz/yz,∥)	→	α-π*(3)
30	28518	351	0.0088201	α-Ni 3d(xz/yz,∥)	→	α-π*(2)
31	28828	347	0.0041067	α-Ni 3d(xz/yz,⊥)	→	α-π*(3)
32	29706	337	0.1162010	α-Ni 3d(xz/yz,⊥)	→	α-π*(2)
33	30679	326	0.0000027	α-Ni 3d(xy)	→	α-π*(2)
34	30909	324	0.0000251	α-Ni 3d(xy)	→	α-π*(3)
35	31051	322	0.0330242	β-Ni 3d(xz/yz,∥)	→	β-Ph π*(1)
36	31471	318	0.0000043	β-Ni 3d(z²)	→	β-Ph π*(1)
37	31940	313	0.0028909	α/β-π(1)	→	α/β-π*(3)
38	32016	312	0.0042637	β-Ni 3d(xz/yz,∥)	→	β-Ph π*(2)
39	32078	312	0.4313805	α-π(3)	→	α-π*(1)
40	32318	309	0.0000060	α-Ph π*(1)	→	α-π*(1)

Table S11. Absorption transitions for the equilibrium structure of **5** at the TDDFT(B3LYP) level with the CPCM(THF) solvation model.

State	*E* (cm^{-1})	*E* (nm)	f_{osc}	Transition Assignment		
1	9091	1100	0.0002290	β-Ni 3d(z²)	→	β-π*(1)
2	9151	1093	0.0033827	β-Ni 3d(z²)	→	β-Ni 3d(x²-y²)
3	10389	963	0.0004404	β-Ni 3d(xz/yz,⊥)	→	β-Ni 3d(x²-y²)
4	11264	888	0.0030372	β-Ni 3d(xy)	→	β-Ni 3d(x²-y²)
5	11392	878	0.0000812	β-Ni 3d(xz/yz,∥)	→	β-Ni 3d(x²-y²)
6	11860	843	0.0284356	β-Ni 3d(xz/yz,∥)	→	β-π*(1)
7	12658	790	0.0009997	β-Ni 3d(xz/yz,⊥)	→	β-π*(1)
8	15983	626	0.0000290	β-Ni 3d(xy)	→	β-π*(1)
9	16030	624	0.0000045	α-Ni 3d(x²-y²)	→	α-π*(1)
10	17987	556	0.0463427	β-Ni 3d(xz/yz,∥)	→	β-π*(2)
11	18260	548	0.0009906	α-Ni 3d(z²)	→	α-π*(1)
12	18809	532	0.0000022	β-Ni 3d(z²)	→	β-π*(2)
13	19552	512	0.0106762	β-Ni 3d(xz/yz,⊥)	→	β-π*(2)
14	19828	504	0.0246655	α-Ni 3d(xz/yz,⊥)	→	α-π*(1)
15	20886	479	0.0003944	β-Ni 3d(xz/yz,∥)	→	β-π*(3)
16	21168	472	0.0000018	α-Ni 3d(xy)	→	α-π*(1)
17	22193	451	0.0000033	β-Ni 3d(z²)	→	β-π*(3)
18	23219	431	0.0054451	α-Ni 3d(x²-y²)	→	α-π*(2)
19	23248	430	0.1213548	α-Ni 3d(xz/yz,∥)	→	α-π*(1)
20	23662	423	0.0050371	β-Ni 3d(xz/yz,⊥)	→	β-π*(3)
21	24509	408	0.0000006	β-Ni 3d(xy)	→	β-π*(2)
22	25113	398	0.0032781	α/β-π	→	α/β-π*(1)
23	25853	387	0.0000009	α-Ni 3d(z²)	→	α-π*(2)
24	26043	384	0.0000004	α-Ni 3d(x²-y²)	→	α-π*(3)
25	26468	378	0.0399679	α-Ni 3d(xz/yz,∥)	→	α-π*(2)
26	27775	360	0.0000103	β-Ni 3d(xy)	→	β-π*(3)
27	28457	351	0.1106965	α-Ni 3d(xz/yz,⊥)	→	α-π*(2)
28	28589	350	0.0000012	α-Ni 3d(xy)	→	α-π*(2)
29	28780	348	0.0000007	α-Ni 3d(z²)	→	α-π*(3)
30	29229	342	0.0634764	α-Ni 3d(xz/yz,∥)	→	α-π*(3)
31	29675	337	0.0070271	α-Ni 3d(xz/yz,⊥)	→	α-π*(3)
32	30412	329	0.0012561	α/β-π	→	α/β-π*(2)
33	30628	327	0.0023853	α/β-π	→	α/β-π*(3)
34	31416	318	0.0000132	α-Ni 3d(xy)	→	α-π*(3)
35	31968	313	0.3709734	β-Ni 3p(z)	→	β-π*(1)
36	32798	305	0.0000048	β-Ni 3p(x/y,⊥)	→	β-π*(1)
37	32920	304	0.1317423	β-Ni 3d(xz/yz,∥)	→	β-π*(3)

Table S12. Absorption transitions for the equilibrium structure of **5-Br** at the TDDFT(B3LYP) level with the CPCM(THF) solvation model.

State	E (cm^{-1})	E (nm)	f_{osc}	Transition Assignment		
1	8849	1130	0.0024990	β-Ni 3d(z²)	→	β-Ni 3d(x²-y²)
2	9135	1095	0.0000423	β-Ni 3d(xz/yz,⊥)	→	β-Ni 3d(x²-y²)
3	10326	969	0.0005262	β-Ni 3d(z²)	→	β-π*(1)
4	11131	898	0.0000184	β-Ni 3d(xz/yz,∥)	→	β-Ni 3d(x²-y²)
5	11266	888	0.0033695	β-Ni 3d(xy)	→	β-Ni 3d(x²-y²)
6	12189	820	0.0281527	β-Ni 3d(xz/yz,∥)	→	β-π*(1)
7	12595	794	0.0020666	β-Ni 3d(xz/yz,⊥)	→	β-π*(1)
8	16002	625	0.0000073	α-Ni 3d(x²-y²)	→	α-π*(1)
9	16481	607	0.0000518	β-Ni 3d(xy)	→	β-π*(1)
10	18208	549	0.0009511	α-Ni 3d(z²)	→	α-π*(1)
11	18375	544	0.0328136	β-Ni 3d(xz/yz,∥)	→	β-π*(2)
12	18871	530	0.0000601	β-Ni 3d(z²)	→	β-π*(2)
13	19437	515	0.0172895	β-Ni 3d(xz/yz,⊥)	→	β-π*(2)
14	19764	506	0.0326760	α-Ni 3d(xz/yz,⊥)	→	α-π*(1)
15	21205	472	0.0000214	α-Ni 3d(xy)	→	α-π*(1)
16	21301	470	0.0014812	β-Ni 3d(xz/yz,∥)	→	β-π*(3)
17	22197	451	0.0001503	β-Ni 3d(z²)	→	β-π*(3)
18	22899	437	0.1336416	α-Ni 3d(xz/yz,∥)	→	α-π*(1)
19	23179	431	0.0001572	α-Ni 3d(x²-y²)	→	α-π*(2)
20	23632	423	0.0038845	β-Ni 3d(xz/yz,⊥)	→	β-π*(3)
21	24849	402	0.0000008	β-Ni 3d(xy)	→	β-π*(2)
22	25045	399	0.0022873	α/β-π	→	α/β-π*(1)
23	25798	388	0.0001838	α-Ni 3d(z²)	→	α-π*(2)
24	25982	385	0.0010291	α-Ni 3d(x²-y²)	→	α-π*(3)
25	26096	383	0.0375426	α-Ni 3d(xz/yz,∥)	→	α-π*(2)
26	28025	357	0.0010304	β-Ni 3d(xy)	→	β-π*(3)
27	28171	355	0.1092840	α-Ni 3d(xz/yz,⊥)	→	α-π*(2)
28	28615	350	0.0000006	α-Ni 3d(xy)	→	α-π*(2)
29	28719	348	0.0095568	α-Ni 3d(z²)	→	α-π*(3)
30	28794	347	0.0453802	α-Ni 3d(xz/yz,∥)	→	α-π*(3)
31	29330	341	0.0000008	β-Ni 3p(x/y,⊥)	→	β-π*(1)
32	29561	338	0.0029779	α-Ni 3d(xz/yz,⊥)	→	α-π*(3)
33	30407	329	0.0010950	α/β-π	→	α/β-π*(2)
34	30567	327	0.0000111	β-Ni 3p(z)	→	β-π*(1)
35	30605	327	0.0015624	α/β-π	→	α/β-π*(3)
36	31202	321	0.0000006	α-Ni 3p(x/y,⊥)	→	α-π*(1)
37	31406	318	0.0000256	α-Ni 3d(xy)	→	α-π*(3)
38	32009	312	0.4327586	β-π	→	β-π*(1)
39	32588	307	0.0130867	α-Ni 3p(z)	→	α-π*(1)
40	33226	301	0.0321215	β-Ni 3d(xz/yz,∥)	→	β-π*(3)

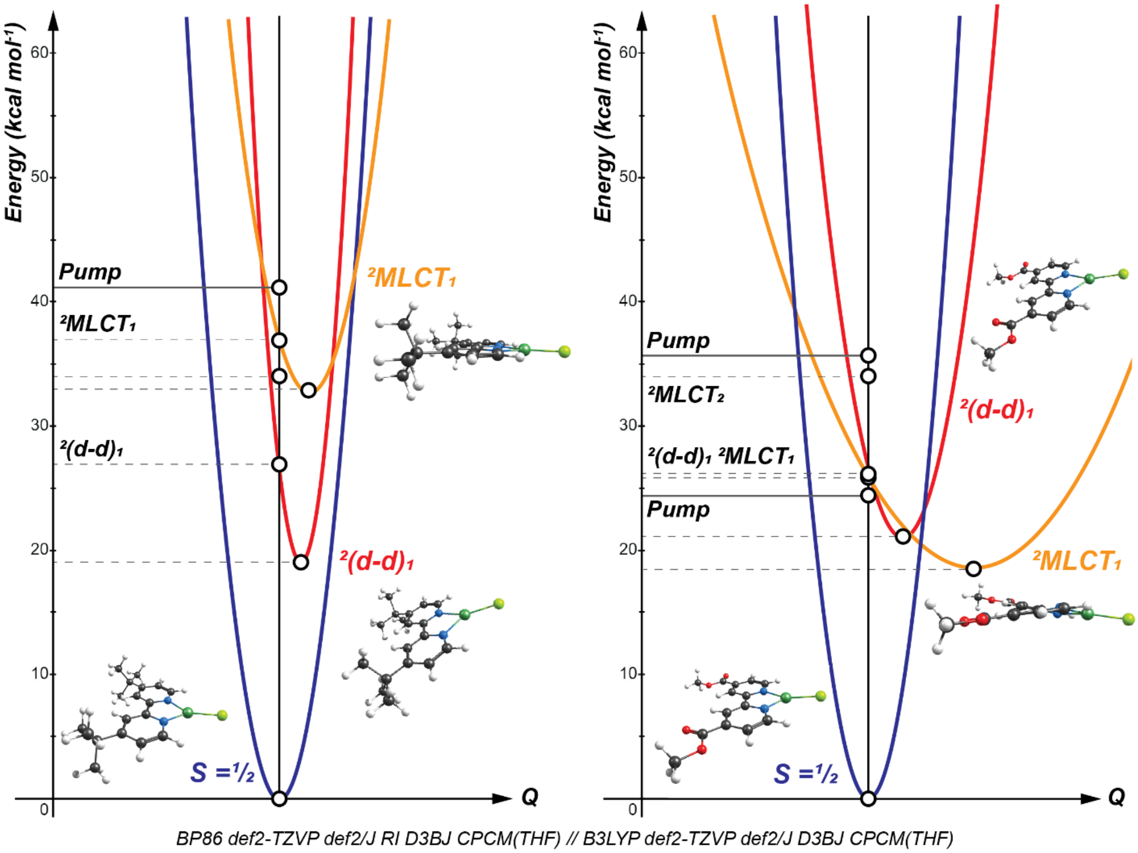

Figure S44. Computed potential energy surfaces for **1** (left) and **5** (right) showing the positions of the calculated transitions relative to the TA pump (800 or 1200 nm). Relaxed structures computed at the DFT BP86 level for the ground state. Lowest energy excited states corresponding to β-Ni 3d(z^2)→β-Ni 3d(x^2–y^2) for the metal centered state (red surface) and β-Ni 3d(z^2)→bpy π*(1) for the MLCT state (orange surface) were optimized using TD-DFT at the B3LYP level; structures are shown. Open circles indicate computed values and correspond to the energy markers on the y-axis. Calculations were performed to investigate the possibility surface crossing into a ligand field excited state and to interrogate the relative geometries of the ^{2}MLCT excited states vs. the ground state structures (see Supporting Information Section 1.6; Fits to Alternative Relaxation Models for more details). We find that only in **1** is there a ^{2}d-d state lower than the MLCT. Given the similar energies of the ^{2}d-d in **1** and the ^{2}MLCT in **5** (the lowest energy surfaces are both ~19 kcal mol^{-1} above the ground state by TD-DFT), one would expect their relaxation times to be similar under energy gap law behavior (a result opposite of that to the experiment). Thus, DFT/TD-DFT suggests that a rate-limiting ^{2}d-d → ^{2}GS is unlikely; relaxation is from the ^{2}MLCT (a point better emphasized by the experimental TA spectra). The ^{2}MLCT geometry of **5** is more distorted than for the ^{2}MLCT of **1**, indicative of an excited-state surface that is nested with the ground state in **1** (Marcus inverted) but a displaced excited-state surface in **5** (Marcus normal). We note, however, that the relative widths and displacements along *Q* of the excited state surfaces are qualitative; accurate computed displacements are precluded without the use of an explicit solvation model to account for outer sphere reorganization energy. Vertical and relaxed excited state energies of **5** are likely to be overestimated (see Supporting Information Section 2.5; Limitations of DFT/TD-DFT).

S2.5 Limitations of DFT/TD-DFT

As can be noted in the experimental versus computed absorption spectra (Figure S43 and Tables S5-S12), TD-DFT well simulates the Ni(I) complexes with electron-rich bpy ligands, particularly those with electron-rich substituents (i.e., **1-3**). However, it quickly fails to adequately stabilize the charge transfer excited states of those with an electron poor bpy ligand or with an extended π-system (i.e., **4-5**). The lowest energy MLCTs of these complexes (labeled Manifold 1 in Figure S43) are either substantially blue-shifted in energy or not accounted for at all.

Taking complex **5** as an example, we attempted to better model the experiment by adjusting the functional used in the TD-DFT calculations[50] and thereby tuning the amount of exact exchange (which can favor open-shell electronic configurations)[51,52] from 20% in B3LYP[46,47] to 10% in meta-hybrid TPSSh[53–55] and 0% in the standard generalized gradient approximation (GGA) functional, BP86.[38,39] However, in none of these cases were we able to reproduce the experimental spectrum of **5** well (Figure S45).

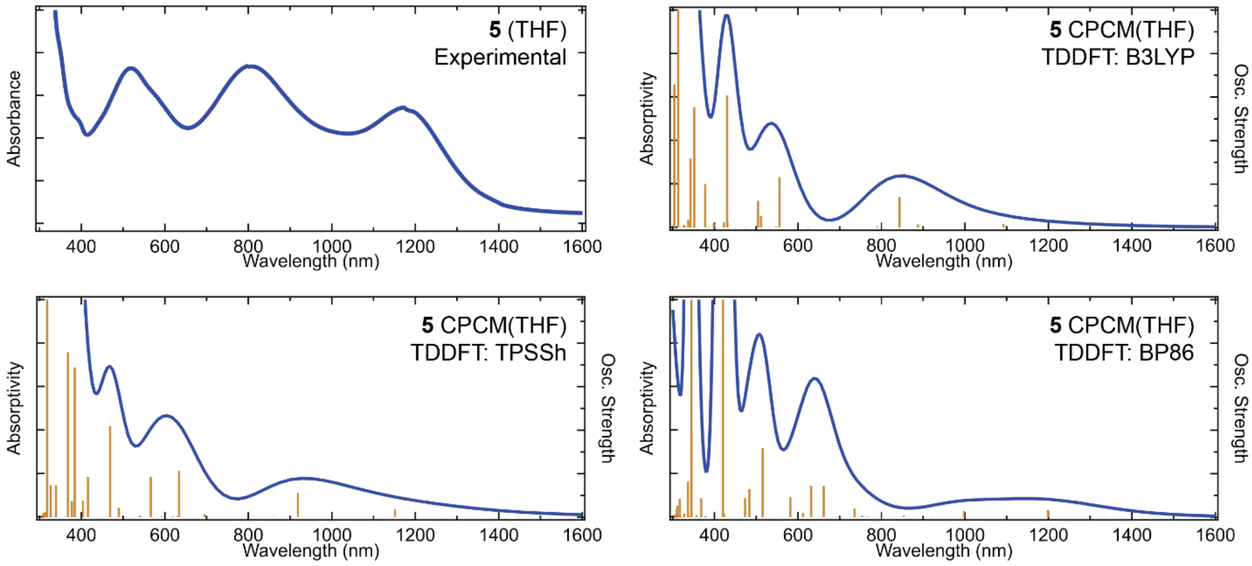

Figure S45. Comparison of the experimental absorption spectrum of **5** in THF (*top left*) and the computed spectra of the same using TD-DFT at the B3LYP (*top right*), TPSSh (*bottom left*), and BP86 (*bottom right*) levels. Although the electronic transitions red-shift, their positions and intensities are still poor models of the experiment.

Excited state optimizations using TD-DFT, particularly those of the MLCT transitions, would similarly suffer. Relaxed MLCT geometries of **4-5** are likely computed too high in energy. Additionally, an implicit solvation model CPCM is used to simulate THF, but it cannot adequately describe outer sphere reorganization energies and related effects that contributing to the experimental value of λ presented in the manuscript.

A detailed computational analysis of these electron-deficient bpy-ligated Ni(I) complexes is needed, likely employing multireference calculations (which have been previously demonstrated to reveal substantial bpy ligand non-innocence effects)[4,49] and/or excited state molecular dynamics, both of which are beyond the scope of this work. As such, the analysis of **1-5** given in the manuscript main text rests on the experimental data; computational analysis of **4-5** is provided here for completeness.

S3. NMR and IR Spectra.

Proton nuclear magnetic resonance (^1H NMR) and fluorine nuclear magnetic resonance (^{19}F NMR) spectra were recorded on a 400 MHz Varian Spectrometer with broadband auto-tune OneProbe. Fluorine NMR were externally referenced to neat fluorobenzene (δ = -113.15 ppm). All ^{13}C NMR spectra were collected on a Bruker AV-III HD 400 MHz spectrometer and were ^1H decoupled. Chemical shifts are reported in parts per million and are referenced to residual solvent signal.

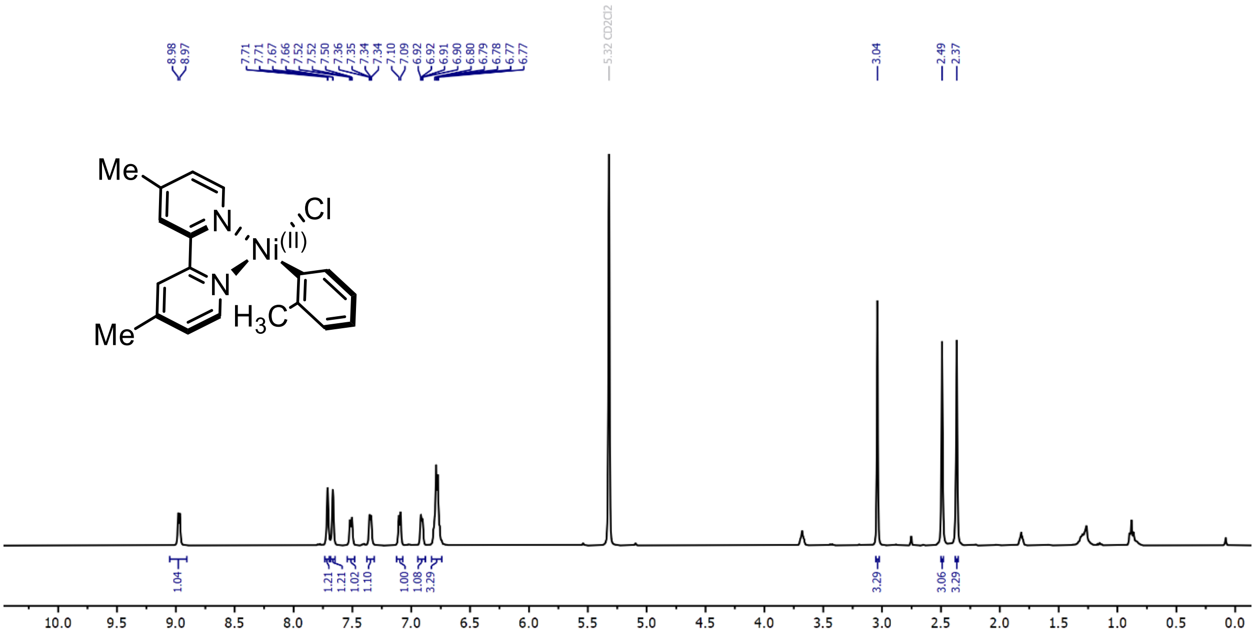

Figure S46. ^1H NMR (400 MHz, CD_2Cl_2) spectra of Ni(^{Me}bpy)(*o*-tolyl)Cl, parent compound for **2**.

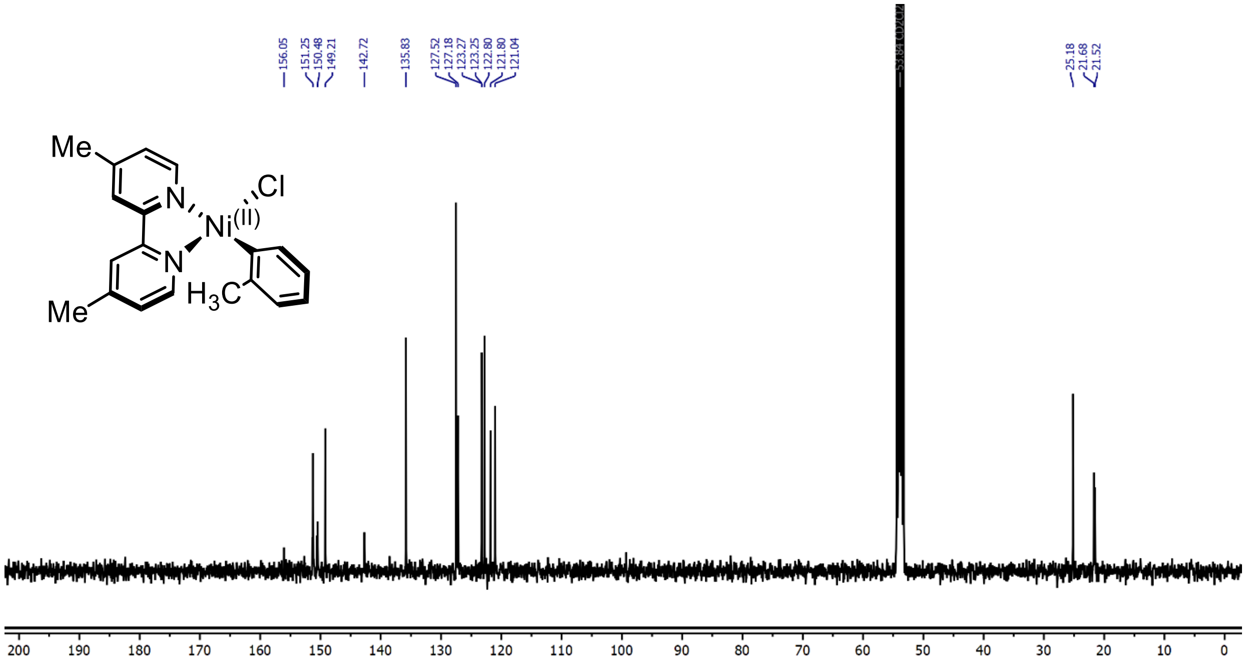

Figure S47. ^{13}C NMR (100 MHz, CD_2Cl_2) spectra of Ni(^{Me}bpy)(*o*-tolyl)Cl, parent compound for **2**.

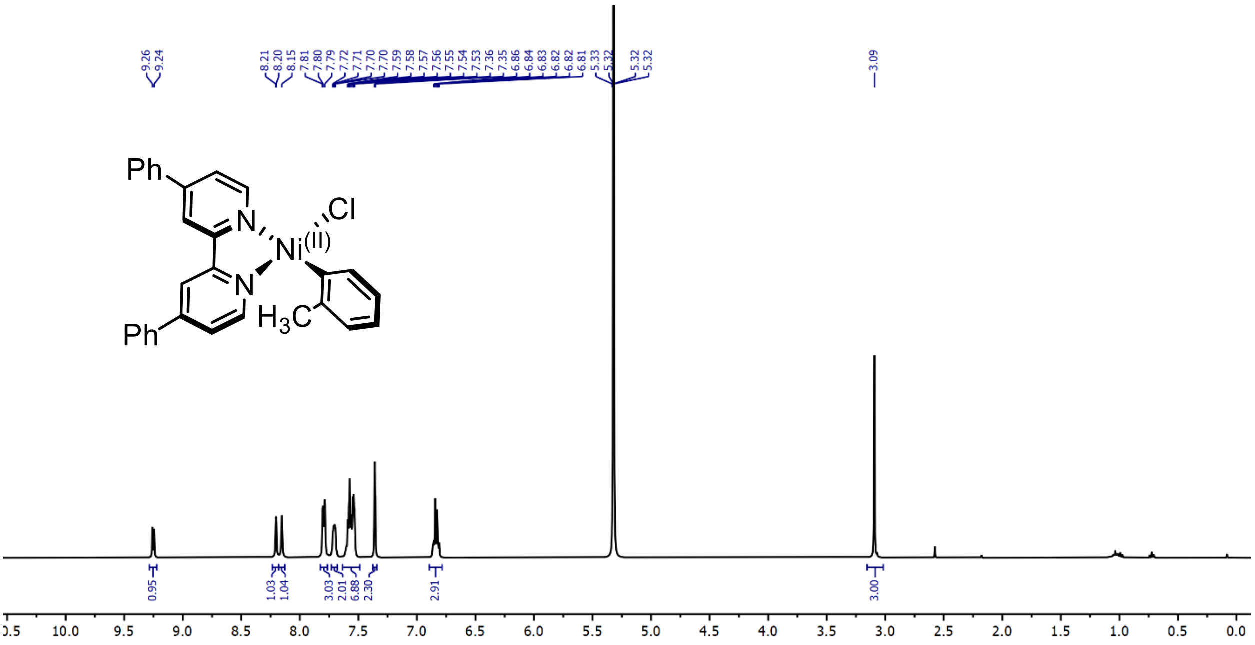

Figure S48. ^{1}H NMR (400 MHz, CD_2Cl_2) spectra of Ni(^{Ph}bpy)(*o*-tolyl)Cl, parent compound for **4**. Proton NMR agrees with previous report.[5]

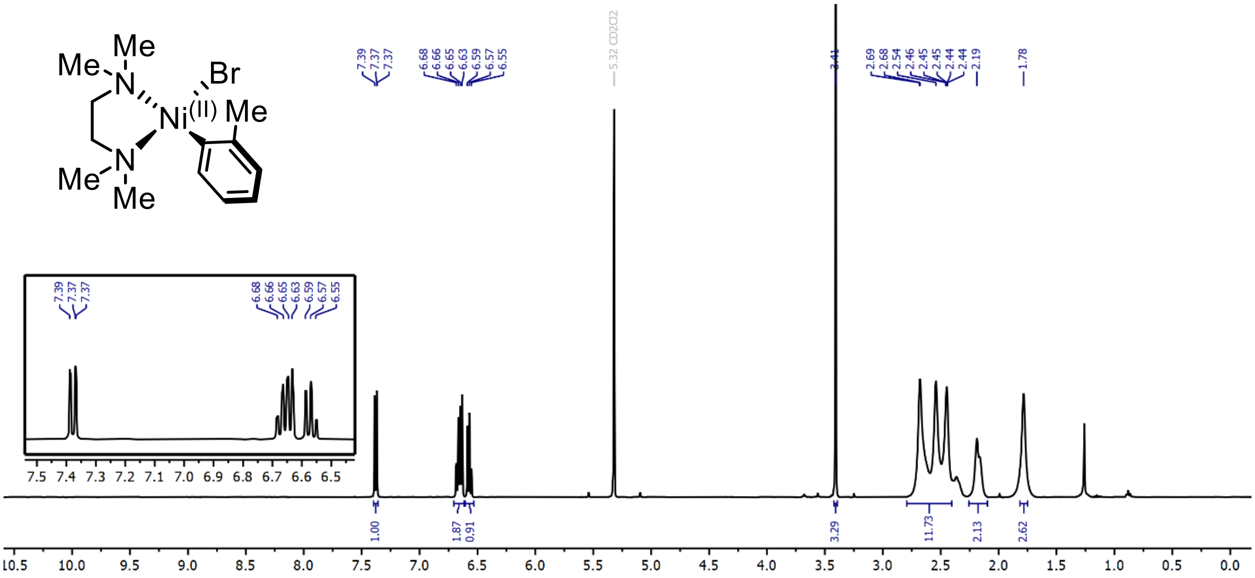

Figure S49. ^{1}H NMR (400 MHz, CD_2Cl_2) spectra of Ni(TMEDA)(*o*-tolyl)Br.

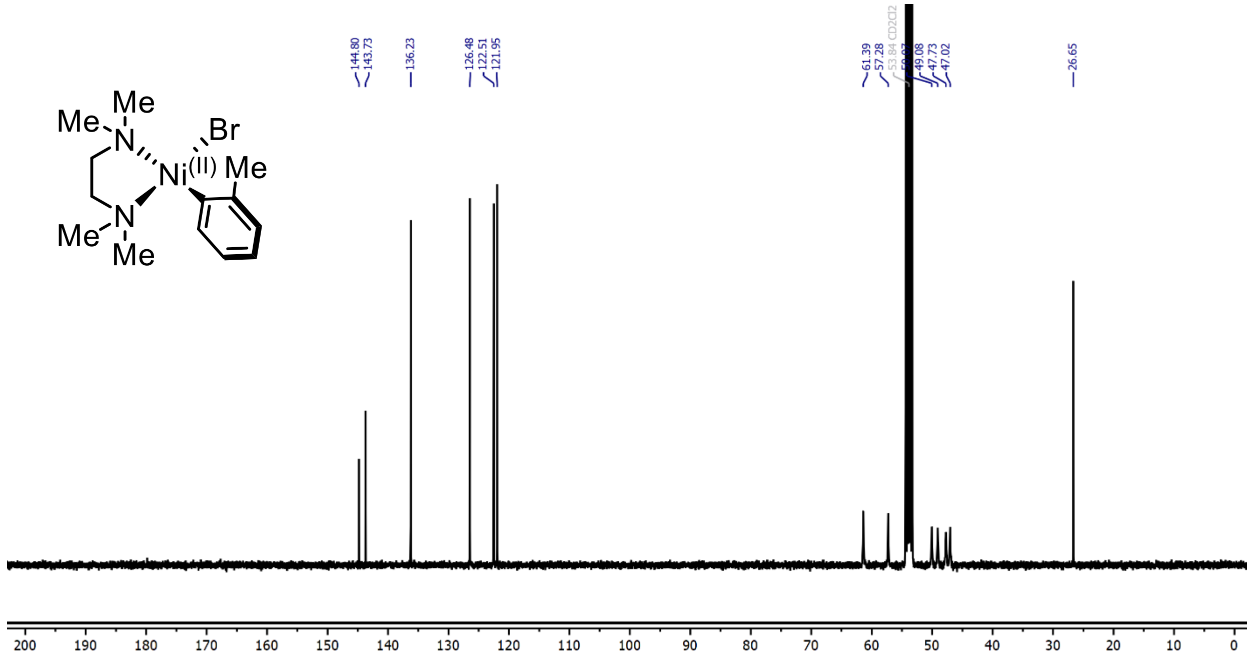

Figure S50. ^{13}C NMR (100 MHz, CD_2Cl_2) spectra of Ni(TMEDA)(*o*-tolyl)Br.

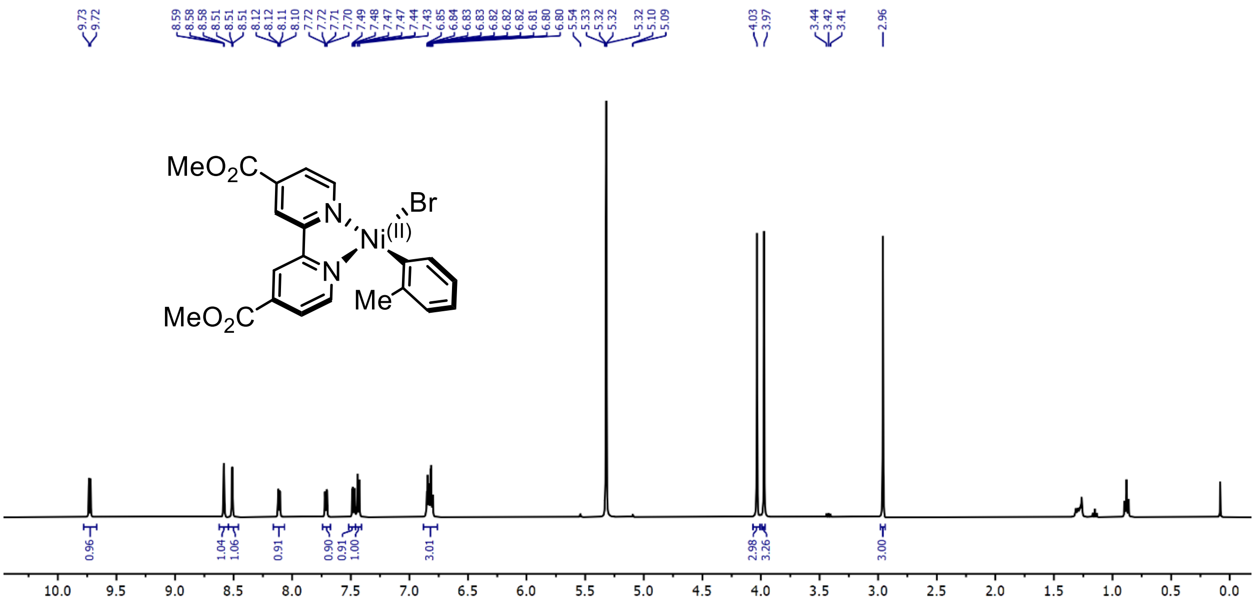

Figure S51. ^{1}H NMR (400 MHz, CD_2Cl_2) spectra of Ni(^{MeO_2C}bpy)(*o*-tolyl)Br, parent compound for **5-Br**.

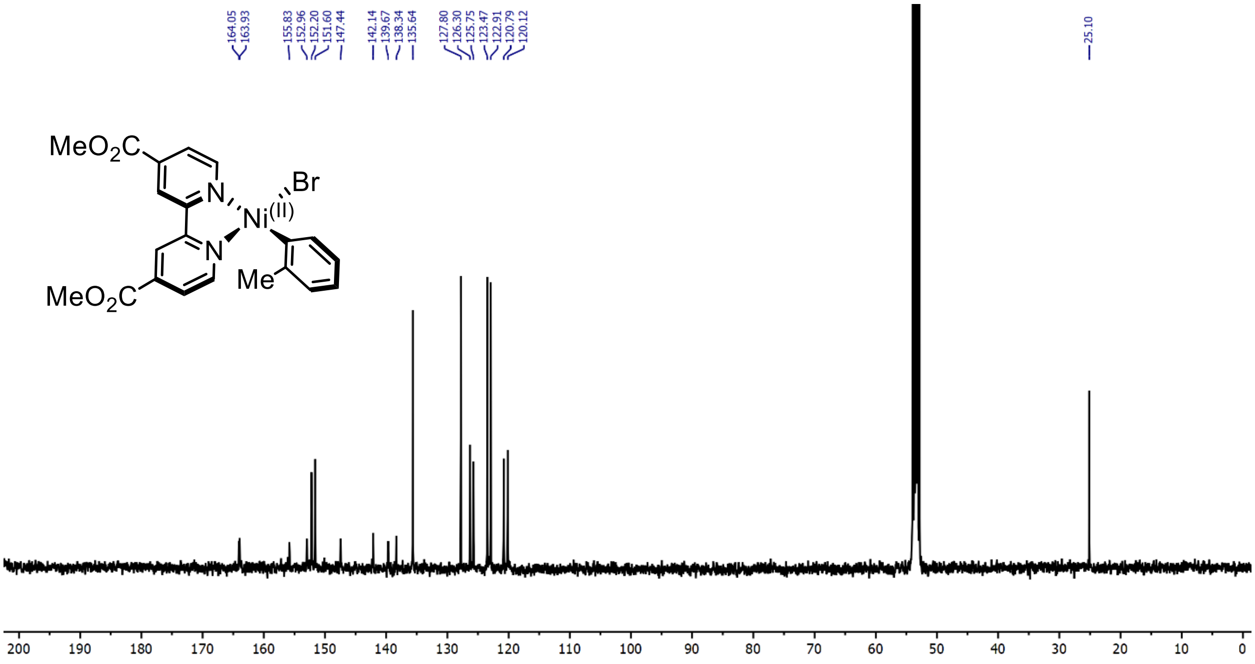

Figure S52. ^{13}C NMR (100 MHz, CD_2Cl_2) spectra of Ni(^{MeO_2C}bpy)(*o*-tolyl)Br, parent compound for **5-Br**.

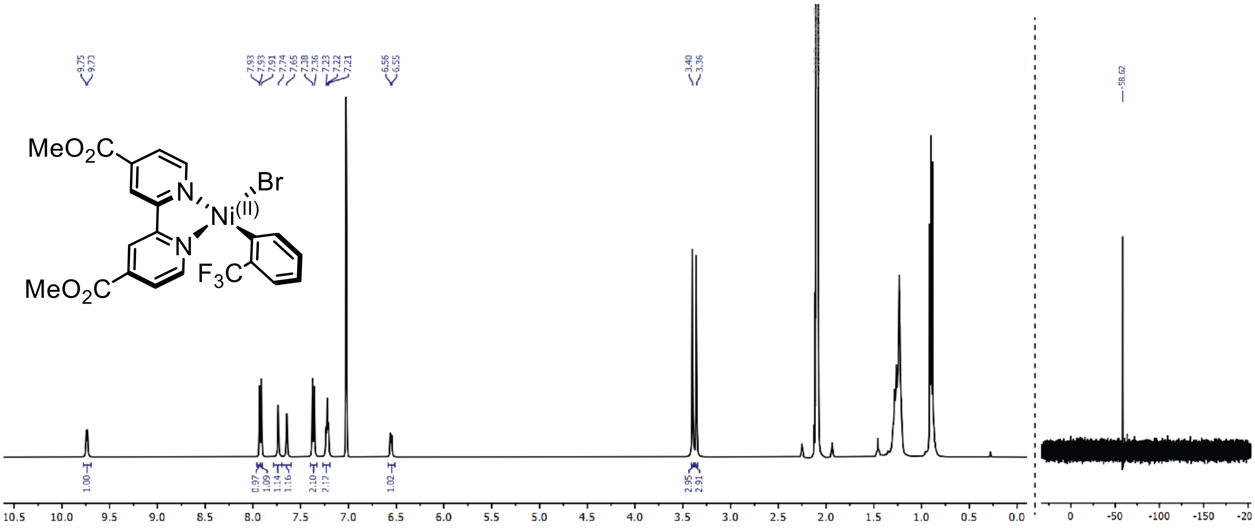

Figure S53. (Left) ^{1}H NMR (400 MHz, d_8-toluene) and (right) ^{19}F NMR (400 MHz, CD_2Cl_2) spectra of Ni(^{MeO_2C}bpy)(*o*-CF_3Ph)Br. Residual hexanes from the workup/washing steps are seen at ~1.26 ppm and ~0.89 ppm in the proton spectrum.

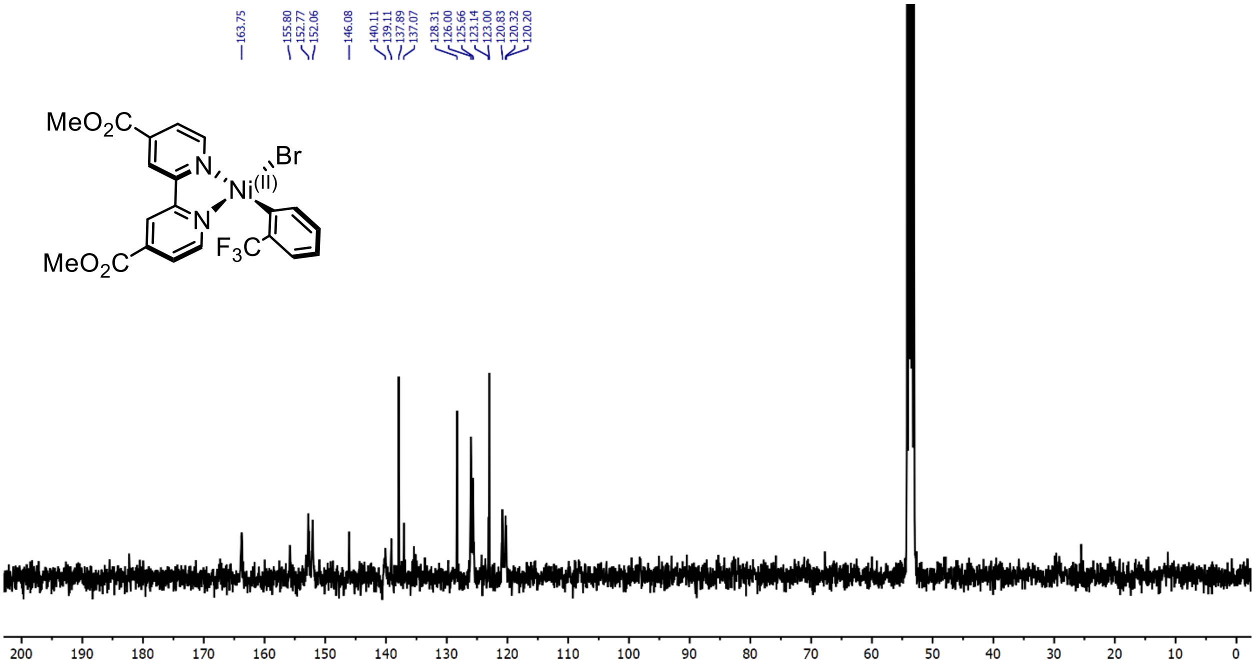

Figure S54. ^{13}C NMR (100 MHz, CD_2Cl_2) spectra of Ni(^{MeO_2C}bpy)(*o*-CF_3Ph)Br.

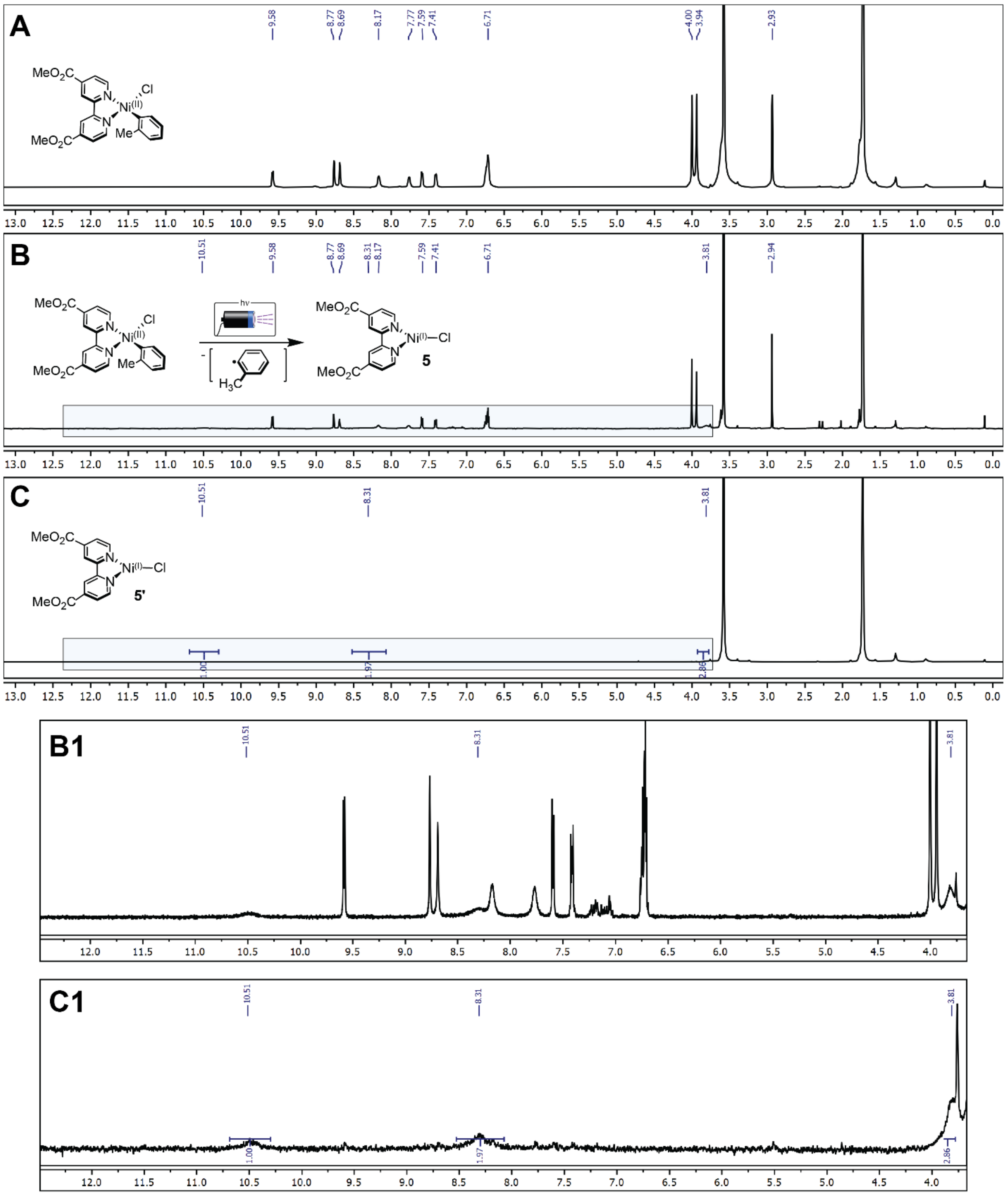

Figure S55. ^{1}H NMR (400 MHz, d_8-THF) spectra of (A) Ni(^{MeO_2C}bpy)(*o*-tolyl)Cl, (B) the photochemical conversion of Ni(^{MeO_2C}bpy)(*o*-tolyl)Cl to **5**, and (C) paramagnetic, isolated **5′**. Boxed insets in (B) and (C) are shown in panels (B1) and (C1), respectively. Assignments for Ni(^{MeO_2C}bpy)(*o*-tolyl)Cl are as described previously.[4]

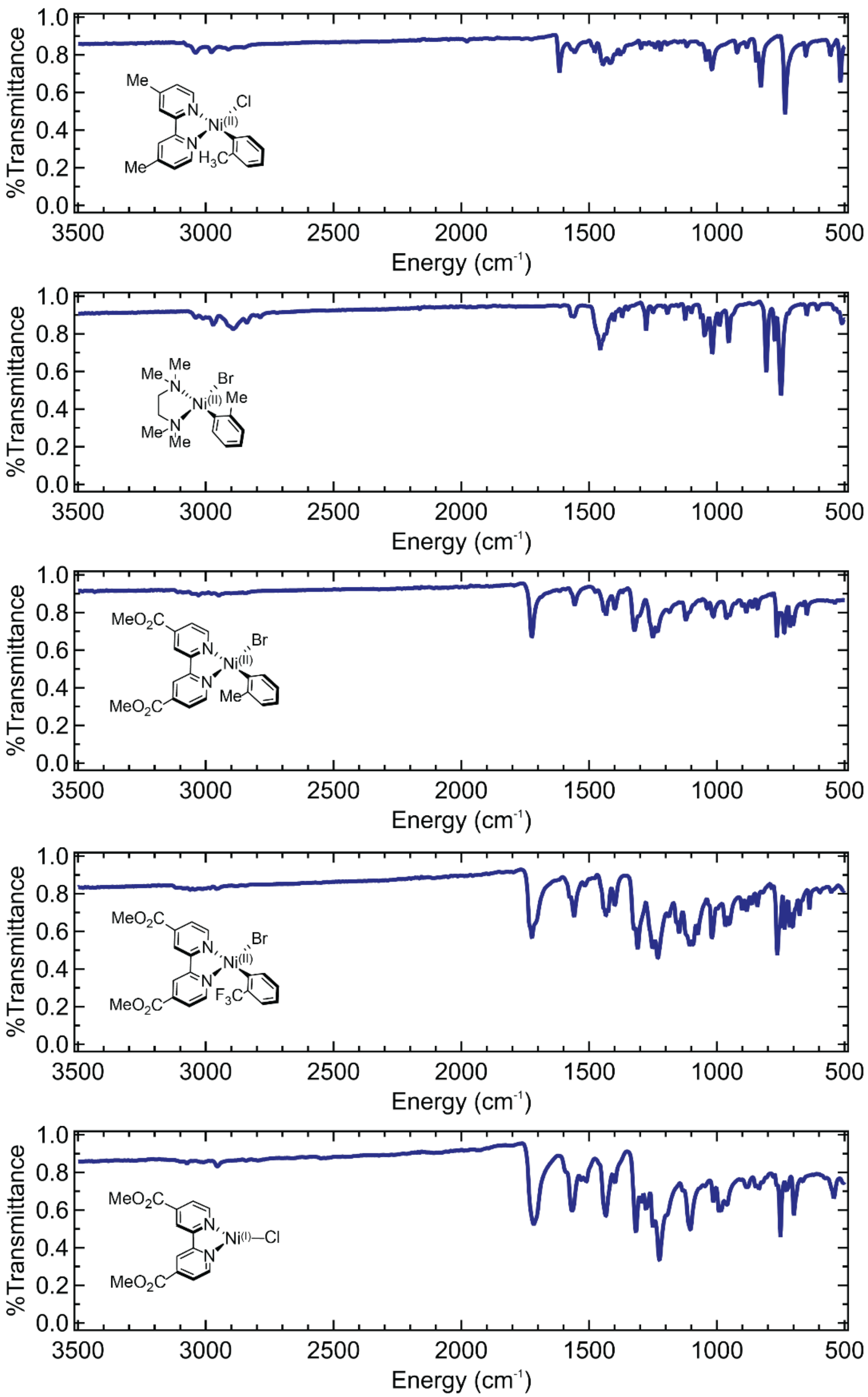

Figure S56. Solid-state IR spectra of the Ni(II) complexes newly synthesized in this work and the isolated complex, **5′**. From top to bottom: Ni(II)(^{Me}bpy)(*o*-tolyl)Cl, Ni(II)(TMEDA)(*o*-tolyl)Cl, Ni(II)(^{MeO_2C}bpy)(*o*-tolyl)Br, Ni(II)(^{MeO_2C}bpy)(*o*-CF_3Ph)Br, Ni(I)(^{MeO_2C}bpy)Cl. Structures shown on each spectrum.

S4. Appendix.

1				1-Br			
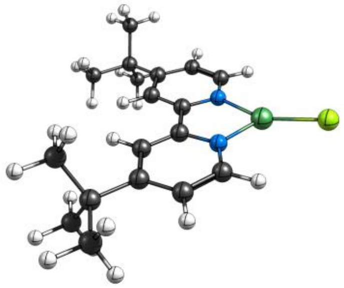				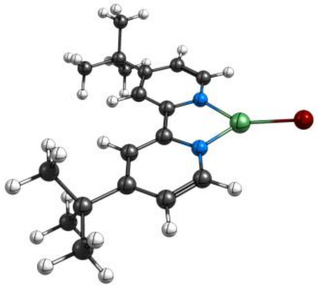			
C	-2.029767481	-5.482378378	0.242513014	C	0.659321000	-2.259714000	0.041788000
N	-2.993320211	-4.541935687	0.229603853	N	-0.397352000	-1.428101000	0.119515000
C	-4.295276591	-4.956603212	0.200439005	C	-1.649222000	-1.968507000	0.012015000
C	-4.630774991	-6.307361731	0.186192007	C	-1.840237000	-3.333722000	-0.178781000
C	-3.635290044	-7.296086104	0.202279473	C	-0.746495000	-4.209229000	-0.260353000
C	-2.306945286	-6.846340471	0.230069863	C	0.526474000	-3.632789000	-0.142727000
C	-5.261779440	-3.849662986	0.190029655	C	-2.727948000	-0.975037000	0.116787000
C	-6.648934787	-4.006354925	0.151789945	C	-4.091702000	-1.272852000	0.071256000
C	-7.493591358	-2.892159547	0.143655115	C	-5.047239000	-0.257758000	0.181630000
C	-6.868329156	-1.631051876	0.179230886	C	-4.557068000	1.053054000	0.333112000
C	-5.485454487	-1.534413023	0.215901240	C	-3.191298000	1.290266000	0.376550000
N	-4.677959590	-2.617770489	0.220269810	N	-2.278114000	0.301427000	0.274279000
C	-9.016340381	-3.002867842	0.098655540	C	-6.550778000	-0.522399000	0.143140000
C	-9.601934114	-2.328966391	1.359270503	C	-7.183911000	-0.000721000	1.452255000
Ni	-2.752347324	-2.623508518	0.259827385	Ni	-0.358827000	0.480878000	0.358578000
Cl	-1.059195981	-1.305289447	0.317631596	Br	1.053673000	2.255198000	0.638329000
C	-4.022766266	-8.773587920	0.191453470	C	-0.974933000	-5.704285000	-0.474611000
C	-4.891752963	-9.069960068	1.434425899	C	-1.869149000	-6.245857000	0.662745000
C	-9.489648427	-4.463198015	0.051075312	C	-6.873605000	-2.016991000	0.001797000
C	-9.532749178	-2.273372247	-1.161574862	C	-7.156813000	0.234846000	-1.059583000
C	-2.793524766	-9.693511954	0.216089041	C	0.342297000	-6.493501000	-0.483851000
C	-4.838551620	-9.067196224	-1.087289670	C	-1.689293000	-5.906205000	-1.829868000
H	-7.065085163	-5.010934268	0.127776804	H	-4.402007000	-2.308624000	-0.046145000
H	-7.454021382	-0.712160239	0.178081633	H	-5.235811000	1.900709000	0.421825000
H	-4.984029087	-0.566301298	0.242288659	H	-2.791397000	2.297545000	0.498717000
H	-5.681886650	-6.592145825	0.165020124	H	-2.854691000	-3.720427000	-0.267245000
H	-1.472701894	-7.544815970	0.242795152	H	1.429217000	-4.238110000	-0.193174000
H	-1.003355313	-5.113796749	0.264629404	H	1.641470000	-1.794106000	0.132408000
H	-3.125386762	-10.741100459	0.204986446	H	0.124197000	-7.560941000	-0.627315000
H	-2.189664978	-9.541107276	1.122413974	H	0.884848000	-6.384022000	0.466347000
H	-2.149507033	-9.535050064	-0.661128316	H	1.004616000	-6.172965000	-1.301145000
H	-10.587640101	-4.487448094	0.014460344	H	-7.964180000	-2.150816000	-0.012640000
H	-9.170794673	-5.023994300	0.941803493	H	-6.474049000	-2.599403000	0.844773000
H	-9.111098939	-4.982162212	-0.841603077	H	-6.471514000	-2.435361000	-0.932513000
H	-9.123131571	-2.731811081	-2.073275273	H	-6.718811000	-0.118922000	-2.004097000
H	-9.255802893	-1.210271219	-1.156192434	H	-6.984190000	1.317268000	-0.983942000
H	-10.629749085	-2.339348662	-1.203057528	H	-8.242662000	0.064168000	-1.095596000
H	-4.243248926	-8.857561688	-1.987679478	H	-1.074874000	-5.521211000	-2.656492000
H	-5.753897280	-8.461049022	-1.131336205	H	-2.660069000	-5.391982000	-1.853258000
H	-5.131020694	-10.127257327	-1.104148959	H	-1.866678000	-6.978306000	-1.999478000
H	-4.332046802	-8.868430762	2.359199251	H	-1.388861000	-6.100021000	1.641047000
H	-5.190577441	-10.128344393	1.433356604	H	-2.040007000	-7.322462000	0.517547000
H	-5.804367177	-8.458291521	1.443432679	H	-2.847755000	-5.747024000	0.681522000
H	-9.250452573	-2.833680152	2.270759038	H	-6.762950000	-0.522027000	2.324143000
H	-10.700034884	-2.384748993	1.333256842	H	-8.269275000	-0.177178000	1.433738000
H	-9.316120791	-1.269916189	1.419775304	H	-7.016833000	1.077236000	1.581444000

1-I				2			
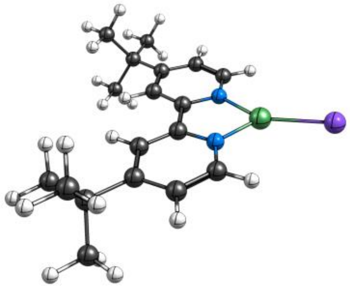				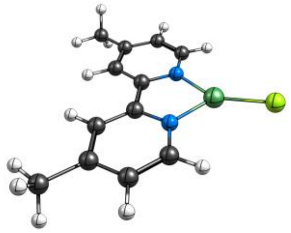			
C	-0.751175000	2.863174000	0.039848000	C	-0.710483000	2.863308000	0.180761000
N	0.216287000	1.927032000	0.046683000	N	0.262518000	1.931301000	0.097130000
C	1.517527000	2.346358000	0.066315000	C	1.559679000	2.351214000	0.045922000
C	1.846986000	3.698260000	0.074519000	C	1.890582000	3.706989000	0.080218000
C	0.847183000	4.683179000	0.064787000	C	0.888165000	4.678474000	0.166719000
C	-0.479159000	4.228310000	0.048675000	C	-0.441423000	4.224544000	0.216764000
C	2.491693000	1.244221000	0.070050000	C	2.527817000	1.250223000	-0.044628000
C	3.877410000	1.409261000	0.118877000	C	3.913083000	1.408225000	-0.114486000
C	4.728096000	0.299192000	0.113278000	C	4.750709000	0.291287000	-0.196705000
C	4.112406000	-0.965072000	0.055949000	C	4.133307000	-0.971932000	-0.205470000
C	2.729961000	-1.070136000	0.016149000	C	2.750849000	-1.068540000	-0.134968000
N	1.918915000	0.008728000	0.024492000	N	1.947017000	0.014365000	-0.055631000
C	6.249761000	0.417804000	0.159594000	Ni	0.023437000	0.013952000	0.046003000
C	6.778836000	-0.367947000	1.379607000	Cl	-1.691356000	-1.276324000	0.095598000
Ni	-0.009358000	0.014083000	0.017072000	H	4.348734000	2.407504000	-0.105601000
I	-1.757424000	-1.706865000	0.005504000	H	4.730038000	-1.882751000	-0.267441000
C	1.227408000	6.162348000	0.068929000	H	2.247241000	-2.035856000	-0.140650000
C	2.055040000	6.462742000	1.338405000	H	2.935772000	4.014392000	0.040845000
C	6.713712000	1.877312000	0.273631000	H	-1.268961000	4.931670000	0.284708000
C	6.831188000	-0.191122000	-1.136123000	H	-1.731723000	2.482348000	0.219264000
C	-0.008786000	7.073668000	0.057297000	C	6.243486000	0.429869000	-0.272213000
C	2.077925000	6.462862000	-1.185246000	H	6.724932000	-0.087009000	0.571548000
H	4.287757000	2.415278000	0.163858000	H	6.630314000	-0.031285000	-1.193411000
H	4.705457000	-1.878916000	0.043982000	H	6.548672000	1.483447000	-0.255454000
H	2.232167000	-2.039731000	-0.022912000	C	1.213724000	6.143338000	0.203972000
H	2.896881000	3.988782000	0.084580000	H	0.817025000	6.607006000	1.119609000
H	-1.316660000	4.922644000	0.041484000	H	2.296930000	6.312533000	0.168362000
H	-1.776107000	2.490317000	0.025775000	H	0.750804000	6.665870000	-0.646746000
H	0.314596000	8.123904000	0.060367000				
H	-0.639190000	6.912120000	0.943558000				
H	-0.622112000	6.912334000	-0.840898000				
H	7.811562000	1.907254000	0.312162000				
H	6.331195000	2.353487000	1.188210000				
H	6.392446000	2.476063000	-0.591113000				
H	6.470584000	0.355448000	-2.019487000				
H	6.550799000	-1.247781000	-1.246572000				
H	7.929022000	-0.129330000	-1.114004000				
H	1.507143000	6.256388000	-2.102118000				
H	2.995528000	5.858824000	-1.208574000				
H	2.368469000	7.523581000	-1.189549000				
H	1.467437000	6.256957000	2.244712000				
H	2.346227000	7.523249000	1.347555000				
H	2.971510000	5.857978000	1.378164000				
H	6.371029000	0.041487000	2.315115000				
H	7.875457000	-0.295007000	1.418533000				
H	6.511232000	-1.431864000	1.323651000				

3				4			
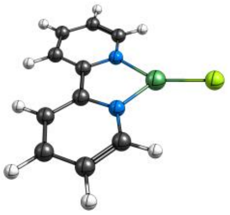				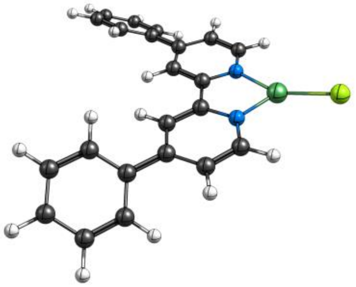			
C	0.473042000	-2.474101000	0.301340000	C	-0.303121000	2.357166000	-1.786324000
N	-0.621213000	-1.686728000	0.241593000	N	0.370314000	1.197550000	-1.632083000
C	-1.820768000	-2.247454000	-0.095944000	C	1.340992000	0.888638000	-2.542159000
C	-1.933067000	-3.610491000	-0.379539000	C	1.643905000	1.733652000	-3.605965000
C	-0.798133000	-4.417499000	-0.316330000	C	0.948392000	2.941798000	-3.774420000
C	0.426311000	-3.837966000	0.030900000	C	-0.049372000	3.239870000	-2.825965000
C	-2.929113000	-1.285073000	-0.124235000	C	2.002125000	-0.397495000	-2.277626000
C	-4.254095000	-1.595831000	-0.438432000	C	3.017789000	-0.943208000	-3.057500000
C	-5.214232000	-0.585292000	-0.427954000	C	3.583328000	-2.184070000	-2.722633000
C	-4.822396000	0.717024000	-0.101945000	C	3.070632000	-2.829197000	-1.580223000
C	-3.487035000	0.963078000	0.200276000	C	2.057866000	-2.233660000	-0.843102000
N	-2.551375000	-0.010033000	0.191995000	N	1.520684000	-1.038404000	-1.170501000
Ni	-0.679100000	0.210618000	0.584668000	Ni	0.120011000	-0.109760000	-0.235981000
Cl	0.725756000	1.738839000	1.122454000	Cl	-1.159087000	-0.274252000	1.478289000
H	-4.532733000	-2.618318000	-0.689887000	H	3.359026000	-0.413421000	-3.945518000
H	-6.252626000	-0.810518000	-0.670461000	H	3.475143000	-3.784416000	-1.247827000
H	-5.539336000	1.537014000	-0.081022000	H	1.652308000	-2.709981000	0.050026000
H	-3.133022000	1.961473000	0.459119000	H	2.409633000	1.443726000	-4.323622000
H	-2.898610000	-4.037241000	-0.647427000	H	-0.614348000	4.169553000	-2.880582000
H	-0.868067000	-5.482974000	-0.534779000	H	-1.064154000	2.569899000	-1.034839000
H	1.337867000	-4.431333000	0.092479000	C	1.252568000	3.851686000	-4.894780000
H	1.403530000	-1.976099000	0.575931000	C	0.244798000	4.652755000	-5.464168000
				C	2.556277000	3.937364000	-5.419065000
				C	0.531524000	5.508562000	-6.527592000
				C	2.842500000	4.797980000	-6.478636000
				C	1.831232000	5.585590000	-7.038813000
				H	-0.776753000	4.584838000	-5.087682000
				H	3.358085000	3.345468000	-4.975335000
				H	-0.264922000	6.113522000	-6.963318000
				H	3.861054000	4.859297000	-6.864606000
				H	2.055069000	6.256808000	-7.869049000
				C	4.663419000	-2.778875000	-3.532174000
				C	4.790750000	-4.176191000	-3.645886000
				C	5.590001000	-1.962084000	-4.207824000
				C	5.809841000	-4.738031000	-4.414914000
				C	6.611721000	-2.525698000	-4.971884000
				C	6.725020000	-3.915699000	-5.080299000
				H	4.070040000	-4.826883000	-3.148912000
				H	5.526701000	-0.877222000	-4.111536000
				H	5.886107000	-5.822953000	-4.500356000
				H	7.327196000	-1.877112000	-5.479460000
				H	7.523016000	-4.355894000	-5.679758000

5				5-Br			
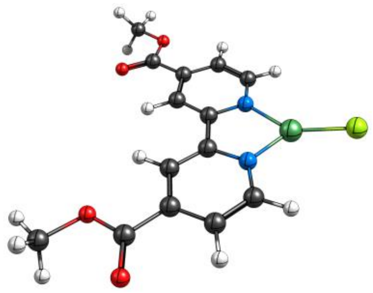				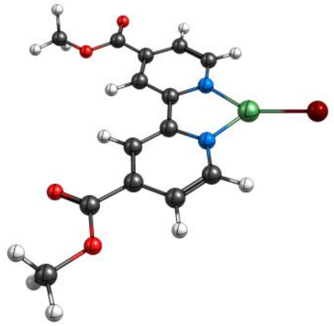			
C	0.568503000	-2.394588000	0.316547000	C	0.764158000	-2.191930000	0.350402000
N	-0.519621000	-1.595903000	0.306684000	N	-0.323481000	-1.393036000	0.341233000
C	-1.753839000	-2.162453000	0.148607000	C	-1.558559000	-1.957846000	0.184071000
C	-1.908807000	-3.537154000	-0.001582000	C	-1.714520000	-3.332451000	0.034530000
C	-0.779763000	-4.362047000	0.010679000	C	-0.586011000	-4.158180000	0.046305000
C	0.483694000	-3.773251000	0.173273000	C	0.677975000	-3.570739000	0.207383000
C	-2.850198000	-1.186377000	0.156934000	C	-2.655609000	-0.981640000	0.191745000
C	-4.199573000	-1.503469000	0.021577000	C	-4.004795000	-1.300035000	0.057076000
C	-5.149743000	-0.476662000	0.047900000	C	-4.955627000	-0.273731000	0.081623000
C	-4.707806000	0.844794000	0.209841000	C	-4.515313000	1.048328000	0.240264000
C	-3.349024000	1.092138000	0.339974000	C	-3.156501000	1.297154000	0.370217000
N	-2.427774000	0.104205000	0.316439000	N	-2.235630000	0.309625000	0.348946000
C	-6.612290000	-0.734410000	-0.088046000	C	-6.418504000	-0.533285000	-0.052459000
O	-6.882910000	-2.048339000	-0.229848000	O	-6.687937000	-1.847857000	-0.187666000
C	-8.286502000	-2.386728000	-0.365605000	C	-8.091449000	-2.188373000	-0.320409000
Ni	-0.529882000	0.317468000	0.498309000	Ni	-0.337623000	0.517928000	0.540196000
Cl	0.960350000	1.824056000	0.786525000	C	-0.785026000	-5.628340000	-0.112243000
C	-0.977021000	-5.831976000	-0.149263000	O	0.381935000	-6.301605000	-0.075498000
O	0.190825000	-6.504183000	-0.112213000	C	0.278198000	-7.741378000	-0.218972000
C	0.088721000	-7.943740000	-0.257699000	O	-7.269897000	0.343453000	-0.040063000
O	-7.463500000	0.142614000	-0.071618000	O	-1.878068000	-6.156553000	-0.256796000
O	-2.069176000	-6.361714000	-0.295546000	H	-4.320438000	-2.333754000	-0.064544000
H	-4.515737000	-2.536785000	-0.102283000	H	-5.231610000	1.867955000	0.262336000
H	-5.423238000	1.665127000	0.233770000	H	-2.770426000	2.308504000	0.497451000
H	-2.962519000	2.103126000	0.468761000	H	-2.698855000	-3.780523000	-0.091230000
H	-2.892830000	-3.985669000	-0.128370000	H	1.582467000	-4.174833000	0.221837000
H	1.388707000	-4.376553000	0.188369000	H	1.723792000	-1.690908000	0.478182000
H	1.527777000	-1.892921000	0.444695000	H	-8.643445000	-1.862308000	0.569907000
H	-8.839144000	-2.065172000	0.525972000	H	-8.512503000	-1.709724000	-1.213329000
H	-8.706483000	-1.902810000	-1.256203000	H	-8.114985000	-3.277492000	-0.415395000
H	-8.311075000	-3.475297000	-0.466662000	H	-0.328688000	-8.157626000	0.594718000
H	-0.519047000	-8.361820000	0.554406000	H	-0.175988000	-7.991643000	-1.185777000
H	-0.363635000	-8.193319000	-1.225558000	H	1.306252000	-8.110018000	-0.165190000
H	1.117044000	-8.311511000	-0.202772000	Br	1.211822000	2.149558000	0.887246000

S5. References

(1) Stoll, S.; Schweiger, A. EasySpin, a Comprehensive Software Package for Spectral Simulation and Analysis in EPR. *J. Magn. Reson.* **2006**, *178* (1), 42–55. https://doi.org/10.1016/j.jmr.2005.08.013.

(2) Kazmierczak, N. P.; Chew, J. A.; Vander Griend, D. A. Bootstrap Methods for Quantifying the Uncertainty of Binding Constants in the Hard Modeling of Spectrophotometric Titration Data. *Anal. Chim. Acta* **2022**, *1227*, 339834. https://doi.org/10.1016/j.aca.2022.339834.

(3) Shields, B. J.; Kudisch, B.; Scholes, G. D.; Doyle, A. G. Long-Lived Charge-Transfer States of Nickel(II) Aryl Halide Complexes Facilitate Bimolecular Photoinduced Electron Transfer. *J. Am. Chem. Soc.* **2018**, *140* (8), 3035–3039. https://doi.org/10.1021/jacs.7b13281.

(4) Cagan, D. A.; Bím, D.; Silva, B.; Kazmierczak, N. P.; McNicholas, B. J.; Hadt, R. G. Elucidating the Mechanism of Excited-State Bond Homolysis in Nickel–Bipyridine Photoredox Catalysts. *J. Am. Chem. Soc.* **2022**, *144* (14), 6516–6531. https://doi.org/10.1021/jacs.2c01356.

(5) Ting, S. I.; Garakyaraghi, S.; Taliaferro, C. M.; Shields, B. J.; Scholes, G. D.; Castellano, F. N.; Doyle, A. G. 3d-d Excited States of Ni(II) Complexes Relevant to Photoredox Catalysis: Spectroscopic Identification and Mechanistic Implications. *J. Am. Chem. Soc.* **2020**, *142* (12), 5800–5810. https://doi.org/10.1021/jacs.0c00781.

(6) Cagan, D. A.; Bím, D.; McNicholas, B. J.; Kazmierczak, N. P.; Oyala, P. H.; Hadt, R. G. Photogenerated Ni(I)–Bipyridine Halide Complexes: Structure–Function Relationships for Competitive C(Sp2)–Cl Oxidative Addition and Dimerization Reactivity Pathways. *Inorg. Chem.* **2023**, *62* (24), 9538–9551. https://doi.org/10.1021/acs.inorgchem.3c00917.

(7) Ting, S. I.; Williams, W. L.; Doyle, A. G. Oxidative Addition of Aryl Halides to a Ni(I)-Bipyridine Complex. *J. Am. Chem. Soc.* **2022**, *144* (12), 5575–5582. https://doi.org/10.1021/jacs.2c00462.

(8) Sheldrick, G. M. Phase Annealing in SHELX-90: Direct Methods for Larger Structures. *Acta Crystallogr. A* **1990**, *46* (6), 467–473. https://doi.org/10.1107/S0108767390000277.

(9) Sheldrick, G. M. Crystal Structure Refinement with SHELXL. *Acta Crystallogr. Sect. C Struct. Chem.* **2015**, *71* (1), 3–8. https://doi.org/10.1107/S2053229614024218.

(10) Müller, P. Practical Suggestions for Better Crystal Structures. *Crystallogr. Rev.* **2009**, *15* (1), 57–83. https://doi.org/10.1080/08893110802547240.

(11) Cagan, D. A.; Bím, D.; McNicholas, B. J.; Kazmierczak, N. P.; Oyala, P. H.; Hadt, R. G. Photogenerated Ni(I)–Bipyridine Halide Complexes: Structure–Function Relationships for Competitive C(Sp2)–Cl Oxidative Addition and Dimerization Reactivity Pathways. *Inorg. Chem.* **2023**, *62* (24), 9538–9551. https://doi.org/10.1021/acs.inorgchem.3c00917.

(12) Newman-Stonebraker, S. H.; Raab, T. J.; Roshandel, H.; Doyle, A. G. Synthesis of Nickel(I)–Bromide Complexes via Oxidation and Ligand Displacement: Evaluation of Ligand Effects on Speciation and Reactivity. *J. Am. Chem. Soc.* **2023**, *145* (35), 19368–19377. https://doi.org/10.1021/jacs.3c06233.

(13) Dawson, G. A.; Lin, Q.; Neary, M. C.; Diao, T. Ligand Redox Activity of Organonickel Radical Complexes Governed by the Geometry. *J. Am. Chem. Soc.* **2023**. https://doi.org/10.1021/jacs.3c07031.

(14) Diccianni, J. B.; Diao, T. Mechanisms of Nickel-Catalyzed Cross-Coupling Reactions. *Trends Chem.* **2019**, *1* (9), 830–844. https://doi.org/10.1016/j.trechm.2019.08.004.

(15) Kunnus, K.; Li, L.; Titus, C. J.; Lee, S. J.; Reinhard, M. E.; Koroidov, S.; Kjær, K. S.; Hong, K.; Ledbetter, K.; Doriese, W. B.; O'Neil, G. C.; Swetz, D. S.; Ullom, J. N.; Li, D.;

Irwin, K.; Nordlund, D.; Cordones, A. A.; Gaffney, K. J. Chemical Control of Competing Electron Transfer Pathways in Iron Tetracyano-Polypyridyl Photosensitizers. *Chem. Sci.* **2020**, *11* (17), 4360–4373. https://doi.org/10.1039/C9SC06272F.
(16) Sun, R.; Qin, Y.; Ruccolo, S.; Schnedermann, C.; Costentin, C.; Daniel G. Nocera. Elucidation of a Redox-Mediated Reaction Cycle for Nickel-Catalyzed Cross Coupling. *J. Am. Chem. Soc.* **2019**, *141* (1), 89–93. https://doi.org/10.1021/jacs.8b11262.
(17) Mohadjer Beromi, M.; Brudvig, G. W.; Hazari, N.; Lant, H. M. C.; Mercado, B. Q. Synthesis and Reactivity of Paramagnetic Nickel Polypyridyl Complexes Relevant to C(Sp2)–C(Sp3)Coupling Reactions. *Angew. Chem. Int. Ed.* **2019**, *58* (18), 6094–6098. https://doi.org/10.1002/anie.201901866.
(18) Till, N. A.; Oh, S.; MacMillan, D. W. C.; Bird, M. J. The Application of Pulse Radiolysis to the Study of Ni(I) Intermediates in Ni-Catalyzed Cross-Coupling Reactions. *J. Am. Chem. Soc.* **2021**, *143* (25), 9332–9337. https://doi.org/10.1021/jacs.1c04652.
(19) Englman, R.; Jortner, J. The Energy Gap Law for Radiationless Transitions in Large Molecules. *Mol. Phys.* **1970**, *18* (2), 145–164. https://doi.org/10.1080/00268977000100171.
(20) Freed, K. F.; Jortner, J. Multiphonon Processes in the Nonradiative Decay of Large Molecules. *J. Chem. Phys.* **1970**, *52* (12), 6272–6291. https://doi.org/10.1063/1.1672938.
(21) Byrne, J. P.; McCoy, E. F.; Ross, I. G. Internal Conversion in Aromatic and N-Heteroaromatic Molecules. *Aust. J. Chem.* **1965**, *18* (10), 1589–1603. https://doi.org/10.1071/ch9651589.
(22) Marcus, R. A. Chemical and Electrochemical Electron-Transfer Theory. *Annu. Rev. Phys. Chem.* **1964**, *15* (1), 155–196. https://doi.org/10.1146/annurev.pc.15.100164.001103.
(23) Marcus, R. A. On the Theory of Oxidation-Reduction Reactions Involving Electron Transfer. I. *J. Chem. Phys.* **1956**, *24* (5), 966–978. https://doi.org/10.1063/1.1742723.
(24) Marcus, R. A. Theoretical Relations among Rate Constants, Barriers, and Broensted Slopes of Chemical Reactions. *J. Phys. Chem.* **1968**, *72* (3), 891–899. https://doi.org/10.1021/j100849a019.
(25) Marcus, R. A.; Sutin, N. Electron Transfers in Chemistry and Biology. *Biochim. Biophys. Acta BBA - Rev. Bioenerg.* **1985**, *811* (3), 265–322. https://doi.org/10.1016/0304-4173(85)90014-X.
(26) Siders, P.; Marcus, R. A. Quantum Effects for Electron-Transfer Reactions in the "Inverted Region." *J. Am. Chem. Soc.* **1981**, *103* (4), 748–752. https://doi.org/10.1021/ja00394a004.
(27) Ulstrup, J.; Jortner, J. The Effect of Intramolecular Quantum Modes on Free Energy Relationships for Electron Transfer Reactions. *J. Chem. Phys.* **1975**, *63* (10), 4358–4368. https://doi.org/10.1063/1.431152.
(28) Kumpulainen, T.; Lang, B.; Rosspeintner, A.; Vauthey, E. Ultrafast Elementary Photochemical Processes of Organic Molecules in Liquid Solution. *Chem. Rev.* **2017**, *117* (16), 10826–10939. https://doi.org/10.1021/acs.chemrev.6b00491.
(29) Closs, G. L.; Miller, J. R. Intramolecular Long-Distance Electron Transfer in Organic Molecules. *Science* **1988**, *240* (4851), 440–447. https://doi.org/10.1126/science.240.4851.440.
(30) Mataga, N.; Chosrowjan, H.; Shibata, Y.; Yoshida, N.; Osuka, A.; Kikuzawa, T.; Okada, T. First Unequivocal Observation of the Whole Bell-Shaped Energy Gap Law in Intramolecular Charge Separation from S2 Excited State of Directly Linked Porphyrin−Imide Dyads and Its Solvent-Polarity Dependencies. *J. Am. Chem. Soc.* **2001**, *123* (49), 12422–12423. https://doi.org/10.1021/ja010865s.

(31) Häberle, T.; Hirsch, J.; Pöllinger, F.; Heitele, H.; Michel-Beyerle, M. E.; Anders, C.; Döhling, A.; Krieger, C.; Rückemann, A.; Staab, H. A. Ultrafast Charge Separation and Driving Force Dependence in Cyclophane-Bridged Zn−Porphyrin−Quinone Molecules. *J. Phys. Chem.* **1996**, *100* (46), 18269–18274. https://doi.org/10.1021/jp960423g.
(32) Asahi, T.; Ohkohchi, M.; Matsusaka, R.; Mataga, N.; Zhang, R. P.; Osuka, A.; Maruyama, K. Intramolecular Photoinduced Charge Separation and Charge Recombination of the Product Ion Pair States of a Series of Fixed-Distance Dyads of Porphyrins and Quinones: Energy Gap and Temperature Dependences of the Rate Constants. *J. Am. Chem. Soc.* **1993**, *115* (13), 5665–5674. https://doi.org/10.1021/ja00066a036.
(33) Poronik, Y. M.; Sadowski, B.; Szychta, K.; Quina, F. H.; Vullev, V. I.; Gryko, D. T. Revisiting the Non-Fluorescence of Nitroaromatics: Presumption *versus* Reality. *J. Mater. Chem. C* **2022**, *10* (8), 2870–2904. https://doi.org/10.1039/D1TC05423F.
(34) Miller, J. R.; Calcaterra, L. T.; Closs, G. L. Intramolecular Long-Distance Electron Transfer in Radical Anions. The Effects of Free Energy and Solvent on the Reaction Rates. *J. Am. Chem. Soc.* **1984**, *106* (10), 3047–3049. https://doi.org/10.1021/ja00322a058.
(35) Heitele, H.; Poellinger, F.; Haeberle, T.; Michel-Beyerle, M. E.; Staab, H. A. Energy Gap and Temperature Dependence of Photoinduced Electron Transfer in Porphyrin-Quinone Cyclophanes. *J. Phys. Chem.* **1994**, *98* (30), 7402–7410. https://doi.org/10.1021/j100081a028.
(36) Neese, F. The ORCA Program System. *WIREs Comput. Mol. Sci.* **2012**, *2* (1), 73–78. https://doi.org/10.1002/wcms.81.
(37) Neese, F. Software Update: The ORCA Program System—Version 5.0. *WIREs Comput. Mol. Sci.* **2022**, *12* (5), e1606. https://doi.org/10.1002/wcms.1606.
(38) Perdew, J. P. Density-Functional Approximation for the Correlation Energy of the Inhomogeneous Electron Gas. *Phys. Rev. B* **1986**, *33* (12), 8822–8824. https://doi.org/10.1103/PhysRevB.33.8822.
(39) Becke, A. D. Density-Functional Exchange-Energy Approximation with Correct Asymptotic Behavior. *Phys. Rev. A* **1988**, *38* (6), 3098–3100. https://doi.org/10.1103/PhysRevA.38.3098.
(40) Weigend, F.; Ahlrichs, R. Balanced Basis Sets of Split Valence, Triple Zeta Valence and Quadruple Zeta Valence Quality for H to Rn: Design and Assessment of Accuracy. *Phys. Chem. Chem. Phys.* **2005**, *7* (18), 3297–3305. https://doi.org/10.1039/B508541A.
(41) Eichkorn, K.; Treutler, O.; Öhm, H.; Häser, M.; Ahlrichs, R. Auxiliary Basis Sets to Approximate Coulomb Potentials. *Chem. Phys. Lett.* **1995**, *240* (4), 283–290. https://doi.org/10.1016/0009-2614(95)00621-A.
(42) Grimme, S.; Antony, J.; Ehrlich, S.; Krieg, H. A Consistent and Accurate Ab Initio Parametrization of Density Functional Dispersion Correction (DFT-D) for the 94 Elements H-Pu. *J. Chem. Phys.* **2010**, *132* (15), 154104. https://doi.org/10.1063/1.3382344.
(43) Grimme, S.; Ehrlich, S.; Goerigk, L. Effect of the Damping Function in Dispersion Corrected Density Functional Theory. *J. Comput. Chem.* **2011**, *32* (7), 1456–1465. https://doi.org/10.1002/jcc.21759.
(44) Klamt, A.; Schüürmann, G. COSMO: A New Approach to Dielectric Screening in Solvents with Explicit Expressions for the Screening Energy and Its Gradient. *J. Chem. Soc. Perkin Trans. 2* **1993**, No. 5, 799–805. https://doi.org/10.1039/P29930000799.
(45) Barone, V.; Cossi, M. Quantum Calculation of Molecular Energies and Energy Gradients in Solution by a Conductor Solvent Model. *J. Phys. Chem. A* **1998**, *102* (11), 1995–2001. https://doi.org/10.1021/jp9716997.

(46) Lee, C.; Yang, W.; Parr, R. G. Development of the Colle-Salvetti Correlation-Energy Formula into a Functional of the Electron Density. *Phys. Rev. B* **1988**, *37* (2), 785–789. https://doi.org/10.1103/PhysRevB.37.785.

(47) Becke, A. D. Density-functional Thermochemistry. III. The Role of Exact Exchange. *J. Chem. Phys.* **1993**, *98* (7), 5648–5652. https://doi.org/10.1063/1.464913.

(48) Neese, F.; Wennmohs, F.; Hansen, A.; Becker, U. Efficient, Approximate and Parallel Hartree–Fock and Hybrid DFT Calculations. A 'Chain-of-Spheres' Algorithm for the Hartree–Fock Exchange. *Chem. Phys.* **2009**, *356* (1), 98–109. https://doi.org/10.1016/j.chemphys.2008.10.036.

(49) Cagan, D. A.; Stroscio, G. D.; Cusumano, A. Q.; Hadt, R. G. Multireference Description of Nickel–Aryl Homolytic Bond Dissociation Processes in Photoredox Catalysis. *J. Phys. Chem. A* **2020**, *124* (48), 9915–9922. https://doi.org/10.1021/acs.jpca.0c08646.

(50) Jensen, K. P.; Roos, B. O.; Ryde, U. Performance of Density Functionals for First Row Transition Metal Systems. *J. Chem. Phys.* **2007**, *126* (1), 014103. https://doi.org/10.1063/1.2406071.

(51) Neese, F. A Critical Evaluation of DFT, Including Time-Dependent DFT, Applied to Bioinorganic Chemistry. *JBIC J. Biol. Inorg. Chem.* **2006**, *11* (6), 702–711. https://doi.org/10.1007/s00775-006-0138-1.

(52) Jensen, K. P. Bioinorganic Chemistry Modeled with the TPSSh Density Functional. *Inorg. Chem.* **2008**, *47* (22), 10357–10365. https://doi.org/10.1021/ic800841t.

(53) Perdew, J. P.; Kurth, S.; Zupan, A.; Blaha, P. Accurate Density Functional with Correct Formal Properties: A Step Beyond the Generalized Gradient Approximation. *Phys. Rev. Lett.* **1999**, *82* (12), 2544–2547. https://doi.org/10.1103/PhysRevLett.82.2544.

(54) Perdew, J. P.; Tao, J.; Staroverov, V. N.; Scuseria, G. E. Meta-Generalized Gradient Approximation: Explanation of a Realistic Nonempirical Density Functional. *J. Chem. Phys.* **2004**, *120* (15), 6898–6911. https://doi.org/10.1063/1.1665298.

(55) Staroverov, V. N.; Scuseria, G. E.; Tao, J.; Perdew, J. P. Comparative Assessment of a New Nonempirical Density Functional: Molecules and Hydrogen-Bonded Complexes. *J. Chem. Phys.* **2003**, *119* (23), 12129–12137. https://doi.org/10.1063/1.1626543.
